# Prolonged intermittent theta burst stimulation enhances hippocampal plasticity via GluN2A-mediated signaling

**DOI:** 10.3389/fnagi.2026.1757554

**Published:** 2026-03-09

**Authors:** Danica Popovic, Marina Zaric Kontic, Milica Zeljkovic Jovanovic, Milena Milosevic, Teodora Martic, Tamara Radukic, Andjela Stekic, Emilija Glavonic, Ana Jakovljevic, Katarina Mihajlovic, Marija Adzic Bukvic, Ivana Stevanovic, Milorad Dragic

**Affiliations:** 1 Center for Translational Neuroscience, Faculty of Biology, University of Belgrade, Belgrade, Serbia; 2 Department of Molecular Biology and Endocrinology, VINČA Institute of Nuclear Sciences—National Institute of the Republic of Serbia, University of Belgrade, Belgrade, Serbia; 3 Center for Laser Microscopy, Faculty of Biology, University of Belgrade, Belgrade, Serbia; 4 Medical Faculty of Military Medical Academy, University of Defence, Belgrade, Serbia

**Keywords:** dendritic spines, GluN2A subunit, hippocampus, iTBS, NMDA signaling, repetitive transcranial magnetic stimulation, synaptic plasticity

## Abstract

**Background:**

Intermittent theta burst stimulation (iTBS) is increasingly explored as a non-invasive neuromodulatory approach capable of inducing long-lasting plasticity with potential therapeutic value in age-related neurological and psychiatric conditions. However, the cellular and molecular mechanisms underlying iTBS protocols remain largely unknown, limiting its further therapeutic development.

**Methods:**

Here, we investigated the behavioral, structural, synaptic, and calcium-dependent effects of a 7-day iTBS600 protocol using a combination of *in vivo*, *ex vivo*, and *in vitro* approaches. 2.5-months old male Wistar rats and *Grin2A* knockout mice were used.

**Results:**

Prolonged iTBS did not alter general locomotor activity, anxiety-like behavior, or short-term recognition memory, indicating preserved baseline behavioral function. Despite the absence of behavioral changes, prolonged iTBS induced robust structural plasticity in hippocampal CA1 neurons, increasing total spine density and selectively enhancing the proportion of thin, learning spines. Synaptosomal analysis revealed upregulation of GluN1 and GluN2A, elevated BDNF levels, and activation of downstream Akt, ERK1/2, and mTOR pathways. Prolonged iTBS also enhanced perineuronal net formation around PV^+^ interneurons across hippocampal subfields. *In vitro* recordings demonstrated increased spontaneous and evoked Ca^2+^ activity following both acute and prolonged stimulation, with the prolonged protocol uniquely extending the duration of K^+^-evoked Ca^2+^ responses. Pharmacological blockade with D-AP5 and experiments in *Grin2a*-knockout neurons revealed that these effects are dependent on NMDA receptors, particularly the GluN2A subunit.

**Conclusion:**

Together, these findings indicate that prolonged iTBS drives coordinated structural, synaptic, and Ca^2+^-dependent plasticity in the hippocampus through GluN2A- and BDNF-dependent mechanisms. This work provides mechanistic insight into how iTBS may induce sustained circuit-level adaptations relevant for therapeutic applications.

## 1 Introduction

Transcranial magnetic stimulation (TMS) is a safe, non-invasive technique that enables precise modulation of brain activity, provides valuable insights into neuronal function and offers therapeutic potential for various neurological and psychiatric disorders (Knotkova et al., 2021). TMS operates by passing a high-intensity current through a coil. According to Faraday’s law of electromagnetic induction, this generates a magnetic field that penetrates the skull and induces electrical currents in the brain area under the coil, thus altering excitability of neurons located nearby (Somaa et al., 2022; Valero-Cabré et al., 2017). Among different TMS protocols, repetitive TMS (rTMS) may induce lasting neuroplasticity after-effects, highlighting its potential for therapeutic use (Klomjai et al., 2015). Over the past four decades, since the introduction of the first rTMS device, more than 25,000 clinical and preclinical studies have been published. This compelling evidence has supported the integration of rTMS into clinical practice for the treatment of unipolar and bipolar depression, smoking cessation, obsessive-compulsive disorder, and, more recently, for reducing anxiety symptoms in patients with unipolar depression and comorbid anxiety (Benster et al., 2023; Cotovio et al., 2023). In addition to advances in device technology and performance, a significant milestone in stimulation protocols was achieved in 2007 with the introduction of the theta burst stimulation (TBS) paradigm, a specific type of rTMS protocol which enables a substantially shorter treatment duration (Huang et al., 2005; Kishi et al., 2024) with the same efficiency as the classic 10 Hz rTMS in the treatment of unipolar depression (Liu et al., 2024). Although rTMS has been extensively investigated across a broad spectrum of psychiatric, neurodevelopmental, and neurological conditions (Somaa et al., 2022), clinical consensus and regulatory approvals have thus far been achieved only for the aforementioned number of indications. Moreover, despite the growing body of research, literature still features conflicting results, varied quality of studies and lacks a consensus on optimal rTMS parameters (Dalhuisen et al., 2022; Magnuson et al., 2023; Pabst et al., 2022). The limited progress in clinical translation of rTMS can, in part, be ascribed to its deviation from the traditional bench-to-bedside translational process. Rather than progressing through the standard translational stages, the non-invasiveness and safety of rTMS facilitated a reversed translational trajectory, whereby clinical application preceded thorough mechanistic understanding. Accordingly, there is a real need to investigate rTMS in experimental animals to reveal mechanistic insights and determine when specific protocols may be effective, based on measurable and reproducible outcomes.

The basis of rTMS-induced alterations in neuronal excitability, which occur both during and after stimulation protocol, is thought to lie in the modifications of ionic balance around stimulated neurons, ultimately leading to synaptic plasticity changes. The mechanisms most often described as those underlying neuromodulatory rTMS effects are long-term potentiation (LTP) and long-term depression (LTD) (Chervyakov et al., 2015; Cirillo et al., 2017). Accordingly, the literature has pointed out that the expression of several molecular players changes after rTMS treatment, including different receptors [i.e., N-methyl-D-aspartate receptors (NMDARs) and alpha-amino-3-hydroxy-5-methyl-4-isoxazole propionic acid receptors (AMPARs)], transporters and neurotrophic factors (such as brain-derived neurotrophic factor, BDNF) known for their roles in synaptic plasticity. Importantly, due to their distinct kinetics and biophysical properties, specific subunit composition of NMDARs and AMPARs may play a significant role in the way rTMS exerts its effects (Diering and Huganir, 2018; Natale et al., 2021; Paoletti et al., 2013; Zeljkovic Jovanovic et al., 2023). In addition to functional changes, recent research has been pointed at structural alterations, leading to discovery that rTMS can induce morphological modifications of axons and dendrites, especially at the level of dendritic spines, that can persist after the stimulation period (Cambiaghi et al., 2021; Natale et al., 2021; Vlachos et al., 2012). Although substantial progress has been made in elucidating the mechanisms of rTMS, much of the existing literature remains focused on treatment outcomes in specific pathologies. Mechanistic data, especially concerning molecular pathways and specific mediators underlying both immediate and after-effects of rTMS, are still fragmentary and not yet fully investigated.

In the present study, we conducted a multi-level investigation of the effects of intermittent theta-burst stimulation (iTBS600), a shorter and equally effective protocol with clearly defined and reproducible parameters, on the hippocampus of healthy Wistar rats, with the primary goal of investigating responses in healthy brain without the confounding influence of preexisting pathology. Due to its intrinsic characteristics, the hippocampus is inherently more susceptible to plastic changes in both physiological and pathological environments, which is why many researchers utilize it in studying synaptic plasticity (Li et al., 2015; Ruggiero et al., 2021; Weerasinghe-Mudiyanselage et al., 2022). Hence, hippocampus emerges as a suitable target for investigating iTBS-induced neuroplasticity mechanisms. Additionally, data obtained from healthy animals serve as a framework for future translational research using disease models. In line with this, our objectives were to comprehensively characterize the nature, magnitude, and temporal dynamics of iTBS-induced changes across behavioral, structural, functional, and neurochemical domains of hippocampus, thereby providing a robust foundation for interpreting the biological underpinnings and therapeutic potential of a 1-week iTBS600 protocol.

## 2 Material and methods

### 2.1 Experimental design

A total of 70 *Wistar* animals (*n* = 70) were included in the *in vivo* study, divided into 22 experimental units (cages with 3–4 animals) and randomly assigned to either the Sham (*n* = 31) or iTBS (*n* = 39) experimental group before the start of the experiments. The entire *in vivo* study was divided into four experiments (EXP1-4). Animals in EXP1 were used for behavioral analysis (*n* = 10 per group), in EXP2 for histological analysis (*n* = 6 per group) and for isolation of pure synaptosomes for immunoblot and biochemical studies (*n* = 6 per group), and in EXP3 for repeated behavioral analysis (*n* = 10 per group). For *in vitro* experiments, primary hippocampal cultures were prepared from a total of 13 C57BL/6 mouse pups, randomly assigned to one of three experimental groups: Sham (*n* = 5), Acute stimulation (*n* = 3), or Prolonged stimulation (*n* = 5). Additionally, hippocampal cultures were generated from *Grin2a* KO mice (*Grin2a^+/–^*; *n* = 5), carrying a null mutation in the gene encoding the GluN2A subunit of the NMDA receptor (The Jackson Laboratory, United States). Animals in EXP4 were used for isolation of pure synaptosomes for immunoblot analysis in order to examine the after-effects of iTBS at 7 days (*n* = 3; 7 dps) and 14 days (*n* = 3; 14 dps) following the last stimulation.

### 2.2 Animals

The study was conducted on adult male Wistar rats (Crl:WI; strain code 003, Charles River Laboratories), aged 2.5 months and weighing 250–350 g, and on neonatal C57BL/6 mouse pups (C57BL/6J; Jackson Laboratories, wild-type and *Grin2a* knock-outs) at postnatal day 0–1 (P0–P1). GluN2A knockout (KO) mice with null mutation for the GluN2A-coding gene *Grin2a* were purchased from Jackson Laboratories and used to establish a colony of *Grin2a* heterozygous (HET) mice. Experimental animals were then generated from HET × HET mating in our laboratory. Mouse genomic DNA from ear punches was used for genotyping *Grin2a* knockouts and wild-types as previously described (Ogiue-Ikeda et al., 2003). The animals were obtained from the animal facility of the VINČA Institute of Nuclear Science—National Institute of Republic of Serbia, University of Belgrade. The animals were kept on a 12-h light-dark cycle at a constant ambient temperature (22 ± 2°C) and had free access to food and water.

### 2.3 Intermittent theta burst stimulation protocol

Five to seven days before the treatment, the animals were handled by the experimenter for 5–7 min in the room where the stimulation was performed in order to familiarize them with experimental conditions. Stimulation was performed with the M-100 Ultimate Stimulator with liquid cooling (Shenzhen Yingchi Technology Co., Ltd., China) using a figure-of-eight coil (model BY-45, inner diameter 25 mm). The iTBS protocol consisted of twenty trains of ten bursts each (three pulses at 50 Hz), repeated at 5 Hz (8 s interval between trains), leading to a total duration of 192 s (resulting in 600 pulses; iTBS600), with the stimulation intensity set to 35% of maximum stimulator output (MSO), generating a peak magnetic field of 735 mT. This MSO was selected because it corresponds to a level close to the motor threshold and has been shown to be sufficient to induce LTP in the healthy rat hippocampus (Ogiue-Ikeda et al., 2003). The animals in the iTBS group were gently held during stimulation, but allowed unrestricted movement during the 8 s intervals between trains (Supplementary Video 1). The rationale for selecting the iTBS600 protocol is based on its widespread clinical use, as this number of pulses is most commonly administered to patients undergoing treatment for major depressive disorder (Ørbo et al., 2023). Animals were exposed to two stimulation sessions per day for seven consecutive days, referred to hereafter as the prolonged iTBS600, with a 45–60 min interval between sessions. In the Sham group, animals were exposed to the sound artifact (63.6 ± 4.3 dB) (Escabi et al., 2019), handling and mild contact pressure by placing an object on the head, mimicking the positioning of the coil throughout the stimulation sessions. For the stimulation of primary hippocampal cultures, the iTBS600 protocol was applied with the coil positioned 3 cm above the bottom of the petri dish. The Prolonged group began stimulation on day *in vitro* (DIV) 6, was stimulated twice per day with 30 min interval between sessions, and was used for experiments 24 h after the last stimulation (DIV12/13). Cells from the Acute group were stimulated at DIV12 (twice, with 30 min interval between sessions) and immediately used for experiments. The Sham group was kept outside the incubator next to the stimulated petri dishes but was not exposed to magnetic stimulation and was used for experiments at DIV12/13. Although direct magnetic field measurements at the cell layer were not performed, the 3 cm coil-to-dish distance was selected based on preliminary experiments confirming neuronal responses to stimulation.

### 2.4 Behavioral analyses

All behavioral analyses were carried out in an isolated room with subdued light (15 lux) to which the animals were brought at least 60 min beforehand in order to acclimate. Two researchers conducted all behavioral experiments to ensure consistency in treatment of the animals. Odors were removed by cleaning the apparatus with 70% ethanol and distilled water between each animal. All animals (EXP1 and 3; *n* = 19 animals/group) were tested before the first and 24 h after the last iTBS session. All parameters were analyzed using the ANY-MAZE Video Tracking System 7.11 (Stoetling Co., United States).

### 2.5 Open field test

Open field test (OFT) was conducted to assess the differences between and within groups in terms of general locomotor activity and anxiety-like behavior. Animals were placed in the center of a black arena (100 × 100 × 50 cm) located in an isolated room and their exploratory activity was recorded for 10 min.

### 2.6 Novel object recognition test

Novel Object Recognition test (NORT) was performed to assess short-term memory of both groups of rats. The OFT was used as a habituation phase for the NORT test. The animals were placed in the center of the arena at an equal distance from two identical, circular, yellow objects and were allowed to freely explore the arena and the objects for 10 min (sampling phase). Two hours after the sampling phase, the animals were returned to the arena, where one of the objects was replaced by a triangular red object, which they were allowed to explore freely for 10 min. Animals that showed a preference for an object during the sampling phase were excluded from further trials (*n* = 1). The time spent with the new object relative to the total time spent with both objects was expressed as a recognition index (RI) and used as the main indicator of short-term memory efficacy.

### 2.7 Brain isolation for immunohistological analyses

Twenty-four hours after the last stimulation, the animals of both groups (EXP2; *n* = 3 animals/group) were decapitated with a small guillotine (Harvard apparatus, United States) and the brains were quickly removed from the skull and fixed in 4% paraformaldehyde (PFA) for 24 h, dehydrated and cryoprotected in graded sucrose solution (10–30% in 0.2 M PBS, pH 7.4) as described previously (Stekic et al., 2025). The 25 μm-thick sagittal sections, prepared on cryostat, were mounted on superfrost glass slides, air-dried for 1–2 h at room temperature (RT) and stored at -20°C until use. After rehydration in PBS, the sections were treated with 0.3 % hydrogen peroxide for 20 min and washed with PBS for 3 × 5 min. Sections were then incubated with 0.1 % Triton X-100 for 15 min, washed in PBS for 3 × 5 min and probed for 30 min at RT with 5 % normal donkey serum (NDS) in PBS as a blocking solution, followed by incubation with primary antibody overnight at 4°C (Supplementary Table 1). The next day, slides were washed in PBS for 3 × 5 min and probed with adequate secondary antibody for 2 h at RT (Supplementary Table 1). The signal was visualized with 3,3-S-diaminobenzidine-tetrahydrochloride kit (DAB, Abcam, United Kingdom) as a chromogen for HRP-conjugated secondary antibodies. After dehydration in graded ethanol (70–100%) and clearance in xylene, the sections were mounted with the DPX-mounting medium (Sigma Aldrich, United States).

### 2.8 Staining of perineuronal nets and imaging

For staining of perineuronal nets (EXP 2; *n* = 3 animals/group, 2–4 sections per animal), non-specific antigen binding was blocked with 5% NDS and 0.2% Triton X-100 in PBS for 40 min at room temperature. Endogenous biotin was blocked using a streptavidin–biotin blocking kit (Vector Laboratories), with 15 min incubations for each solution and PBS rinses between steps. Perineuronal nets (PNNs) were labeled using *Wisteria floribunda* agglutinin (WFA; 1:100, L1516, Millipore Sigma, United States) together with anti-parvalbumin antibody (Supplementary Table 1) diluted in 1% NDS in PBS. After incubation of primary antibodies overnight at 4°C, sections were washed in PBS (5 × 5 min) and then incubated for 2 h at room temperature with appropriate secondary antibodies. Sections were washed again in PBS (5 × 5 min), coverslipped with Mowiol mounting medium, and dried overnight before imaging. Sagittal hippocampal sections were imaged using a confocal laser-scanning microscope (LSM 510, Carl Zeiss, Jena, Germany) equipped with Arg (488 nm) and HeNe (543 nm, 633 nm) lasers, and 40 × (Plan-Apochromat, N_*A*_ = 1.3) or 63 × (DIC, N_*A*_ = 1.4) oil immersion objectives. PNNs and parvalbumin-positive (PV^+^) neurons from CA1–CA3 subfields were imaged in 2D mode. All acquisition parameters were identical between two groups to allow direct comparison of pixel intensities. Images were analyzed in ImageJ (ImageJ v1.46r, NIH, United States).^1^ In each section, the mean pixel intensity of PNNs surrounding PV^+^ (WFA^+^ PV^+^) and PV^–^ (WFA^+^ PV^–^) cells, and PV signal intensity in neurons with (PV^+^ WFA^+^) or without (PV^+^ WFA^–^) PNNs, was quantified across CA1–CA3. Background was subtracted using the rolling ball method (Murthy et al., 2019), and regions of interest (ROIs) were manually drawn around each PNN and PV cell to measure mean pixel intensity (range 0–255). All imaging and image analyses were performed manually by an experimenter blind to group allocation.

### 2.9 Modified Golgi-Cox staining for visualization of dendritic spines

Modified rapid Golgi-Cox staining was performed with slight modifications according to Zaqout and Kaindl (2016) and Shan et al. (2022). In brief, brains (*n* = 3 animals/group) were quickly removed from the skull, cut into two halves midsagittally and placed in impregnating solution (IS), consisting of potassium chromate, potassium dichromate and mercury(II) chloride, for 15 days in the dark, as described in Zaqout and Kaindl (2016). The brains were then washed in distilled water to remove excess IS, dehydrated and cryoprotected in a 30% sucrose solution in 0.2 M PB for 14 days. The 100 μm-thick sagittal sections were obtained using cryostat and mounted on gelatin-coated slides, air-dried for 1–2 h and stored in the dark at RT until use. For the development step, slides were put in 1% Li_2_CO_3_ in deionized water (diH_2_O) for 25 min, then washed in diH_2_O for 5 min, dehydrated in graded ethanol (70–100%) and cleared in xylene for 20 min. Sections were mounted using DPX mounting medium (Sigma Aldrich, United States).

### 2.10 Light and confocal microscopy and image acquisition and analysis

Immunostained sections were examined using a LEITZ DM RB light microscope (Leica Mikroskopie and Systems GmbH, Wetzlar, Germany) equipped with a LEICA DFC320 CCD camera (Leica Microsystems Ltd., Heerbrugg, Switzerland). Image acquisition was performed with LEICA DFC Twain Software (Leica, Wetzlar, Germany). For the quantification of c-Fos-positive neurons in hippocampal sections, images of the CA1 and CA3 subfields and dentate gyrus (DG) of the dorsal hippocampus were captured at 20× magnification in a medio-lateral orientation. From each animal, 3–4 images per section were obtained from 3 to 4 sections, yielding a total of 45–50 images per hippocampal subfield for each experimental group. Only clearly stained c-Fos-positive neurons were counted in a high-power field (3,072 × 2,304 pixels, 1 μm = 5.1 pixels, HPF = 0.27 mm^2^) using the ImageJ *Cell counter* plugin. Golgi-Cox-stained sections of dorsal hippocampus were visualized using confocal laser-scanning microscope (LSM 510, Carl Zeiss GmbH, Germany) using HeNe (633 nm) laser. Z-stack images of Golgi-Cox-stained secondary and tertiary basal dendritic branches (1,024 × 1,024 pixels, 1 pixel = 0.09 μm, up to 20 μm total on *Z*-axis, optical section thickness = 0.5 μm, i.e., up to 40 images per stack) were captured using 100 × (DIC, N_*A*_ = 1.4) oil objective and monochrome camera AxioCam ICm1 camera (Carl Zeiss GmbH, Germany). Analysis and classification of spine morphology was assessed using ImageJ and Reconstruct software,^2^ according to Risher et al. (2014). Five neurons per animal were analyzed, with three basal dendritic branches assessed per neuron in the CA1 subfield, yielding a total of 2,345 dendritic spines from both experimental groups. Spine classification followed the criteria described in Zaqout and Kaindl (2016), with the modification that mushroom spines were identified by a head width > 0.5 μm (Jedynak et al., 2007).

### 2.11 Isolation of purified hippocampal synaptosomes and Western blot analysis

Twenty-four hours after the last stimulation, animals of both groups (EXP2; *n* = 5–6 animals/group) were decapitated and the brains were rinsed in ice-cold saline. Hippocampi were carefully dissected on ice and purified synaptosomes were isolated using Syn-PER Synaptic Protein Extraction Reagent (Thermo Fisher, United States), resulting in both functional, purified synaptosome fraction (Supplementary Figure 4) and cytosolic fraction according to manufacturer instructions. The protein quantity in each sample was determined using the Pierce BCA Protein Assay Kit (Thermo Fisher Scientific, United States). Equal sample aliquots (15 μg of the sample proteins) were resolved using SDS-PAGE and transferred to PVDF membrane using semidry Trans-Blot Turbo Transfer System (Bio-Rad, United States). The supporting membrane was blocked in 5% non-fat milk or 5% bovine serum albumin for 45 min, washed in TBST and then incubated with primary antibodies (Supplementary Table 2), after which it was rinsed in TBST and incubated with appropriate HRP-conjugated secondary antibodies. Primary and secondary antibodies were removed by stripping membranes in mild stripping buffer pH 2.2 (0.2 mmol/L glycine, 0.1% SDS, and 1% Tween-20) to blot other proteins. Chemiluminescent signals were detected in ChemiDoc Imaging Systems (Bio-Rad, United States) using ECL solution (Bio-Rad, United States). The optical densities (OD) of the target band and GAPDH band (loading control) in each lane were determined in the ImageJ program and the ratio in each lane was expressed relative to Sham.

### 2.12 Isolation of primary hippocampal neurons

Primary hippocampal neurons were isolated from newborn C57BL/6 mouse pups (P0.5). Briefly, hippocampi were dissected from cortical tissue in Dulbecco’s Modified Eagle’s Medium (DMEM, D5648, Sigma-Aldrich) supplemented with 0.1 mg/mL streptomycin (Gibco, ThermoFisher Scientific) and 100 IJ/mL penicillin (Gibco, ThermoFisher Scientific). The tissue was enzymatically digested with 1 mg/mL trypsin at 37°C for 10 min, followed by three washes with BrainPhys Neuronal Medium (05794, StemCell Technologies) containing 2% fetal bovine serum (FBS, Gibco, ThermoFisher Scientific). The loosened tissue was then mechanically dissociated in 200 μL of the same medium with 200 μL pipette tip. The cell suspension was transferred to a 15 mL tube, diluted with 900 μL medium, and centrifuged at 400 g for 4 min, at RT. The supernatant was discarded, the pellet was resuspended first in 200 μL of medium, and then in additional 900 μL of medium, for a second centrifugation. Finally, the pellet was resuspended in a Plating medium consisting of NeuroCult Neuronal Plating Medium (05794, StemCell Technologies) supplemented with 2% NeuroCult SM1 Neuronal Supplement (05794, StemCell Technologies), 1% GlutaMAX (Gibco, ThermoFisher Scientific), 25 μM glutamic acid, and 10 μg/mL gentamicin. Cells were plated on pre-coated 10 mm glass coverslips (coated with 50 μg/mL poly-L-ornithine (PLO), and 10μg/mL laminin), placed in 24 well plates (40,000 cells/coverslip) for calcium imaging and electrophysiological recordings. Initially, 40 μL of cell suspension was added to each coverslip, followed by 400 μL of Plating medium after 4 h. For immunoblot analysis 500,000 cells were seeded in 35 mm Petri dishes (precoated with 50μg/mL poly-L-ornithine). Starting on DIV5, half of the culture medium was replaced with Maturation medium consisting of BrainPhys Neuronal Medium supplemented with 2% SM1, 1% GlutaMAX, and 10 μg/mL gentamicin, and the medium exchange was repeated every 3 days thereafter. Cells were maintained in a humidified incubator at 37°C with 5% CO*2* to ensure optimal physiological conditions and pH stability of the culture medium.

### 2.13 Calcium imaging

Intracellular Ca^2+^ dynamics was assessed using the fluorescent indicator Fluo-4 AM (Molecular Probes, United States). A total of 40,000 cells were plated onto 10 mm glass coverslips pre-coated with PLO and laminin, and imaged at DIV12/13. Prior to imaging, cells were incubated in 5 μM Fluo-4 AM solution prepared in extracellular solution (ECS, 140 mM NaCl, 5 mM KCl, 2 mM CaCl_2_, 1 mM MgCl_2_, 10 mM HEPES, and 10 mM glucose, pH adjusted to 7.4, with osmolarity of 320–340 mOsm) for 30 min, followed by a 10 min wash in dye-free ECS in the dark. Fluorescence imaging was performed using an AxioObserver A1 inverted microscope equipped with an LD LCI Plan-Apochromat 25 × /0.8 NA water-immersion objective (Carl Zeiss GmbH, Germany). Images were acquired at 1 Hz using an EM512 CCD camera (Evolve, Photometrics, United States) controlled by VisiView software (Visitron Systems GmbH, Germany). Fluo-4 was excited at 480 nm using a xenon lamp (Ushio, Japan) integrated with a VisiChrome polychromatic illumination system, and emission was collected through an FITC filter set (ex: HQ480/40x, em: HQ535/50m, dichroic Q505LP, Chroma Technology Inc., United States). Cells were continuously perfused with ECS at 4 mL/min using a custom-built gravity-driven system. Glass pipettes (0.8 mm inner diameter) were positioned at a 45° angle, approximately 350 μm laterally and 1 mm above the imaging field. Solution exchange was controlled by a high-speed valve system (VC3, ALA Scientific Instruments, United States), and chamber volume (∼1 mL) was maintained by continuous suction. Baseline fluorescence was recorded from 0 to 300 s. At 300 s, cells were stimulated either with high-potassium ECS (50 mM K^+^) to induce depolarization or with glutamate (25 μM). The high-K^+^ ECS was composed of 85 mM NaCl, 50 mM KCl, 2 mM CaCl_2_, 1 mM MgCl_2_, 10 mM HEPES, and 10 mM glucose (pH 7.4, 320–340 mOsm/L). Spontaneous Ca^2+^ fluctuations were monitored during the baseline period (0–300 s). In experiments using glutamate-induced depolarization, cells were treated with 30 μM D-(-)-2-amino-5-phosphonopentanoic acid (D-AP5) 10 min prior to the first stimulation session. This concentration was selected based on previous reports demonstrating its non-toxic effects (Martin et al., 2003) and, given the 7-day treatment duration, was chosen as a lower alternative to the commonly used 50 μM concentration (Vlachos et al., 2012). Fluorescence intensity was quantified by calculating the mean pixel intensity within user-defined regions of interest (ROIs) corresponding to individual cells using ImageJ. Fluorescence values (F) were normalized to baseline intensity (F_0_) using a custom MATLAB script.

### 2.14 Electrophysiological recordings

Whole-cell patch clamp recordings were performed on hippocampal primary neurons at DIV12/13 in ECS. Cells were visualized with an inverted Zeiss Axiovert 10 microscope (Carl Zeiss GmbH, Germany) using Plan-Neofluar 20 × /0.5 NA (Carl Zeiss GmbH, Germany), equipped with DCC1545M—USB 2.0 CMOS Camera, 1,280 ×1,024, Monochrome Sensor (Thorlabs, United States). Patch pipettes were pulled from borosilicate glass with filament (GB150F-10, 0.86 × 1.50 × 100 mm, Science Products GmbH, Germany) with P-97 Flaming/Brown Micropipette Puller (Sutter Instruments, United States). Pipette resistance, after filling with internal solution (in mM: 123 potassium gluconate, 10 HEPES, 4 MgCl_2_, 0.1 CaCl_2_, 4 ATP-tris, 0.3 GTP-tris, 1 EGTA and 10 phosphocreatine di(tris); pH 7.2 adjusted with KOH, ∼295 mOsm/L) was 4–7 MΩ. All experiments were carried out at room temperature (22–25°C). After achieving the whole cell configuration, capacitive currents were compensated, and series resistance ranged from 10 to 30 MΩ. Whole-cell patch-clamp recordings were performed in current-clamp mode. Electrical signals were amplified using an Axopatch 200B amplifier (Molecular Devices, United States) and digitized with a Digidata 1550B (Molecular Devices, United States). Data acquisition was carried out with Clampex 11.2 software. Only neurons with resting membrane potential between -60 and -40 mV were chosen for recordings. Intrinsic neuronal physiology and action potential firing were assessed using a series of current injections ranging from –100 pA to +280 pA in 20 pA increments (400 ms duration). The sag potential was quantified in response to a –100 pA current step, while input resistance was determined using hyperpolarizing steps of –60, –40, and –20 pA. Individual action potentials (APs) analyzed for spike parameters (threshold, amplitude, half-width, afterhyperpolarization, etc.) corresponded to the first AP evoked at rheobase current injection in current-clamp mode. For AP threshold determination, voltage traces were first low-pass filtered, after which the first derivative of the filtered signal (dV/dt) was calculated. The AP threshold was defined as the membrane potential at which dV/dt reached 1 mV/ms and was obtained by linear interpolation between the two adjacent time points at which this criterion was met.

The resting membrane potential was calculated as the average of the voltage traces in the current clamp mode, before the injection of any current.

### 2.15 Statistical analysis

The required number of animals per group was determined using G*Power software (version 3.1) based on an *a priori* power analysis for a two-tailed Student’s *t*-test or two-way ANOVA, assuming a large effect size (*d* = 1.1–1.3), an alpha level of 0.05, and a statistical power of 0.80. These parameters were derived from preliminary pilot data and relevant published studies (Stekic et al., 2022; Zeljkovic Jovanovic et al., 2023). All datasets were assessed for normality using the Shapiro–Wilk test. Depending on the distribution, either parametric or nonparametric statistical tests were applied as appropriate. Behavioral test results were analyzed using repeated-measures two-way ANOVA (*mixed-design*), with Šidák’s *post*-*hoc* test employed for multiple comparisons. Histological, morphometric, immunoblotting, and electrophysiological data were analyzed using two-tailed Student’s *t*-test applying Welch’s correction when variances were unequal. Calcium imaging data were analyzed using one-way ANOVA or two-way ANOVA (*mixed-design*) followed by Dunnet’s or Tuckey’s *post-hoc* test for multiple comparisons, respectively. Wild-type and knockout calcium imaging data were analyzed using two-tailed Student’s *t*-test. Immunoblot analyses of temporal changes across three time points were performed using the Kruskal–Wallis test, followed by Dunn’s *post-hoc* test for multiple comparisons. Correlations were tested using Pearson’s correlation test. To minimize bias and improve study rigor, experimental units were randomly assigned to groups with equal and predetermined sample sizes sufficient to ensure statistical power. Performance bias was reduced through standardized procedures and constant experimental conditions. Values are presented as mean ± SD when biological replicates were used, and as mean ± SEM when multiple measurements were obtained from a single animal. A significance level of *p* < 0.05 was considered statistically significant. Data analysis and graphical representation were performed using the GraphPad Prism 9.0 software package (San Diego, United States).

## 3 Results

### 3.1 Prolonged iTBS does not alter general locomotor activity or induce anxiety-like behavior

**FIGURE 1:**
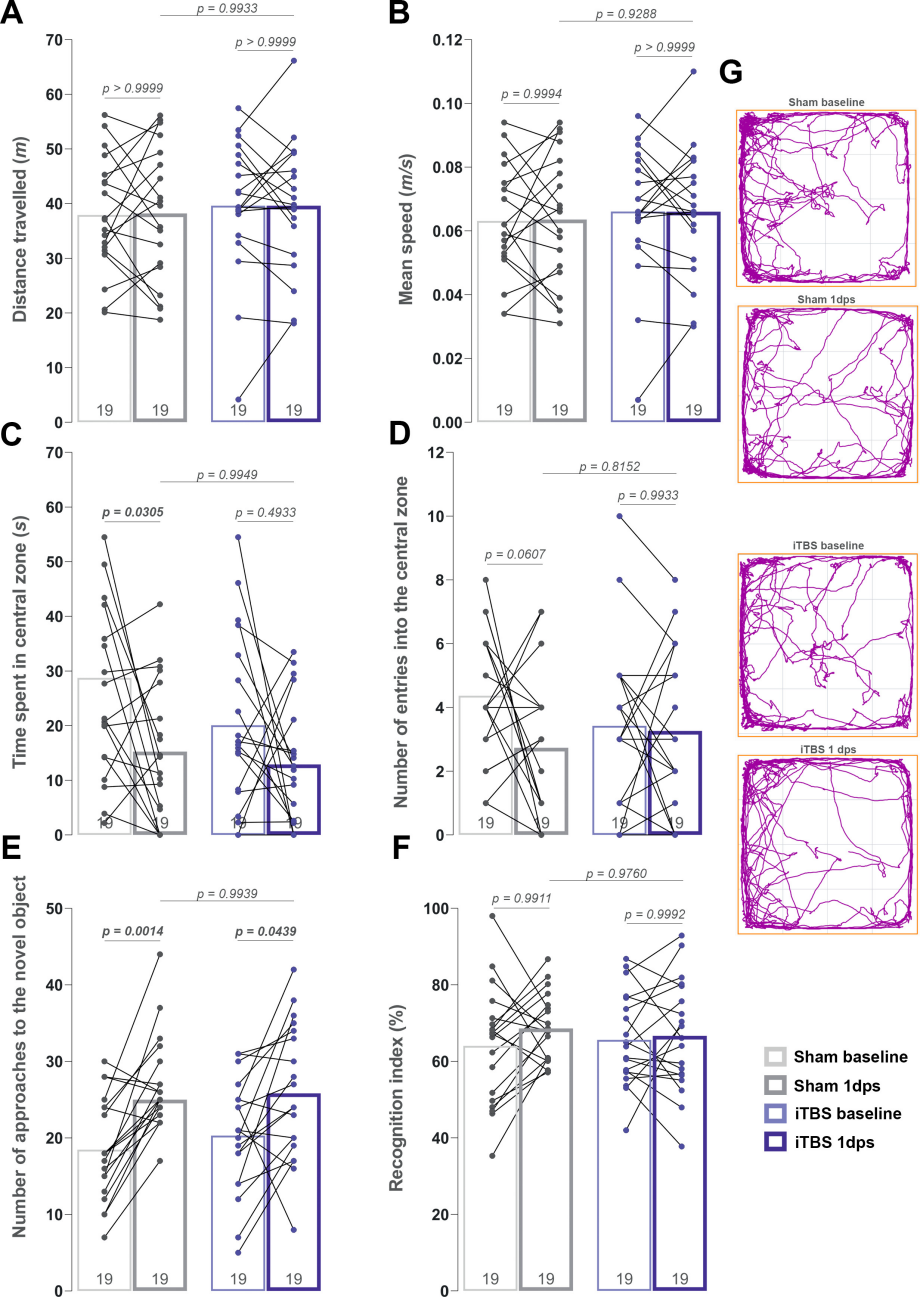
Behavioral measures obtained in the open field test (OFT) and novel object recognition test (NORT). Both tests were performed 1 day before the first iTBS session (baseline) and 1 day after the last iTBS session (1 day post-stimulation, 1 dps). **(A–D)** Parameters investigated in the OFT: **(A)** total distance traveled, **(B)** mean speed, **(C)** time spent in the central zone and **(D)** number of entries into the central zone. **(G)** Representative track plots for both groups before and after stimulation. **(E,F)** Parameters investigated in the NORT: **(E)** number of approaches to the novel object and **(F)** time spent investigating the novel object expressed as recognition index. Dots in the graphs represent the values of individual animals, with lines connecting each value before and after stimulation for respective animals. Numbers at the bottom of the graphs show the number of animals included in the analysis (*n* = 19 animals per group). Obtained results were analyzed using repeated-measures two-way ANOVA (mixed-design), with Šidák’s *post-hoc* test employed for multiple comparisons. Data are expressed as mean ± SD. Significance is shown on graphs as *p*-value, in bold for *p* < 0.05.

Throughout the experimental period, general health, body weight, and in-cage behavior of animals were monitored. All animals maintained normal general health, as indicated by regular grooming behavior (clean fur, eyes, and nails), strong tail tonus, and normal mobility. Social interactions within experimental units remained typical, with no obvious signs of aggression or anxiety-like behavior. As expected, all animals exhibited normal weight gain (Supplementary Figure 1), with no significant differences observed between groups, indicating that the stimulation protocol did not induce visible stress or interfere with feeding behavior. One day before and after the stimulation period, OFT was used to evaluate general locomotor activity and anxiety-like behavior. No significant main effects of treatment, time, or their interaction were observed for either distance traveled [Figure 1A, Time: *F*_(1, 18)_ = 0.007, *p* = 0.930, Treatment: *F*_(1, 18)_ = 0.436, *p* = 0.533, Interaction: *F*_(1, 18)_ = 0.008, *p* = 0.933] or mean speed [Figure 1B, Time: *F*_(1, 18)_ = 0.007, *p* = 0.931, Treatment: *F*_(1, 18)_ = 0.403, *p* = 0.517, Interaction: *F*_(1, 18)_ = 0.007, *p* = 0.926], indicating unchanged general locomotor activity. To assess anxiety-like behavior, the number of entries into, and the time spent in, the central zone of the OFT were quantified. For the time spent in the central zone, only the effect of time was observed [Figure 1C, Time: *F*_(1, 18)_ = 6.121, *p* = 0.023, Treatment: *F*_(1, 18)_ = 4.057, *p* = 0.059, Interaction: *F*_(1, 18)_ = 1.106, *p* = 0.306], while no effects were detected for number of entries in the central zone [Figure 1D, Time: *F*_(1, 18)_ = 2.315, *p* = 0.145, Treatment: *F*_(1, 18)_ = 0.179, *p* = 0.677, Interaction: *F*_(1, 18)_ = 3.035, *p* = 0.098]. To further investigate the behavioral patterns related to anxiety-like behavior, the number of entries into the central zone was correlated with the time spent there for each group before and after the stimulation period. In the Sham group, a moderate and significant positive correlation was observed before treatment (Supplementary Figure 2A, *r* = 0.645, *p* = 0.044), which was further strengthened after treatment (Supplementary Figure 2B, *r* = 0.795, *p* < 0.0001). Similarly, the iTBS group showed a stable and significant positive correlation both before (Supplementary Figure 2C, *r* = 0.608, *p* = 0.0059) and after stimulation (Supplementary Figure 2D, *r* = 0.586, *p* = 0.0089), suggesting that the stimulation protocol did not disrupt the relationship between these exploratory parameters. Overall, these results suggest that behavioral patterns and coherence were maintained in both groups, despite a reduction in total entries, and that iTBS600 did not influence baseline anxiety-like behavior.

### 3.2 Prolonged iTBS does not affect short-term learning and memory

To assess the effects of iTBS on short-term learning and memory, NORT was conducted 1 day before and 1 day following the 7-day stimulation period (1-day post-stimulation, 1 dps). Given that NORT is based on spontaneous exploration, the number of approaches to the novel object and the recognition index (RI) were quantified as primary outcome measures. For the number of approaches to the novel object, only the effect of time was observed, being significantly increased in both groups after stimulation [Figure 1E, Time: *F*_(1, 18)_ = 28.20, *p* < 0.0001, Treatment: *F*_(1, 18)_ = 0.037, *p* = 0.848, Interaction: *F*_(1, 18)_ = 1.246, *p* = 0.279], while no effects were detected for RI [Figure 1F, Time: *F*_(1, 18)_ = 1.099, *p* = 0.308, Treatment: *F*_(1, 18)_ = 0.002, *p* = 0.958, Interaction: *F*_(1, 18)_ = 0.934, *p* = 0.346]. Taken together with OFT results, these findings suggest that exploratory activity remained unaltered in both groups, and that prolonged iTBS did not affect recognition memory, as indicated by the unchanged RI.

### 3.3 Prolonged iTBS promotes dendritic structural plasticity in CA1 pyramidal neurons

**FIGURE 2:**
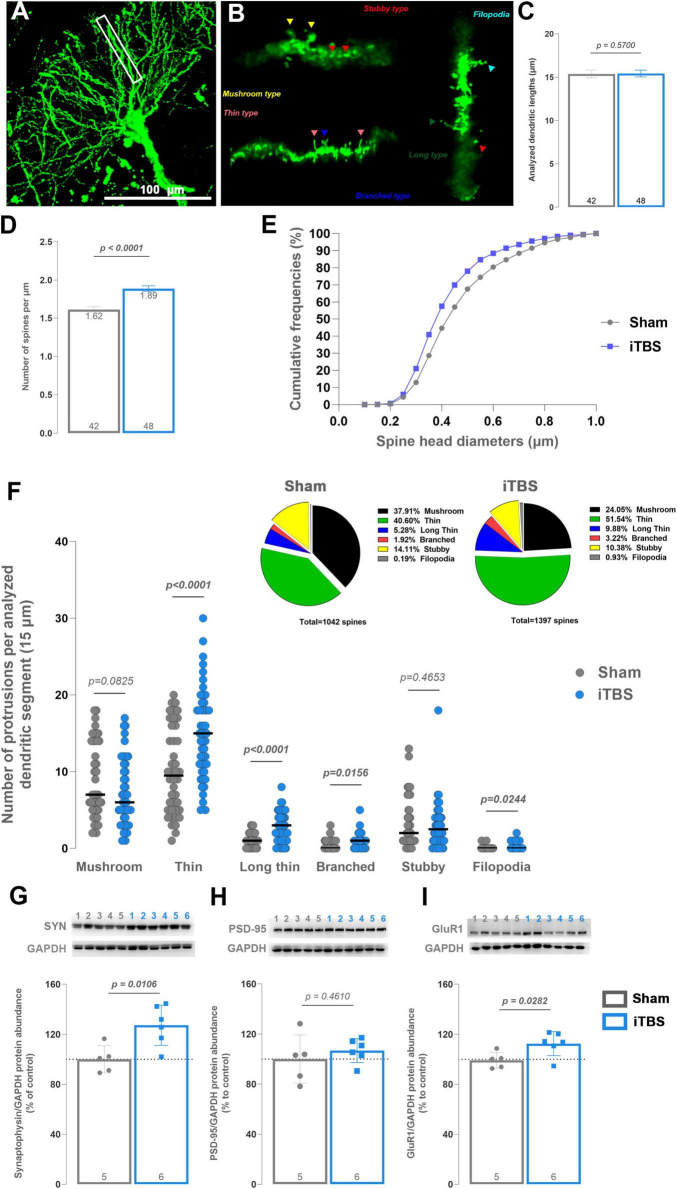
Golgi-Cox (GC) staining and morphological analysis of dendritic spines on secondary and tertiary basal dendrites of pyramidal neurons in CA1 subfield of dorsal hippocampus and western blot analysis of main synaptic protein markers in hippocampal synaptosomal fraction. Fluorescent micrographs show representative pyramidal neuron **(A)** with a single basal dendritic branch bordered in a white rectangle, **(B)** enlarged and marked for different dendritic spine types using colored triangles. **(C)** The length of the analyzed dendritic branches was around 15 μm for all branches, with their number given at the bottom of the graph for each group, providing evidence that the observed differences in dendritic spine number do not stem from dissimilar branch lengths. **(D)** The number of dendritic spines is presented as a number per μm, i.e., spine density, with the number of analyzed branches indicated at the bottom, and the spine density given at the top of the graph for each group. **(E,F)** Cumulative frequencies of spine head diameters and distribution of spine types, obtained from analysis of spine length and width. The pie charts indicate percentual proportion of each spine type in relation to the total number of spines in each group, as shown below the pie charts. Obtained results were analyzed using two-tailed Student’s *t*-test. Data are expressed as mean ± SEM. *n* = 3 animals per group **(G–I)** Western blot analysis of protein markers of **(G)** presynapse (synaptophysin), **(H)** postsynapse (PSD-95) and **(I)** indicators of synaptic maturation (GluR1) in synaptosomal fraction. Protein expression is shown as the percentage of control. Representative membranes are shown above graphs for each protein and GAPDH for that membrane, with numbers above indicating the sample layout. Dots in the graphs represent the values of individual animals. Numbers at the bottom of the graphs show the number of animals included in the analysis (*n* = 5–6 animals per group). Obtained results were analyzed using two-tailed Student’s *t*-test. Data are expressed as arbitrary units derived from optical density ± SD. Significance is shown on graphs as *p*-value, in bold for *p* < 0.05.

To identify hippocampal subfields responsive to prolonged iTBS600, c-Fos immunostaining of the hippocampus was performed (Supplementary Figure 3). Among hippocampal subfields, only the CA1 subfield showed a statistically significant increase in the number of c-Fos–positive neurons after stimulation, indicating neuronal activation and relevance for downstream structural plasticity analysis (Supplementary Figures 3A,B). Therefore, the CA1 subfield (Figure 2A) was selected for the subsequent investigation of dendritic spines (Figure 2B). Ensuring that the length of quantified dendritic branches wasn’t a confounding factor (Figure 2C, *t* = 0.5701, d_*f*_ = 88, *p* = 0.5700), analysis revealed that prolonged iTBS600 led to a statistically significant increase (∼17%) in the total number of dendritic spines in hippocampal CA1 pyramidal neurons compared to Sham (Figure 2D, *t* = 5.148, d_*f*_ = 88, *p* < 0.0001). In addition to increased spine density, morphological parameters such as spine length and diameter are commonly used as indicators of synaptic maturation and stability. Accordingly, the cumulative frequency of spine head diameters showed a shift to the left for the iTBS600 curve (Figure 2E), indicating that stimulation increased the proportion of thin spines. Consequently, a type-specific analysis was conducted (Figure 2F), revealing that iTBS increased the proportion of thin (*t* = 4.097, d_*f*_ = 88, *p* < 0.0001), long thin (*t* = 4.840, d_*f*_ = 88, *p* < 0.0001), branched spines (*t* = 2.468, d_*f*_ = 88, *p* = 0.0156), and filopodia (*t* = 2.290, d_*f*_ = 88, *p* = 0.0244), concomitant with slight decrease in the proportion of mushroom-type spines (*t* = 1.757, d_*f*_ = 88, *p* = 0.0825). Next, purified synaptosomes were probed for markers of presynaptic and postsynaptic structures, as well as for indicators of synaptic maturation. Expression of synaptophysin (Figure 2G, *t* = 3.214, d_*f*_ = 9, *p* = 0.0106) and GluR1 (Figure 2I, *t* = 2.612, d_*f*_ = 9, *p* = 0.0282) was significantly increased following iTBS, whereas PSD-95 (Figure 2H, *t* = 0.7701, d_*f*_ = 9, *p* = 0.461) remained unaltered.

### 3.4 Prolonged iTBS reshapes glutamatergic signaling: GluN1/GluN2A receptor upregulation and transporter-mediated compensation in purified hippocampal synaptosomes

**FIGURE 3:**
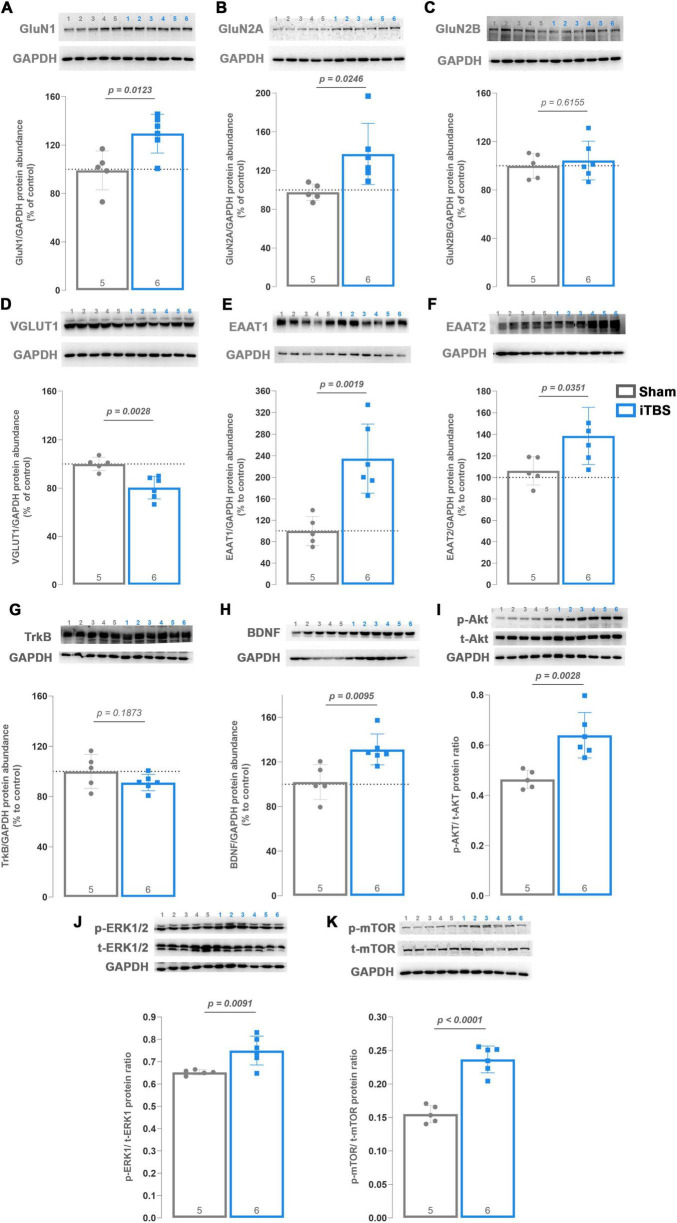
Western blot analysis of components of glutamate signalization and BDNF-TrkB and downstream signalization in hippocampal synaptosomal fraction. Graphs and representative membranes of **(A)** GluN1, **(B)** GluN2A, **(C)** GluN2B, **(D)** VGLUT1, **(E)** EAAT1, **(F)** EAAT2, **(G)** TrkB, **(H)** BDNF, **(I)** phospho/total-Akt, **(J)** phospho/total-ERK1/2 and **(K)** phospho/total-mTOR, with their respective GAPDH. Numbers above the membranes show the sample layout. Protein expression is presented as the percentage of control, except for the p/t-Akt, p/t-ERK1/2 and p/t-mTOR. Dots in the graphs represent the values of individual animals. Numbers at the bottom of the graphs show the number of animals included in the analysis (*n* = 5**–**6 animals per group). Obtained results were analyzed using two-tailed Student’s *t*-test. Data are expressed as arbitrary units derived from optical density ± SD. Significance is shown on graphs as *p*-value, in bold for *p* < 0.05.

To further evaluate the impact of prolonged iTBS on the expression of key components of glutamatergic signaling, immunoblot analysis was performed on purified synaptosomes (Figure 3). Following prolonged iTBS600, increased expression of GluN1 (Figure 3A, *t* = 3.12, d_*f*_ = 9, *p* = 0.0123) and GluN2A (Figure 3B, *t* = 2.696, d_*f*_ = 9, *p* = 0.0246) was observed compared to the Sham group, while no changes were detected in GluN2B levels (Figure 3C, *t* = 0.52, d_*f*_ = 9, *p* = 0.6155). Interestingly, a decrease in VGLUT1 (Figure 3D, *t* = 4.07, d_*f*_ = 9, *p* = 0.0028) expression was observed following prolonged iTBS, whereas EAAT1 (Figure 3E, *t* = 4.34, d_*f*_ = 9, *p* = 0.0019) and EAAT2 (Figure 3F, *t* = 2.47, d_*f*_ = 9, *p* = 0.0351) levels were markedly upregulated, pointing toward increased GluN1/GluN2A-mediated signaling and glutamate turnover.

### 3.5 Prolonged iTBS activates BDNF-Akt/ERK/mTOR pathways in the purified hippocampal synaptosomes

Considering that prolonged iTBS alters glutamate-mediated signaling, changes indicative of LTP-like phenomena, downstream signaling pathways mediated by BDNF were subsequently investigated (Figure 3). While no changes in TrkB receptor (Figure 3G, *t* = 1.42, d_*f*_ = 9, *p* = 0.1873) expression were detected, an approximately 30% increase in BDNF levels was observed (Figure 3H, *t* = 3.28, d_*f*_ = 9, *p* = 0.0095). Since activation of the TrkB–BDNF pathway is known to promote spine growth and protein synthesis (Zagrebelsky et al., 2020), the expression of downstream kinases involved in this process, Akt, ERK1/2, and mTOR, was examined. As expected, an increase in the expression of phosphorylated Akt (Figure 3I, *t* = 4.07, d_*f*_ = 9, *p* = 0.0028), ERK1 (Figure 3J, *t* = 3.03, d_*f*_ = 9, *p* = 0.0091) and mTOR (Figure 3K, *t* = 7.81, d_*f*_ = 9, *p* < 0.0001) was found following prolonged iTBS.

### 3.6 Prolonged iTBS enhances perineuronal net formation around PV^+^ interneurons in the hippocampus

**FIGURE 4:**
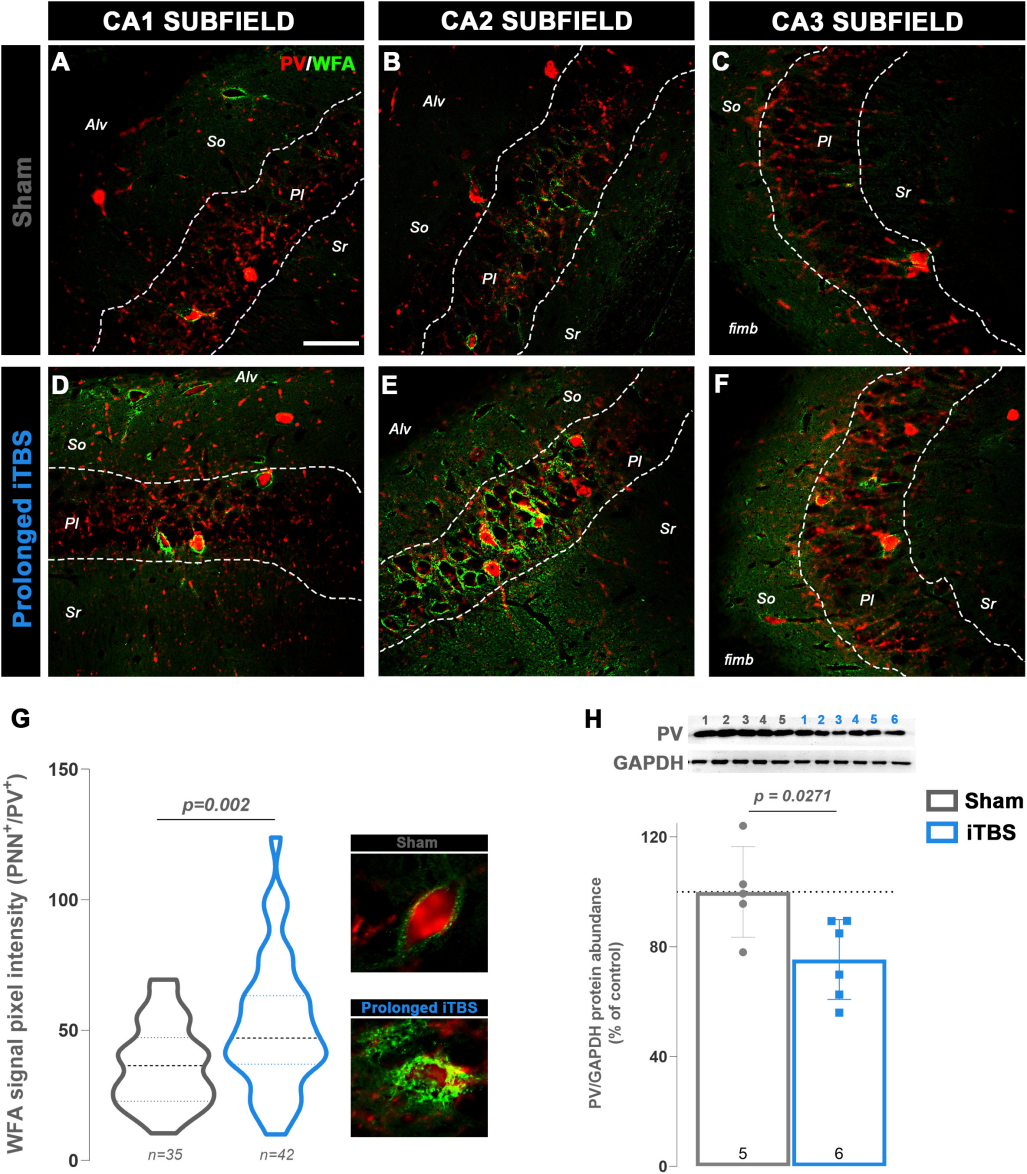
Perineuronal nets and parvalbumin-positive (PV+) neurons in hippocampus and western blot analysis of PV expression in hippocampal cytosolic fraction. Representative fluorescent micrographs showing PV+ neurons in red and perineuronal nets (marked with WFA) in green, in **(A,D)** CA1, **(B,E)** CA2 and **(C,F)** CA3 subfields of dorsal hippocampus for both experimental groups. The dotted line outlines the pyramidal layer (Pl) in all micrographs; Alv—alveus, So—stratum oriens, Sr—stratum radiatum, fimb—fimbria. **(G)** The graph showing pixel intensity for WFA signal for PNNs surrounding PV+ neurons, with representative micrographs for each group. Numbers at the bottom of the graphs show the number of cells included in the analysis (*n* = 3 animals per group). Obtained results were analyzed using two-tailed Student’s *t*-test. **(H)** Graph and representative membranes of PV with the respective GAPDH. Numbers above the membranes show the sample layout. Protein expression is presented as the percentage of control. Dots in the graphs represent the values of individual animals. Numbers at the bottom of the graphs show the number of animals included in the analysis (*n* = 5**–**6 animals per group). Obtained results were analyzed using two-tailed Student’s *t*-test. Data are expressed as arbitrary units derived from optical density ± SD. Significance is shown on graphs as *p*-value, in bold for *p* < 0.05.

Since enhanced structural and functional plasticity is often accompanied by remodeling of perineuronal nets (PNNs), particularly within the hippocampus, we examined PNNs surrounding parvalbumin-positive (PV^+^) interneurons across all three hippocampal subfields in Sham- (Figures 4A–C) and iTBS-treated groups (Figures 4D–F). Following prolonged iTBS, animals exhibited a significantly higher intensity of PNNs surrounding PV^+^ neurons across all three hippocampal subfields, with the most pronounced increase observed in CA2 (Figures 4B,E), compared to Sham (Figure 4G; *t* = 3.206, d_*f*_ = 70.14, *p* = 0.002). Interestingly, the cytosolic expression of parvalbumin (PV) was significantly reduced in the iTBS group (Figure 4H; *t* = 2.636, d_*f*_ = 9, *p* = 0.0271).

### 3.7 Both acute and prolonged iTBS induce changes in spontaneous and evoked Ca^2+^ responses

**FIGURE 5:**
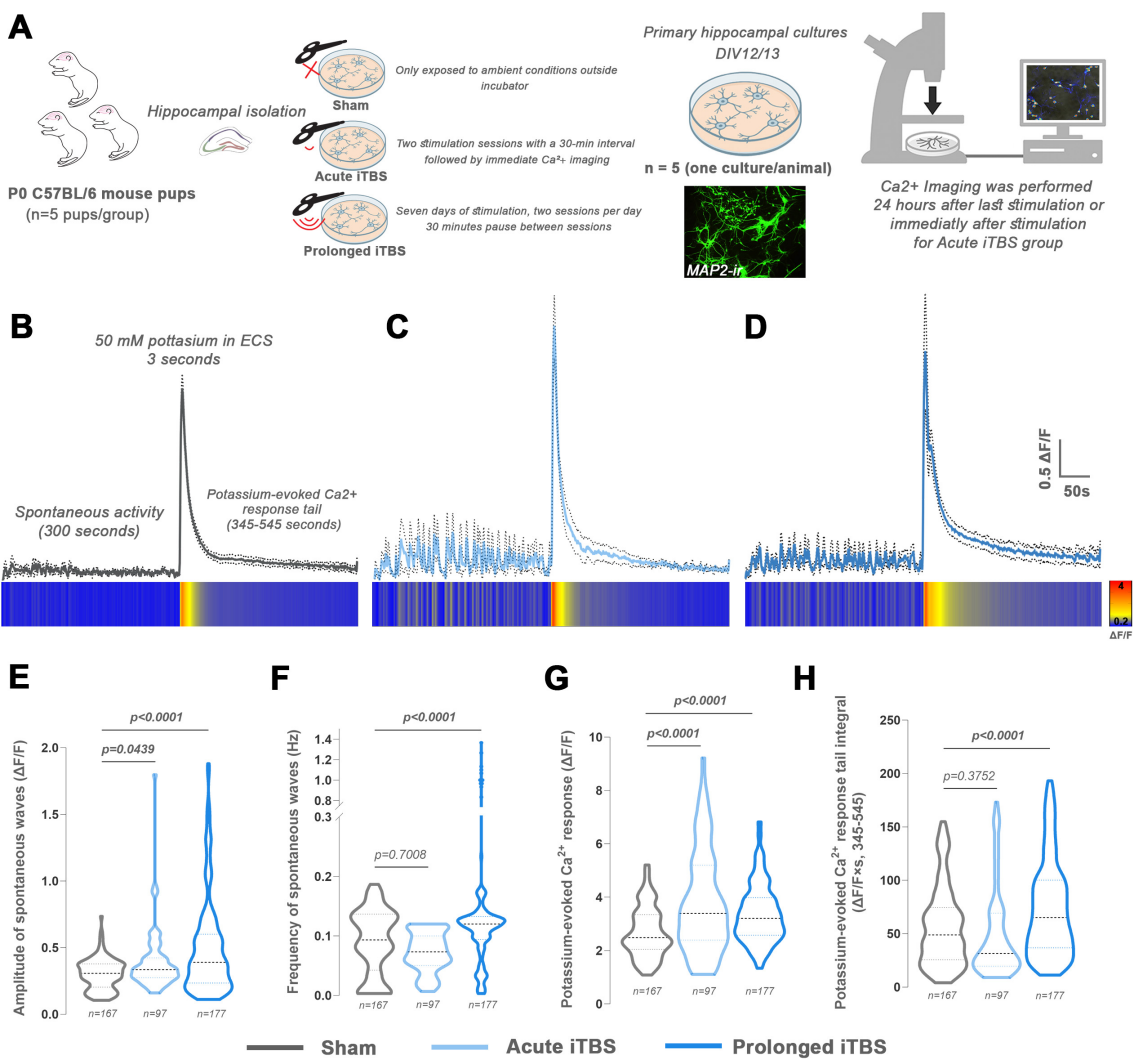
Experimental design and calcium imaging of primary hippocampal mouse cultures after acute and prolonged iTBS600 stimulation. **(A)** *In vitro* study design with experimental groups and timeline. After the hippocampi isolation, the primary cultures were randomly distributed into three groups (*n* = 5 animals per group, one culture per animal). The acute group was stimulated on day *in vitro* (DIV) 12 and immediately used for calcium imaging, while the prolonged group began stimulation on DIV6 and was used for experiments 24 h after the last stimulation (DIV12/13). The Sham group was kept outside the incubator next to the stimulated petri dishes but was not exposed to magnetic stimulation and was used for experiments at DIV12/13. Fluorescent micrograph shows MAP2 immunostaining of primary hippocampal neurons. **(B–D)** Representative graphic changes in calcium dynamics observed for 600 s, including first 300 s of spontaneous activity and next 300 s of potassium-induced activity, are shown for **(B)** Sham, **(C)** acute and **(D)** prolonged group as fluorescence data normalized by the change in fluorescence relative to the baseline. Heat-map plots are given below the corresponding graphs. **(E–H)** Parameters investigated in calcium imaging analysis include the **(E)** amplitude of spontaneous waves, **(F)** frequency of spontaneous waves, **(G)** potassium-evoked calcium response and **(H)** potassium-evoked calcium response tail integral. Numbers at the bottom of the graphs show the number of cells included in the analysis. Data were analyzed using one-way ANOVA or two-way ANOVA (mixed-design) followed by Dunnet’s or Tuckey’s *post-hoc* test for multiple comparisons, respectively. Data are expressed as mean ± SD. Significance is shown on graphs as *p*-value, in bold for *p* < 0.05.

As synaptosomal protein expression resembled that typically observed in LTP-like plasticity, Ca^2+^ dynamics *in vitro* was examined. To evaluate the effects of iTBS on Ca^2+^ dynamics, primary culture of hippocampal neurons was subjected to acute or prolonged stimulation protocol (Figure 5A). Changes in Ca^2+^ dynamics were monitored for 600 s. During the first 300 s, spontaneous activity was recorded, followed by monitoring of K^+^-induced responses at 300 s (Figures 5B–D). When compared to the Sham group (Figure 5B), Ca^2+^ recordings from both the acute (Figure 5C) and prolonged (Figure 5D) stimulated cultures showed an increase in spontaneous activity as well as in the amplitude of K^+^-induced peak. Notably, prolonged stimulation resulted in observable differences in peak decay kinetics (Figure 5D, heat-map plots). Specifically, the amplitude of spontaneous activity [Figure 5E, *F*_(2, 379)_ = 14.60, *p* < 0.0001] was significantly increased in both the acute (*p* = 0.0439) and prolonged (*p* < 0.0001) groups relative to Sham, whereas the frequency of spontaneous activity [Figure 5F, *F*_(2, 379)_ = 14.60, *p* < 0.0001] was increased only in the prolonged group compared to Sham (*p* < 0.0001). Furthermore, the amplitude of K^+^-induced Ca^2+^ peak [Figure 5G, *F*_(2, 423)_ = 25.42, *p* < 0.0001], was increased following both acute (*p <* 0.0001) and prolonged iTBS (*p <* 0.0001) compared to Sham. Finally, the prolonged Ca^2+^ response tail [Figure 7H, *F*_(2, 423)_ = 13.4, *p* < 0.0001] following K^+^ stimulation was significantly longer and of higher intensity compared to Sham (*p* < 0.0001), while no effect of acute stimulation was detected (*p* = 0.3752).

### 3.8 iTBS-induced changes in Ca^2+^ dynamics are partially dependent on NMDA receptor activation

**FIGURE 6:**
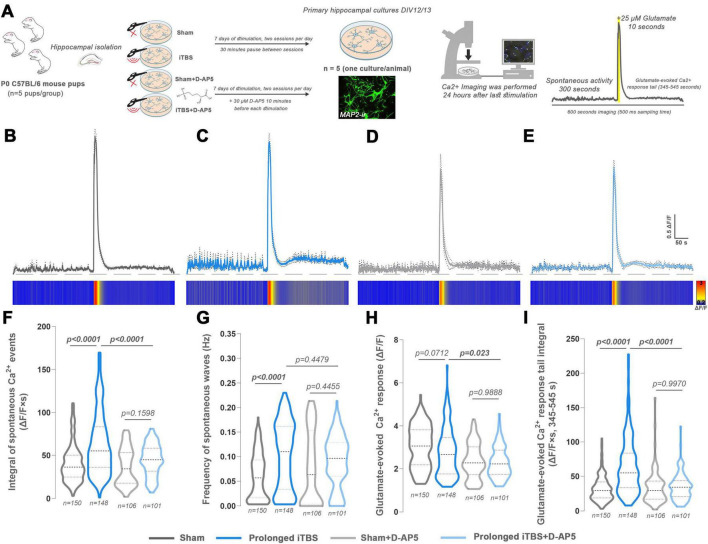
Experimental design and calcium imaging of D-(-)-2-Amino-5-phosphonopentanoic acid (D-AP5)-treated primary hippocampal mouse cultures after prolonged iTBS600 stimulation. **(A)** *In vitro* study design with experimental groups and timeline. After the hippocampi isolation, the primary cultures were randomly distributed into four experimental groups (*n* = 5 animals per group, one culture per animal). The prolonged group began stimulation on DIV6 and was used for experiments 24 h after the last stimulation (DIV12/13). The Sham group was kept outside the incubator next to the stimulated petri dishes but was not exposed to magnetic stimulation and was used for experiments at DIV12/13. For both iTBS+D-AP5 and Sham+D-AP5 groups, the cultures were treated with 30 μM D-AP5 10 min before the first session of stimulation each day. Fluorescent micrograph shows MAP2 immunostaining of primary hippocampal neurons. Representative graphic indicates imaging flow, where the spontaneous activity was observed during the first 300 s, followed by a 300 s period of 25 μ glutamate-induced activity. **(B–E)** Representative graphic changes in calcium dynamics are shown for **(B)** Sham, **(C)** prolonged iTBS, **(D)** Sham+D-AP5 and **(E)** prolonged iTBS+D-AP5 groups as fluorescence data normalized by the change in fluorescence relative to the baseline. Heat-map plots are given below the corresponding graphs. **(F–I)** Parameters investigated in calcium imaging analysis include the **(F)** integral of spontaneous calcium events, **(G)** frequency of spontaneous waves, **(H)** glutamate-evoked calcium response and **(I)** glutamate-evoked calcium response tail integral. Numbers at the bottom of the graphs show the number of cells included in the analysis. Data were analyzed using one-way ANOVA or two-way ANOVA (mixed-design) followed by Dunnet’s or Tuckey’s *post-hoc* test for multiple comparisons, respectively. Data are expressed as mean ± SD. Significance is shown on graphs as *p*-value, in bold for *p* < 0.05.

Given that synaptosomal analysis revealed alterations in glutamatergic signaling, and that prolonged iTBS affected Ca^2+^ homeostasis, the next step was to assess whether the cellular response to glutamate was also modified, and to what extent NMDA receptors contributed to this effect. To this end, D-(-)-2-Amino-5-phosphonopentanoic acid (D-AP5), a selective NMDA receptor antagonist, was applied 10 min prior to each stimulation and this was repeated throughout the entire 7-day stimulation period (Figure 6A), resulting in four experimental groups—Sham (Figure 6B), Prolonged iTBS (Figure 6C), Sham+D-AP5 (Figure 6D) and Prolonged iTBS+D-AP5 (Figure 6E). The integral of spontaneous waves [Figure 6F, Stimulation: *F*_(1, 524)_ = 41.25, *p* < 0.0001, NMDA antagonism: *F*_(1, 524)_ = 23.95, *p* < 0.0001, Interaction: *F*_(1, 524)_ = 9.87, *p* = 0.018] was significantly increased in prolonged iTBS when compared to Sham (*p <* 0.0001), confirming the effects observed in previous experiments. Notably, application of D-AP5 abolished this effect, as the iTBS+D-AP5 group showed a significantly reduced response compared to iTBS alone group (*p <* 0.0001). As observed in previous experiments, prolonged iTBS increased the frequency of spontaneous Ca^2+^ waves [Figure 6G, Stimulation: *F*_(1, 518)_ = 23.60, *p* < 0.0001, NMDA antagonism: *F*_(1, 518)_ = 0.305, *p* = 0.5809, Interaction: *F*_(1, 518)_ = 6.643, *p* = 0.0102] compared to Sham (*p <* 0.0001). However, D-AP5 had no significant effect on this parameter, as the frequency in the iTBS+D-AP5 group remained nearly identical to that in the iTBS group (*p* = 0.4455). Interestingly, glutamate-evoked Ca^2+^ peak [Figure 6H, Stimulation: *F*_(1, 504)_ = 3.131, *p* = 0.0774; NMDA antagonism: *F*_(1, 504)_ = 39.43, *p* < 0.0001; Interaction: *F*_(1, 504)_ = 1.617, *p* = 0.2041] was not significantly different between prolonged iTBS and Sham groups (*p* = 0.0712). However, chronic application of D-AP5 prior to prolonged stimulation significantly reduced the amplitude of the peak in both Sham and iTBS groups (*p* = 0.023), indicating a partial contribution of the NMDA receptors to the glutamate-evoked response. Finally, as NMDA receptor activity may contribute to the extended phase of glutamate-induced Ca^2+^ responses, we examined whether this component is modulated by iTBS [Figure 6I, Stimulation: *F*_(1, 504)_ = 45.43, *p* < 0.0001; NMDA antagonism: *F*_(1, 504)_ = 32.48, *p* < 0.0001; Interaction: *F*_(1, 504)_ = 41.29, *p* < 0.0001]. As expected, iTBS significantly increased the amplitude of the tail-like Ca^2+^ response compared to Sham (*p* < 0.0001, Figure 6B, heat-map plot), while this effect was abolished by D-AP5, suggesting that prolonged iTBS modulates Ca^2+^ dynamics at least in part through enhanced activation of NMDA receptors.

### 3.9 GluN2A subunit is necessary for iTBS-induced changes in Ca^2+^ dynamics

**FIGURE 7:**
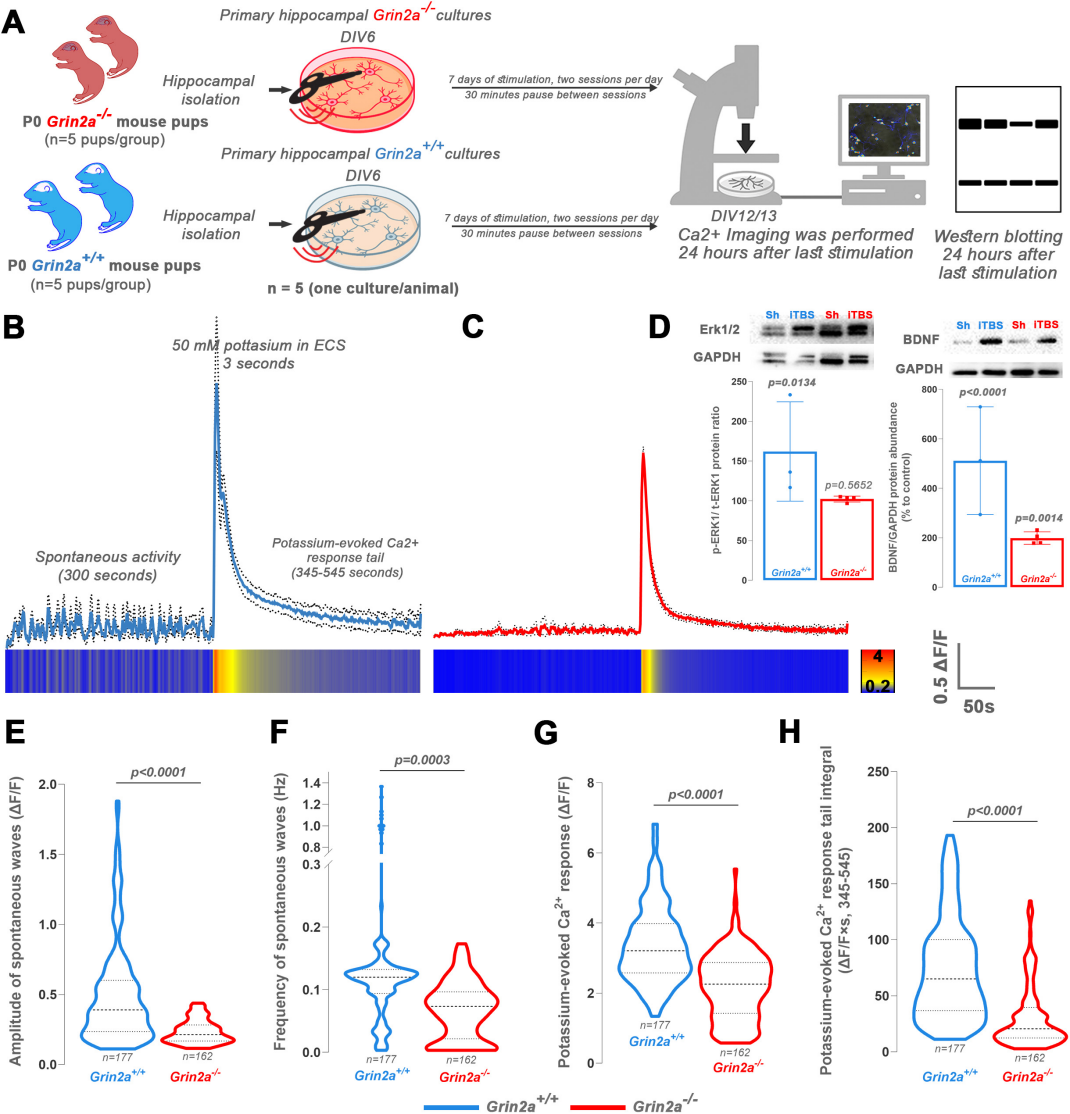
Experimental design, calcium imaging and western blot of wild-type (WT) and *Grin2a -/-* (KO) primary hippocampal mouse cultures after the prolonged iTBS600. **(A)** *In vitro* study design with experimental groups and timeline. After the hippocampi isolation, the primary cultures were randomly distributed into four experimental groups (Sham groups not shown) (*n* = 5 animals per group, one culture per animal). Both prolonged groups began stimulation on DIV6 and were used for experiments 24 h after the last stimulation (DIV12/13). The Sham groups were kept outside the incubator next to the stimulated petri dishes but were not exposed to magnetic stimulation and were used for experiments at DIV12/13. **(B,C)** Representative graphic changes in calcium dynamics observed for 600 s, including the first 300 s of spontaneous activity and next 300 s of potassium-induced activity, shown for **(B)** WT and **(C)** KO group as fluorescence data normalized by the change in fluorescence relative to the baseline. Heat-map plots are given below the corresponding graphs. **(E–H)** Parameters investigated in calcium imaging analysis include the **(E)** amplitude of spontaneous waves, **(F)** frequency of spontaneous waves, **(G)** potassium-evoked calcium response and **(H)** potassium-evoked calcium response tail integral. Numbers at the bottom of the graphs show the number of cells included in the analysis. Data were analyzed using two-tailed Student’s *t*-test. Data are expressed as mean ± SD. **(D)** Western blot analysis of phospho/total-ERK and BDNF expression in hippocampal cultures with their respective GAPDH (graphs represent change in respect to WT or KO Sham). The sample layout is indicated above the membranes (Sh—Sham). Dots in the graphs represent the values of individual animals. Obtained results were analyzed using two-tailed Student’s *t*-test. Data are expressed as arbitrary units derived from optical density ± SD. Significance is shown on graphs as *p*-value, in bold for *p* < 0.05.

Since the GluN2A subunit was significantly upregulated in synaptosomes following prolonged iTBS, we next examined whether this subunit is required for iTBS-induced effects by using *Grin2a* knockout cultures *in vitro* (Figure 7A). All analyzed parameters were markedly reduced in KO iTBS (Figure 7C) cultures compared to wild-type iTBS (Figure 7B) stimulated for 7 days, including the amplitude of spontaneous Ca^2+^ waves (Figure 7E, *t* = 6.039, d_*f*_ = 243, *p* < 0.0001), frequency of spontaneous waves (Figure 7F, *t* = 3.701, d_*f*_ = 243, *p* = 0.0003), potassium-evoked Ca^2+^ peak (Figure 7G, *t* = 10.65, d_*f*_ = 337, *p* < 0.0001), and the integral of the potassium-evoked Ca^2+^ response tail (Figure 7H, *t* = 10.68, d_*f*_ = 337, *p* < 0.0001). Finally, immunoblotting was performed on these cultures to assess the expression levels of ERK1/2 and BDNF, both of which were upregulated in synaptosomes following iTBS. In *Grin2a* KO cultures, ERK1/2 expression remained unchanged after stimulation (Figure 7D, *t* = 0.5949, d_*f*_ = 10, *p* = 0.5652), whereas a clear upregulation was observed in WT cultures (Figure 7D, *t* = .260, d_*f*_ = 8, *p* = 0.0134). Notably, BDNF expression (Figure 7D) was robustly increased in WT cultures (∼5.5-fold, *t* = 10.37, d_*f*_ = 8, *p <* 0.0001), but only modestly elevated (∼2-fold, *t* = 4.375, d_*f*_ = 10, *p* = 0.0014) in KO cultures, indicating that GluN2A contributes to the molecular effects of iTBS, particularly in BDNF signaling.

### 3.10 Prolonged iTBS selectively affects action potential properties without changing intrinsic excitability of primary hippocampal neurons

**FIGURE 8:**
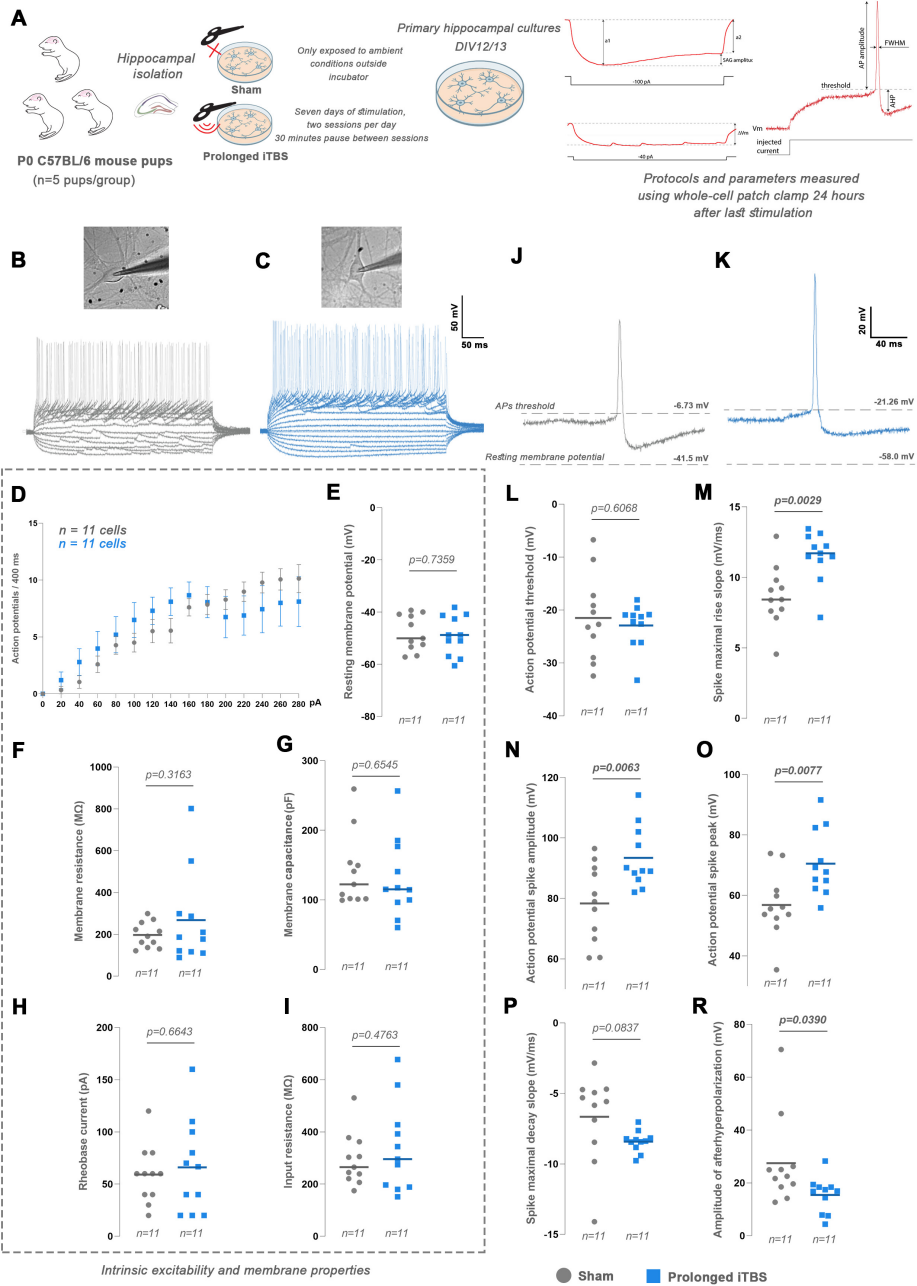
Experimental design and patch-clamp analysis of wild-type (WT) primary hippocampal mouse cultures after prolonged iTBS600. **(A)** *In vitro* study design with experimental groups and timeline. After the hippocampi isolation, the primary cultures were randomly distributed into two experimental groups (*n* = 5 animals per group, one culture per animal). The prolonged iTBS group began stimulation on DIV6 and was used for experiments 24 h after the last stimulation (DIV12/13). The Sham group was kept outside the incubator next to the stimulated petri dishes but was not exposed to magnetic stimulation and was used for experiments at DIV12/13. **(B,C)** Representative current-clamp figures obtained over 400 ms from a series of current injections ranging from -100 pA to +280 pA in 20 pA increments along with brightfield images of **(B)** Sham and **(C)** prolonged iTBS cells. **(D)** Graph showing the number of evoked action potentials at each current step during the 400 ms period. **(E–I)** Parameters describing intrinsic membrane properties, including **(E)** resting membrane potential, **(F)** membrane resistance, **(G)** membrane capacitance, **(H)** rheobase current and **(I)** input resistance. **(J,K)** Representative action potential graphs with values for action potential thresholds and resting membrane potentials for **(J)** Sham and **(K)** prolonged iTBS. **(L–R)** Parameters describing properties of action potentials (APs), including **(L)** AP threshold, **(M)** spike maximal rise slope, **(N)** AP spike amplitude, **(O)** AP spike peak, **(P)** spike maximal decay slope and **(R)** amplitude of afterhyperpolarization. Dots and squares in the graphs represent the values of individual cells. Numbers at the bottom of the graphs show the number of cells included in the analysis. Data were analyzed using two-tailed Student’s *t*-test. Data are expressed as mean ± SEM. Significance is shown on graphs as *p*-value, in bold for *p* < 0.05.

To evaluate the effects of prolonged iTBS on intrinsic neuronal properties and excitability, we performed whole-cell patch clamp recordings in primary hippocampal neurons (Figure 8A). Step-wise 20 pA current injection evoked typical APs in both Sham (Figure 8B) and prolonged iTBS neurons (Figure 8C), with no difference in the number of spikes generated at equivalent depolarizing currents (Figure 8D). Furthermore, no significant differences were observed in the parameters describing intrinsic membrane properties, including resting membrane potential (Figure 8E, *t* = 0.5229, d_*f*_ = 20, *p* = 0.7359), membrane resistance (Figure 8F, *t* = 1.028, d_*f*_ = 20, *p* = 0.3163), membrane capacitance (Figure 8G, *t* = 0.4543, d_*f*_ = 20, *p* = 0.6545), rheobase (Figure 8H, *t* = 0.4406, d_*f*_ = 20, *p* = 0.6643), and input resistance (Figure 8I, *t* = 0.7260, d_*f*_ = 20, *p* = 0.4763). On the other hand, prolonged iTBS (Figure 8K) significantly changed properties of action potentials compared to Sham (Figure 8J). Although the action potential threshold was unaffected by iTBS (Figure 8L, *t* = 0.5229, d_*f*_ = 20, *p* = 0.6068), iTBS significantly increased the maximal rise slope (Figure 8M, *t* = 3.398, d_*f*_ = 20, *p* = 0.0029), spike amplitude (Figure 8N, *t* = 3.054, d_*f*_ = 20, *p* = 0.0063), peak (Figure 8O, *t* = 2.962, d_*f*_ = 20, *p* = 0.0077), with concomitant reduction in afterhyperpolarization amplitude (Figure 8R, *t* = 2.209, d_*f*_ = 20, *p* = 0.0390), while the maximal decay slope showed a trend toward significance (Figure 8P, *t* = 1.820, d_*f*_ = 20, *p* = 0.0837).

### 3.11 Prolonged iTBS produces long-lasting after-effects that possibly facilitate maturation and stabilization of immature synapses

**FIGURE 9:**
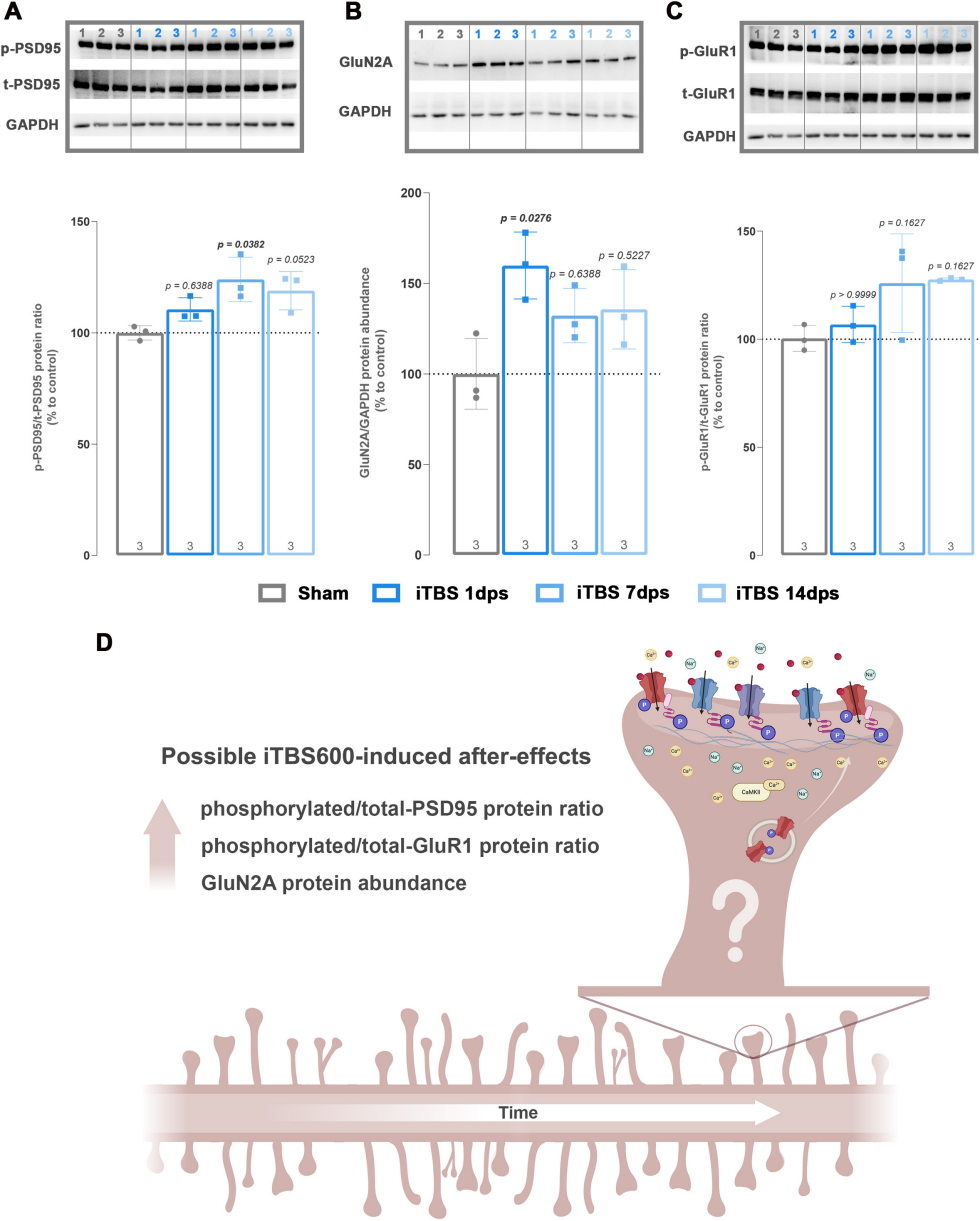
Western blot analysis of postsynaptic components in three different timepoints following prolonged iTBS600 in hippocampal synaptosomal fraction. Graphs and representative membranes of **(A)** phospho/total-PSD95, **(B)** GluN2A, **(C)** phospho/total-GluR1 with their respective GAPDH. **(D)** Schematic representation of possible iTBS600-induced after-effects, indicating enhancements in synaptic maturation and stabilization over time. Numbers above the membranes show the sample layout. Dots in the graphs represent the values of individual animals. Numbers at the bottom of the graphs show the number of animals included in the analysis (*n* = 3 animals per group). Obtained results were analyzed using Kruskal-Wallis test followed by Dunnett’s *post-hoc* analysis. Data are expressed as arbitrary units derived from optical density ± SD. Significance is shown on graphs as *p*-value, in bold for *p* < 0.05. Created in BioRender. Cosic, T. (2026). https://BioRender.com/zf3r2cx.

To evaluate whether prolonged iTBS600 produces any lasting after-effects, we performed a preliminary study on isolated synaptosomes at 7- (7 dps) and 14 (14 dps) days after last stimulation. The pattern of expression for phosphorylated PSD-95 (*Ser295*) (Figure 9A, Kruskal–Wallis test: *H* = 8.12, *p* = 0.0152) showed an increase at 7 dps (*p* = 0.0382) which was also observed at 14 dps (*p* = 0.0523). GluN2A expression (Figure 9B, Kruskal–Wallis test: *H* = 6.79, p = 0.0536) showed an increase at 1 dps (*p* = 0.0276) that was maintained at 7 and 14 dps, however, this effect did not reach statistical significance. Furthermore, phosphorylated GluR1 (Figure 9C, Kruskal–Wallis test: *H* = 5.97, *p* = 0.10) showed increase of about 20 % at both 7 and 14 dps, however, due to the small sample size, this effect did not reach statistical significance. Finally, synaptophysin expression (Supplementary Figure 5, Kruskal–Wallis test: *H* = 9.42, *p* = 0.0014) followed a dynamic, biphasic pattern, increasing at 1 dps, transiently decreasing at 7 dps, and rising again at 14 dps relative to Sham. Graphical summary of temporal changes and their implications are shown in Figure 9D.

## 4 Discussion

The present study investigated the effect of FDA-approved iTBS600 protocol on hippocampal structural and functional plasticity in healthy animals. Our data demonstrated that a 7-day iTBS600 protocol, applied twice per day, does not affect animal behavior; however, it significantly enhances structural and functional plasticity. This enhancement is reflected by an increase in the number of thin, “*learning*” spines, alterations in electrophysiological properties and Ca^2+^ dynamics, and upregulation of GluN2A- and BDNF-mediated signaling, which in turn activates downstream mTOR-mediated translational machinery. Although it may seem counterintuitive to investigate iTBS in healthy animals, such studies are highly relevant, as they provide a controlled environment to assess the effects of iTBS without the confounding factors associated with disease models (Löscher, 2024), and help establish a baseline physiological response. Since behavioral changes are considered a primary indicator of the effectiveness of nearly all therapeutic interventions, they serve as a critical outcome measure for evaluating treatment efficacy. Despite the proven anxiolytic (Zhang et al., 2023) and cognitive/memory-enhancing potential of iTBS (Pabst et al., 2022; Stekic et al., 2022) in various models of neuropsychiatric and age-related neurodegenerative disorders, our data indicate that, in healthy animals, prolonged iTBS600 does not significantly affect general locomotor or exploratory activity. The somewhat decreased performances in the OFT, observed in both Sham and iTBS group following treatment, may be explained by a re-test effect (Rittweger et al., 2021). In line with a few other studies on healthy animals, iTBS did not appear to influence short-term recognition memory (Rittweger et al., 2021; Wu et al., 2024). We speculate that the absence of observable effects here may be, at least partially, attributed to the fact that the animals used were healthy young adults, likely at the peak of their memory performance (Fukui et al., 2001). Furthermore, in addition to the hippocampus, the perirhinal cortex is also critically involved in object recognition (Murray and Richmond, 2001). Although these structures are highly interconnected, perirhinal cortex primarily contributes to recognition memory over short retention intervals (∼3 h), whereas the hippocampus plays a more prominent role in maintaining long-term (∼24 h) object memory (Hammond et al., 2004; Reger et al., 2009). Given that animals were tested ∼2 h following the sampling phase, and considering the anatomical position of the perirhinal cortex which likely received little to no effective direct stimulation due to its depth, orientation, and the stimulation intensity applied (Zeljkovic Jovanovic et al., 2023), the influence of iTBS600 on memory performance may have been limited. Alternative behavioral paradigms with greater sensitivity to subtle memory changes, or those more specifically reliant on hippocampal processing, may be more appropriate for detecting potential iTBS-induced effects. Furthermore, in the absence of explicit learning or memory demands, newly formed synaptic connections may not be effectively stabilized and, consequently, may fail to translate into measurable behavioral outcomes (Wu et al., 2024; Xu et al., 2009). In this context, enhanced plasticity alone may be insufficient unless it is engaged by tasks that recruit the relevant neural circuits. This hypothesis, however, remains to be directly tested using the abovementioned experimental approaches. Overall, these behavioral observations align with previous reports suggesting that iTBS effects may be more pronounced under pathological conditions (Jemna et al., 2024), where synaptic function and behavioral outcomes are already impaired. However, the maintenance of normal social behavior, weight gain, and grooming patterns support the safety and tolerability of the stimulation protocol, showing that iTBS600 does not disrupt normal function in healthy animals.

Although behavioral outcomes remained largely unchanged, iTBS600 induced significant structural remodeling in the hippocampus, specifically in the CA1 subfield. It has been demonstrated that various rTMS protocols, including iTBS, increase the expression of immediate early genes such as c-Fos (Hoppenrath and Funke, 2013). Therefore, c-Fos immunoreactivity as a correlate of neuronal activity (Bullitt, 1990) was used to identify potential region of interest for subsequent analyses. Even though multiple studies report robust increase in c-Fos expression following iTBS stimulation (Aydin-Abidin et al., 2008; Volz et al., 2013), the modest increase in CA1 subfield may be explained by the time delay from the last stimulation. Namely, the increase in the expression of c-Fos is most prominent between 30 and 90 min after stimulation (Hoppenrath and Funke, 2013), although it may persist for up to 24 h, albeit mainly in glial cells (Lara Aparicio et al., 2022). The rationale for examining c-Fos expression ∼24–25 h after the final stimulation was to avoid capturing acute responses of the last session and identify potential cumulative effects instead. Notably, the CA1 subfield still exhibited elevated c-Fos expression, indicating sustained neuronal activation. Indeed, spine analysis revealed a ∼20% increase in basal dendritic spine density in CA1 pyramidal neurons, accompanied by a higher proportion of thin, immature spines typically associated with synaptic plasticity and dynamic remodeling, also called “learning” spines (Bourne and Harris, 2007). Given the sustained neuronal activation in CA1 and enhanced structural synaptic remodeling, we next examined the local inhibitory parvalbumin-positive (PV^+^) interneurons and their surrounding perineuronal nets, which are key regulators of excitatory-inhibitory balance and plasticity gating (Pizzorusso et al., 2002; Yamada et al., 2015). Previous studies have shown that a single session of iTBS600 induces a marked reduction in cortical parvalbumin expression, with the effect being most pronounced in early adulthood (PD90) (Mix et al., 2015). In the hippocampus, most PV^+^ interneurons are enwrapped by PNNs, which contribute to synaptic stabilization and control plasticity (Fawcett et al., 2022). Attenuation or degradation of PNNs is generally associated with increased network activity, likely due to reduced inhibitory control (Gottschling et al., 2019). Importantly, in the hippocampal CA1 subfield, enzymatic degradation or genetic depletion of PNN components has been shown to impair LTP (Bukalo et al., 2001; Kochlamazashvili et al., 2010; Shi et al., 2019). Our findings can be interpreted within the framework of the *stability–plasticity trade-off*. While iTBS induces LTP-like plasticity and increases the number of “learning” spines, the concurrent increase in PNN expression suggests that PNNs do not merely act as brakes on plasticity, but rather stabilize newly formed synapses and prevent excessive plasticity. The mechanisms underlying PNN regulation are activity-dependent. Neuronal activity promotes PNN formation and maturation, as shown by reductions in PNNs following sensory deprivation in the visual cortex and enhancement following depolarization *in vitro* (Brückner and Grosche, 2001). Pharmacological studies indicate that this process relies on Ca^2+^ influx through L-type voltage-gated calcium channels, NMDA receptors, and Ca^2+^-permeable AMPA receptors (Dityatev et al., 2007; Dziembowska et al., 2012). In the context of prolonged iTBS600, the observed greater condensation of PNNs across all CA subfields, together with a slight reduction in PV expression, may represent a compensatory response aimed at maintaining excitatory–inhibitory balance. These changes may constitute coordinated homeostatic response orchestrated by activity-dependent Ca^2+^ signaling. By limiting excessive structural plasticity and stabilizing newly formed synapses, this dual effect could prevent hyperexcitability of the networks while preserving their functional gains.

The observed increase in dendritic spine density and their morphological reshaping, as well as formation of new synapses, likely depend on signals that drive actin cytoskeleton remodeling and local protein synthesis, most commonly initiated by Ca^2+^ influx through NMDAR and/or activation of BDNF–TrkB signaling pathways (Popovic and Dragic, 2025; Zagrebelsky et al., 2020). It is well-accepted that the effects of iTBS are mediated by NMDAR in both human subjects and experimental animals (Brown et al., 2022; Huang et al., 2007). Moreover, it has been shown that even a single iTBS600 stimulation is sufficient to induce synaptic potentiation in CA1 neurons lasting for up to 8 h post-stimulation (Vlachos et al., 2012). Our findings extend this knowledge by demonstrating that 7-day iTBS600 modulates glutamatergic synaptic signaling, specifically through increased GluN1/GluN2A receptor expression and upregulation of glutamate transporters EAAT1 and EAAT2 (Zeljkovic Jovanovic et al., 2023) and downregulation of VGLUT1. This may indicate an alteration in calcium homeostasis and enhanced glutamate clearance, possibly reflecting a compensatory mechanism aimed at maintaining synaptic stability and preventing excitotoxicity (Cao et al., 2022; Wilson et al., 2005). In line with this finding, we observed a robust activation of downstream BDNF-TrkB signaling pathways, as evidenced by increased expression of BDNF and elevated phosphorylation of Akt, ERK1/2, and mTOR kinases, which are well-known mediators of synaptic growth and protein synthesis (Kumar et al., 2005). In particular, acute BDNF treatment in hippocampal cultures has been found to upregulate the protein expression of GluN1, GluN2A and GluN2B subunits, along with promoting their trafficking to the plasma membrane, indicating increased NMDAR activity (Caldeira et al., 2007), and supporting our findings of iTBS-induced enhancement of BDNF and subsequent NMDAR subunits expression. These molecular changes likely underpin the observed enhancement in structural plasticity, including increased density of thin, branched, and filopodial dendritic spines in CA1 pyramidal neurons, which are characteristic of learning-related synaptic remodeling. Our calcium imaging data further corroborate these findings, confirming previous data using acute iTBS600 (Lu et al., 2025), and additionally show that prolonged iTBS600 increases spontaneous and evoked Ca^2+^ responses in cultured primary hippocampal neurons. Notably, the prolonged stimulation led to a sustained Ca^2+^ response tail following K^+^-induced depolarization with altered decay kinetics, persisting 24 h after the final stimulation, thereby revealing a lasting perturbation of calcium dynamics that may underlie and potentially facilitate the observed synaptic modifications *ex vivo*. Vlachos et al. provided compelling evidence that acute 10 Hz repetitive magnetic stimulation (rMS) elicits structural and functional plasticity at excitatory postsynaptic sites, a phenomenon that critically depends, at least in part, on the activation of NMDAR during the stimulation period (Vlachos et al., 2012). To further elucidate this finding, we investigated whether nonspecific antagonism of NMDAR could prevent the effects of prolonged iTBS600 on Ca^2+^ dynamics. Indeed, a 7-day pre-treatment with the competitive NMDAR antagonist D-AP5, followed by iTBS600 stimulation, abolished the enhancements in both spontaneous and glutamate-induced Ca^2+^ responses in cultured primary hippocampal neurons, strongly indicating that, similar to 10 Hz rMS (Vlachos et al., 2012), the effects of iTBS600 are at least partially mediated by NMDAR activation. We further speculate that the sustained Ca^2+^ response tail induced by iTBS600 is primarily driven by prolonged activation of glutamatergic receptors, particularly AMPARs and NMDARs (Vlachos et al., 2012), as their pharmacological blockade significantly reduced or even abolished Ca^2+^ response tail (Li et al., 2017). At this moment we cannot exclude the possibility that Ca^2+^ may also be released from internal stores, as this requires further experiments, but it has been shown that treatment with ryanodine and thapsigargin did not affect calcium waves and dynamics following glutamate exposure *in vitro* (Clodfelter et al., 2002), although it is important for hippocampal LTP (Harvey and Collingridge, 1992).

Even though both GluN2B- and GluN2A-containing NMDAR contribute to the induction of LTP, a principal after-effect of rTMS/iTBS, growing evidence points to a predominant role of the GluN2A subunit in initiating and sustaining hippocampal LTP and structural plasticity (Ahnaou et al., 2021; Li et al., 2007; Monfort and Felipo, 2007; Südkamp et al., 2024). Given that prolonged iTBS600 treatment increased synaptosomal levels of GluN2A, and that nonspecific NMDAR blockade abolished iTBS-induced changes in Ca^2+^ dynamics, we next sought to determine whether GluN2A is required for specific effects of iTBS. To address this, we employed Grin2a knockout model to assess whether the absence of this subunit would mimic the effects of nonspecific NMDAR inhibition on iTBS-induced Ca^2+^ dynamics. Existing evidence indicates that in Grin2a^+^/^+^ mice, the threshold for LTP induction is elevated, and both LTP and LTD are impaired within the CA1 hippocampal subfield (Kannangara et al., 2015; Sakimura et al., 1995). Our experiments with Grin2a^+^/^+^ hippocampal neuronal cultures revealed that the GluN2A subunit is essential for the full spectrum of iTBS-induced effects, as stimulated *Grin2a*^+^/^+^ cultures exhibited Ca^2+^ dynamics similar to WT/KO Sham controls, with lower evoked Ca^2+^ peaks, and failed to display key synaptic and molecular adaptations, including BDNF upregulation and ERK1/2 phosphorylation, although detected increase in BDNF points toward GluN2A-independent pathways. It has been shown that CA1 pyramidal neurons of *Grin2a^+^/^+^* animals exhibit a reduced frequency of miniature excitatory postsynaptic currents, decreased proximal dendritic density, a lower NMDAR-to-AMPAR current ratio, and a slower decay time constant of NMDA receptor mediated EPSCs (Yasmin et al., 2025). Motivated by these findings, we used whole-cell patch clamp recordings to assess the impact of prolonged iTBS600 on intrinsic neuronal properties and excitability. Previous studies on layer V pyramidal neurons have shown that acute low-intensity iTBS600 does not affect passive membrane properties but hyperpolarizes the AP threshold and increases evoked spike-firing frequency (Tang et al., 2016) pointing toward its effect on voltage-gated ion channels. Thus far, only one study applied a 7-day protocol of 1 Hz rTMS on CA1 pyramidal neurons and similarly, found no changes in intrinsic neuronal excitability, but changed AP properties and faster recovery rate (Tan et al., 2013). Consistent with these reports, our findings demonstrate that prolonged iTBS600 does not alter passive membrane properties or overall intrinsic excitability, but selectively modifies AP parameters, such as reducing afterhyperpolarization amplitude, which may facilitate the generation of subsequent action potentials. Moreover, reduced afterhyperpolarization has been linked to enhanced susceptibility for LTP induction (Sah and Bekkers, 1996). Although we did not directly investigate the underlying mechanisms, we speculate that the observed changes in the AHP are unlikely to result from alterations in voltage-gated K^+^ channels, as the decay slope remained unchanged (Kimm et al., 2015). Instead, these effects may be mediated by Ca^2+^-activated K^+^ (SK) channels, as they underlie the AHP which regulates APs and limits the firing frequency of repetitive action potentials (for review please see Kuiper et al., 2012). This can also be viewed in the context of periods of heightened synaptic activity, particularly during LTP, when SK2 channels undergo activity-dependent redistribution, not only presynaptically, but also within dendritic spines, through phosphorylation-mediated internalization, resulting in enhanced postsynaptic potentials (Hammond et al., 2006; Lin et al., 2008). This mechanism, however, remains to be experimentally validated in our system. The observation that iTBS600 affects spike dynamics without altering firing frequency may be indicative for synaptic plasticity rather than driving hyperexcitability, which aligns with modified synaptic expression of EAAT1/2 and VGLUT1 observed *ex vivo*. Given that iTBS also induced GluN2A upregulation, enhanced Ca^2+^ signaling, and promoted BDNF–Akt/Erk/mTOR pathway activation, it is plausible that modulation of action potential properties represents a downstream consequence of NMDAR-dependent signaling, further supporting the role of GluN2A-containing receptors and enhanced Ca^2+^ dynamics as central mediators of iTBS-induced neuroplasticity (Banerjee et al., 2017; Palmer et al., 2014).

One of the main advantages of iTBS in clinical settings is its ability to induce lasting after-effects (Cirillo et al., 2017). Accordingly, we sought to explore the temporal dynamics of these after-effects, however, due to the limited number of animals, the present observations should be interpreted as indicative of potential trends rather than statistically-supported conclusions. It appears that changes in synaptic markers persist for at least 2 weeks following prolonged iTBS600 stimulation. Enhanced phosphorylation of the GluR1 AMPA receptor subunit together with increased PSD-95 phosphorylation suggests activity-dependent synaptic maturation and stabilization (Iasevoli et al., 2013). In particular, phosphorylation of *Ser845* on GluR1 subunit of AMPA receptors has been shown to increase channel opening probability and regulate their expression at the postsynaptic membrane (Banke et al., 2000; Oh et al., 2006). Furthermore, it has been demonstrated that PSD-95 regulates AMPAR delivery during LTP-induced synaptic strengthening in hippocampal synapses, both *in vitro* and *in vivo* (Ehrlich and Malinow, 2004). Specifically, phosphorylation of Ser295 on PSD-95 enhances the synaptic accumulation of PSD-95 and the ability of PSD-95 to recruit surface AMPA receptors and potentiate excitatory postsynaptic currents (Kim et al., 2007), which could be seen as a marker of synaptic maturation (El-Husseini et al., 2000). Given our observation of a modest decrease in synaptophysin levels at 7 dps, it is tempting to speculate that this time window may correspond to the elimination of excess immature synapses. Such a process could potentially involve activity-dependent synaptic pruning mechanisms (Faust et al., 2021), potentially mediated by autophagy-related pathways, as suggested by recent evidence (Wang et al., 2025). However, this interpretation remains speculative, and direct experimental validation is required to establish a causal link between iTBS-induced synaptic remodeling.

**FIGURE 10:**
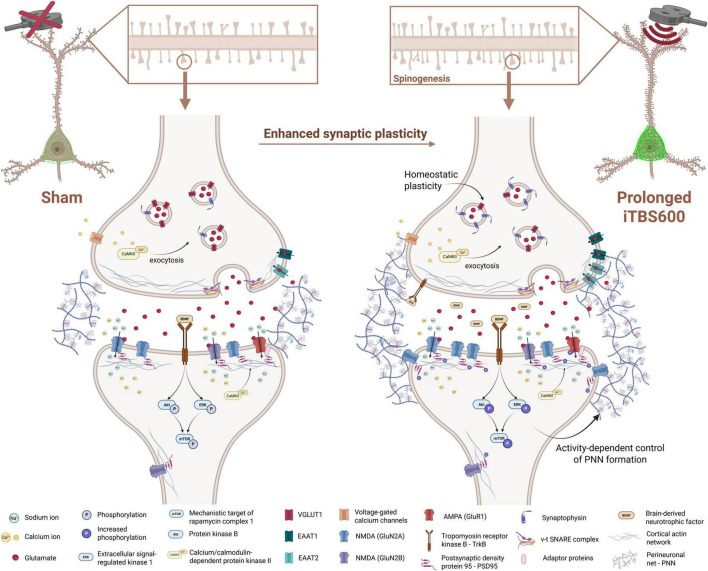
Proposed mechanism of prolonged iTBS600 on synaptic plasticity. Schematic representation of Sham and prolonged iTBS600-stimulated synapses illustrating activity-dependent enhancement of synaptic plasticity. Prolonged iTBS600 is proposed to induce sustained glutamatergic signaling, leading to prolonged Ca^2+^ influx primarily via AMPARs and NMDARs, activation of Ca^2+^-dependent signaling cascades (CaMKII, ERK, Akt–mTOR) and increased BDNF–TrkB signaling. These molecular events promote spinogenesis, remodeling of the actin cytoskeleton, and homeostatic plasticity, accompanied by activity-dependent regulation of perineuronal net (PNN) formation, ultimately supporting strengthened and adaptable synaptic connectivity. Created in BioRender. Cosic, T. (2026). Proposed mechanism of prolonged iTBS600 on synaptic plasticity. https://BioRender.com/ykn3erd.

Together, our findings demonstrate that prolonged iTBS enhances hippocampal synaptic plasticity through coordinated modulation of glutamatergic signaling, calcium dynamics, and neurotrophic pathways, with GluN2A-containing NMDARs emerging as key mediators, with lasting after-effects (Figure 10). Beyond elucidating the biological mechanisms of iTBS600, these results have translational relevance, as several neuropsychiatric and neurological disorders linked to GluN2A mutations (e.g., epileptic seizures, schizophrenia, and developmental delay/intellectual disability) (Elmasri et al., 2022; Shepard et al., 2024) may exhibit reduced responsiveness to iTBS/rTMS-based interventions, which requires further investigation in preclinical and clinical studies in these disease models and disorders, respectively. Finally, our work provides a mechanistic framework to better understand the application of non-invasive brain stimulation in both health and disease.

### 4.1 Study limitations

Several limitations of the present study should be acknowledged. First, despite robust molecular, synaptic, and electrophysiological plasticity induced by iTBS600, we did not observe significant behavioral effects in healthy young animals. This likely reflects ceiling-level performance and limited sensitivity of the employed behavioral paradigms to detect learning and memory enhancements in intact animals. Second, most analyses were performed at a single post-stimulation time point, limiting insight into the temporal trajectory and persistence of the observed synaptic and molecular changes. Third, some experiments relied on a limited number of animals, which may not fully capture biological variability despite extensive within-animal sampling. Fourth, only one sex was included, and given known sex-dependent differences in NMDA receptor signaling (Hönack and Löscher, 1993), synaptic plasticity, and responses to brain stimulation, the generalizability of our findings remains to be explored. Future studies should specifically examine whether female rodents show similar GluN2A-dependent responses, as estrogen is known to modulate NMDA receptor function. Due to the technical limitations associated with the size of the coil, which do not allow focal stimulation of specific areas but can be considered as whole-brain stimulation, the observed effects of iTBS600 may be the result of both cortical and subcortical stimulation and interconnectivity. Addressing these limitations in future studies, particularly through longitudinal, male/female studies, and other learning and memory paradigms, will be essential for fully understanding the functional relevance of iTBS-induced plasticity.

## Author contribution

DP: Formal analysis, Data curation, Investigation, Writing – original draft. MZ: Writing – review & editing, Investigation, Formal analysis. MZJ: Investigation, Writing – review & editing, Formal analysis. MM: Investigation, Funding acquisition, Writing – review & editing, Supervision, Formal analysis. TM: Formal analysis, Writing – review & editing, Investigation. TR: Data curation, Writing – review & editing, Investigation. AS: Writing – review & editing, Formal analysis, Investigation. EG: Data curation, Formal analysis, Writing – review & editing, Investigation. AJ: Writing – review & editing, Formal analysis, Investigation. KM: Writing – review & editing, Investigation. MAB: Formal analysis, Investigation, Writing – review & editing. IS: Writing – review & editing, Funding acquisition, Investigation, Formal analysis. MD: Supervision, Conceptualization, Funding acquisition, Formal analysis, Project administration, Data curation, Writing – original draft.

## Data Availability

The datasets presented in this study can be found in online repositories. The names of the repository/repositories and accession number(s) can be found in this article/Supplementary material.

## Footnotes

1
https://doi.org/10.1038/nmeth.2089

2
https://synapseweb.clm.utexas.edu/software
