# Supplementary material for: Prolonged intermittent theta burst stimulation enhances hippocampal plasticity via GluN2A-mediated signaling
Source: Front Aging Neurosci. 2026 Mar 9;18:1757554. doi: 10.3389/fnagi.2026.1757554 (PMC13006673; doi:10.3389/fnagi.2026.1757554)
Supplement: Supplementary file 1 [file Supplementary_file_1.doc]

**Fig. S1**

**Fig. S1** Changes in the weight of the animals during the seven-day stimulation period

1


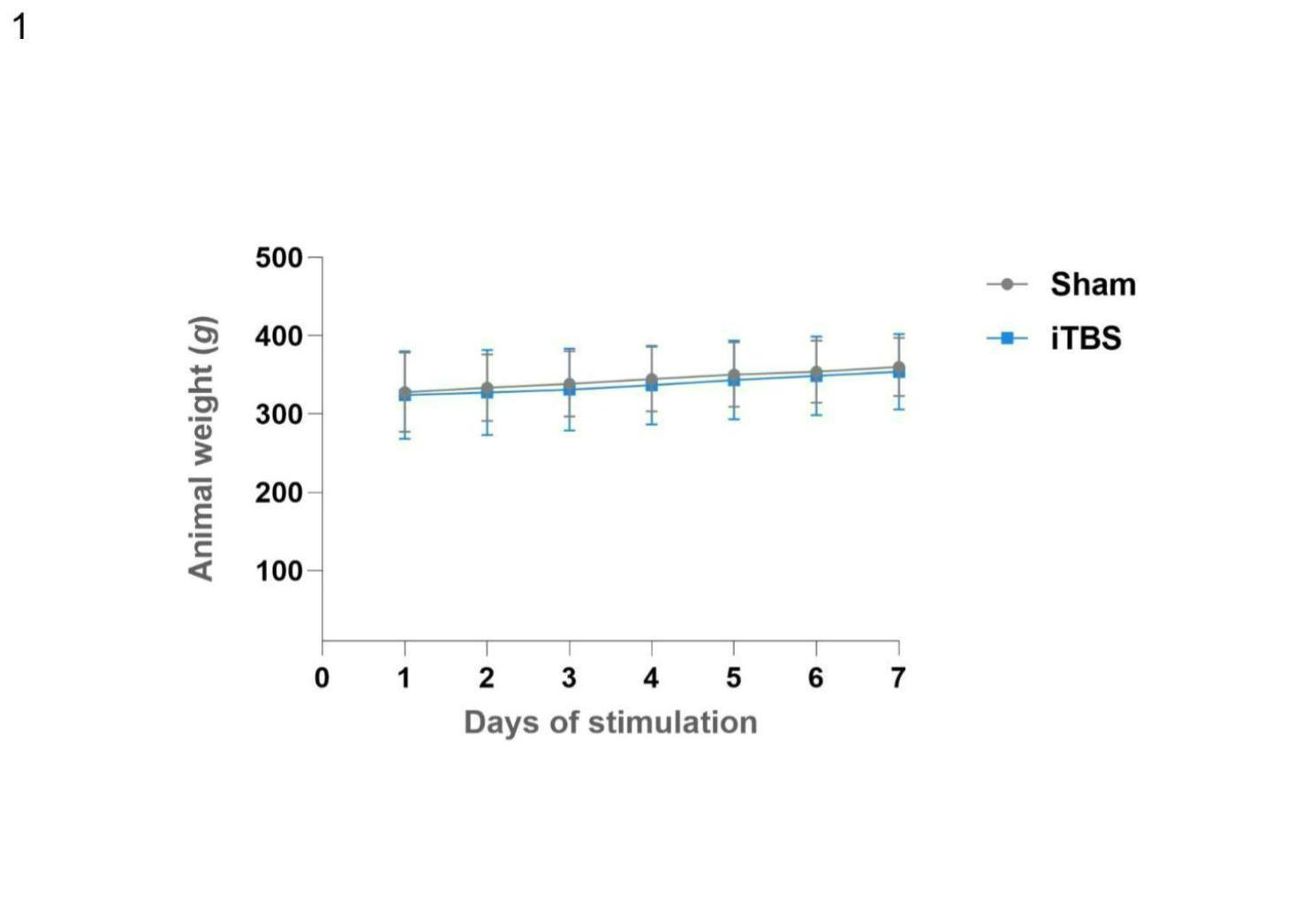


**Fig. S2**

**Fig. S2 Correlation graphs showing the number of entries into the central zone correlated**

**with the time spent there for each group (A, C) before and (B, D) after the stimulation period.**

Correlations were tested using Pearson’s correlation test. The Pearson correlation coefficient (r) is

shown on each graph. Dots in the graphs represent the values of individual animals (n=19 animals

per group)

2


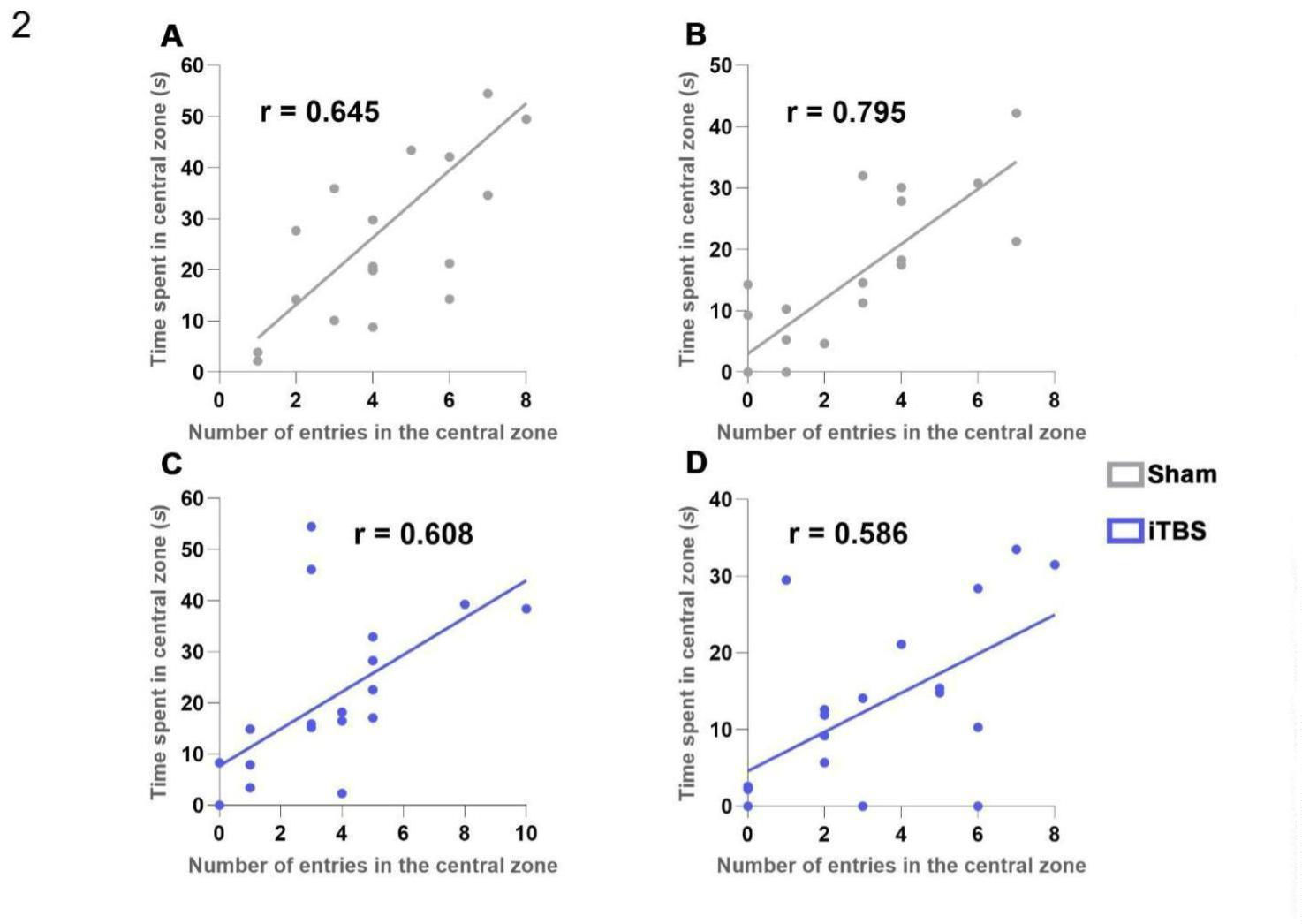


**Fig. S3**

**Fig. S3 Immunohistochemical staining and western blot analysis of c-Fos, a marker of**

**neuronal activation, in the hippocampus.** (A) Representative micrographs of c-Fos

3


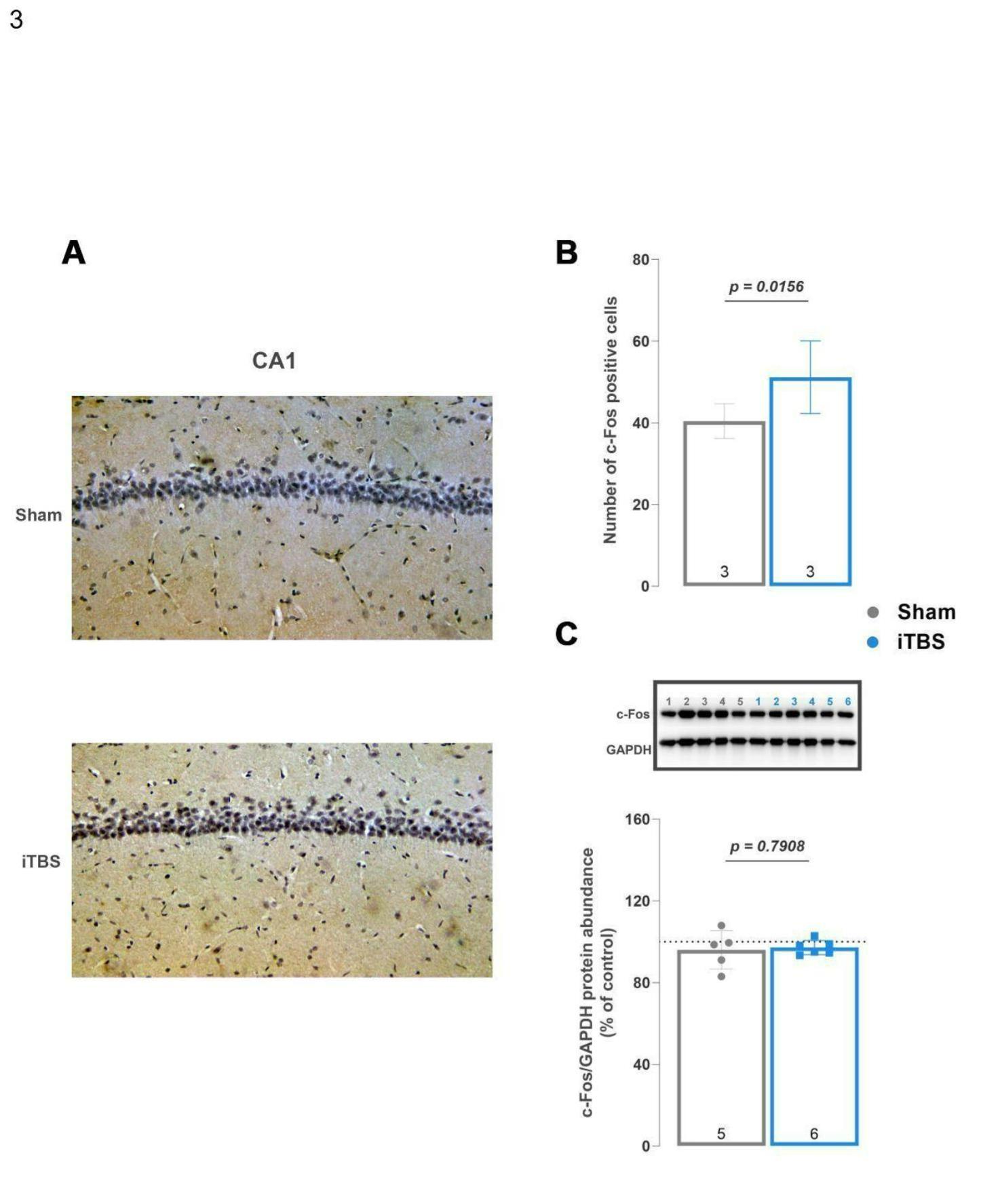


immunostaining in the CA1 subfield, with (B) graph showing the number of c-Fos-positive cells.

Numbers at the bottom of the graphs show the number of animals included in the analysis (n=3

animals per group). Obtained results were analyzed using two-tailed Student’s t-test. Data are

expressed as mean ± SD. Significance is shown on graphs as p-value, in bold for p < 0.05. (C)

Graph and representative membranes of c-Fos with representative bands for GAPDH. Numbers

above the membranes show the sample layout. c-Fos protein expression is presented as the

percentage of control. Dots in the graph represent the values of individual animals. Numbers at

the bottom of the graph show the number of animals included in the analysis (n=5-6 animals per

group). Obtained results were analyzed using two-tailed Student’s t-test. Data are expressed as

arbitrary units derived from optical density ± SD. Significance is shown on the graph as p-value,

in bold for p < 0.05

4

**Fig. S4**

**Fig. S4 Evidence for synaptosomal activity and purity of isolated synaptosomal fraction.**

(A) Representative calcium dynamics graph showing activity of isolated synaptosomes during

calcium imaging. Representative micrographs show the fluorescent images of a single

synaptosome in time points t0 and t1 as indicated on the graph. (B) The purity of isolated

hippocampal synaptosomal fraction was demonstrated with western blot analysis of expression

of presynaptic (synaptophysin), postsynaptic (PSD-95) and cytosolic (PV) protein markers, along

with the representative bands for GAPDH as endogenous control

5


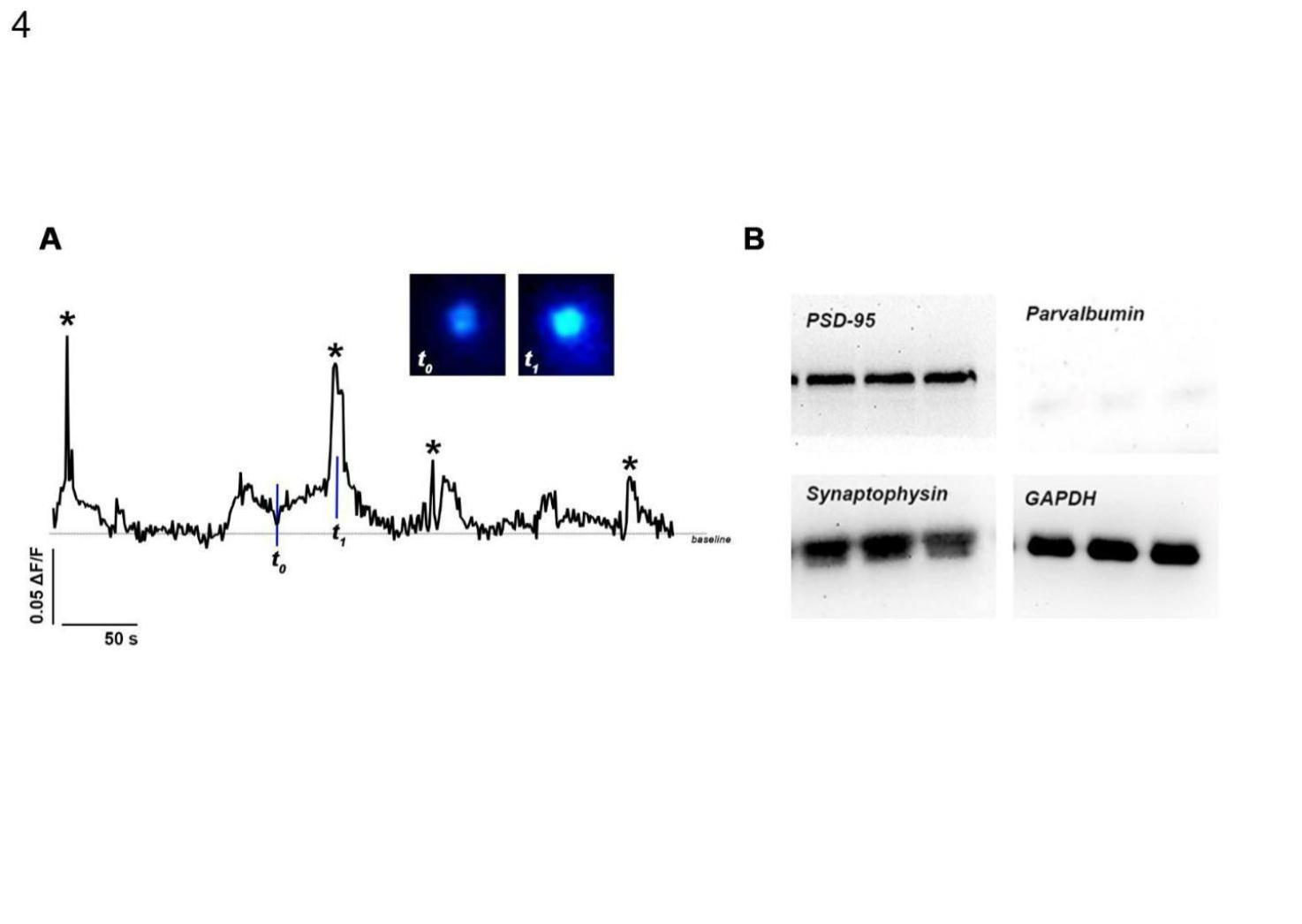


**Fig. S5**

**Fig. S5 Western blot analysis of synaptophysin, protein marker of presynapse, in three different timepoints following prolonged iTBS600 in hippocampal synaptosomal fraction, including graphs and representative membrane with its respective GAPDH.** Numbers above the membranes show the sample layout. Dots in the graphs represent the values of individual animals. Numbers at the bottom of the graphs show the number of animals included in the analysis (n=3 animals per group). Obtained results were analyzed using Kruskal-Wallis test followed by Dunnett’s *post hoc* analysis. Data are expressed as arbitrary units derived from optical density ± SD. Significance is shown on graphs as p-value, in bold for *p* < 0.05.

6


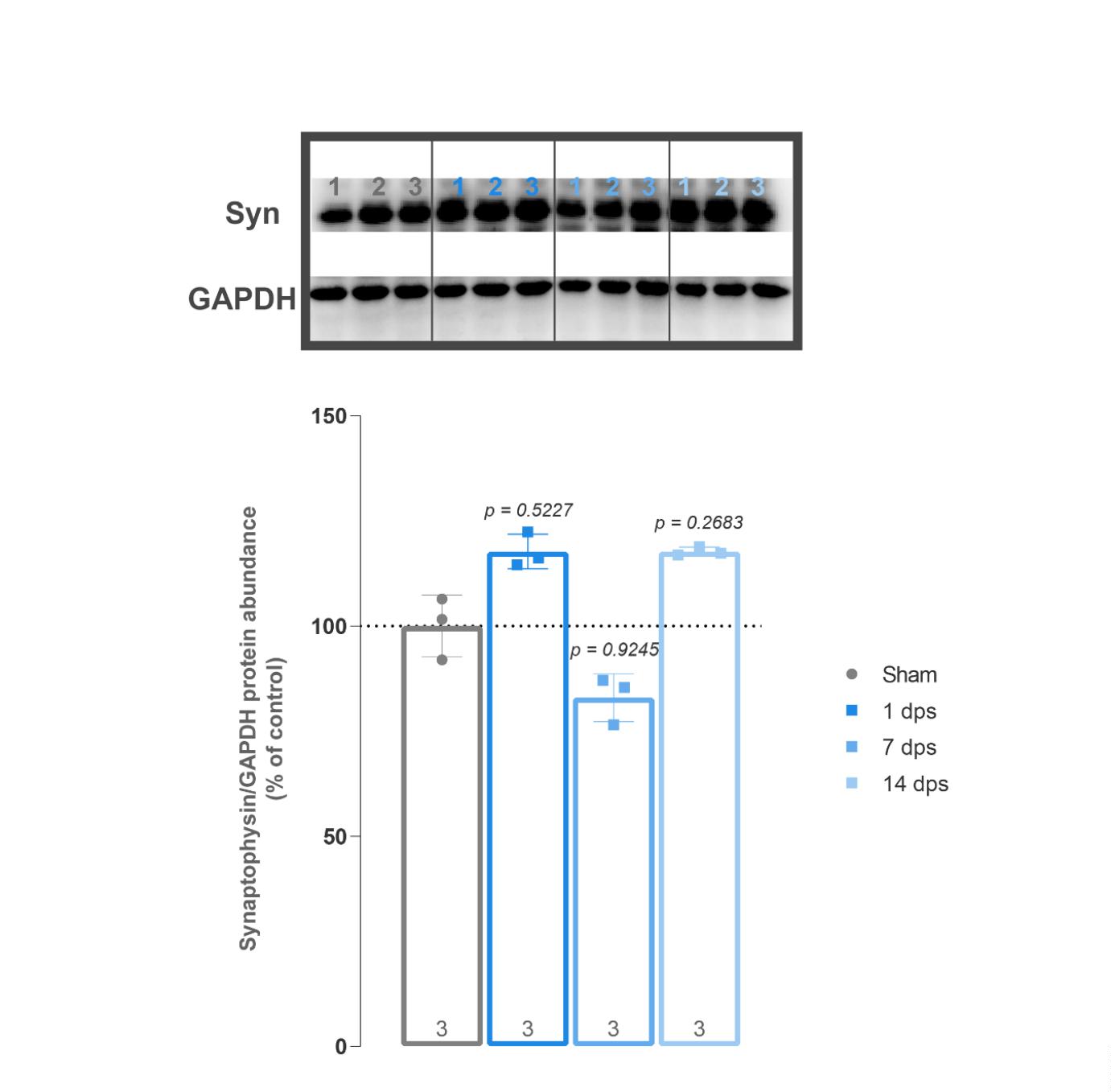

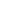


5

**Fig. S6**

**Fig. S6 Representative PCR gel used for genotyping Grin2a +/+ (wild-type, WT) and**

**Grin2a -/- (knock-out, KO) mouse pups, confirming the validity of the used animal model**

7


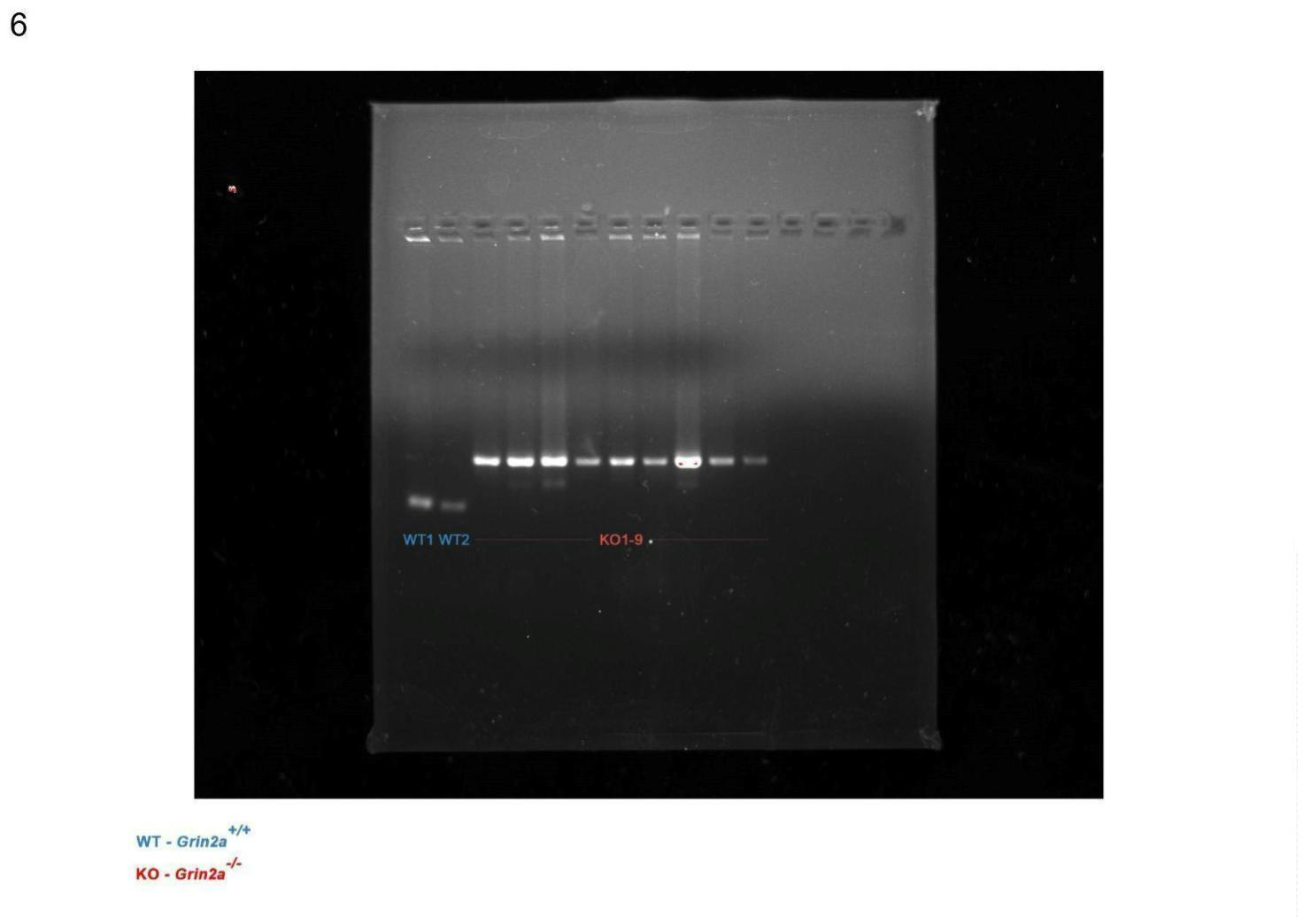


**Table S1**

Source and

type

Antibody

c-Fos

Dilution

1:400

Manufacturer

Rabbit,

polyclonal

Abcam, Cat#ab190289, RRID:

AB_2737414

Rabbit,

polyclonal

Novus, Cat#NB120-11427,

RRID_AB791498

Parvalbumin

1:300

Anti-mouse HRP-

conjugated IgG

Goat,

polyclonal

Abcam, Cat#ab97240,

RRID:AB_10695944

1:100

1:300

1:200

Anti-rabbit IgG Alexa Fluor Donkey,

555-conjugated

Molecular Probes, Cat#A-31572,

RRID:AB_162543

polyclonal

Alexa Fluor 488-conjugated

streptavidin

/

Thermo Fisher Scientific, Cat#S11223

**Table S1 List of antibodies used for immunohistochemistry and immunofluorescence**

**staining**

8


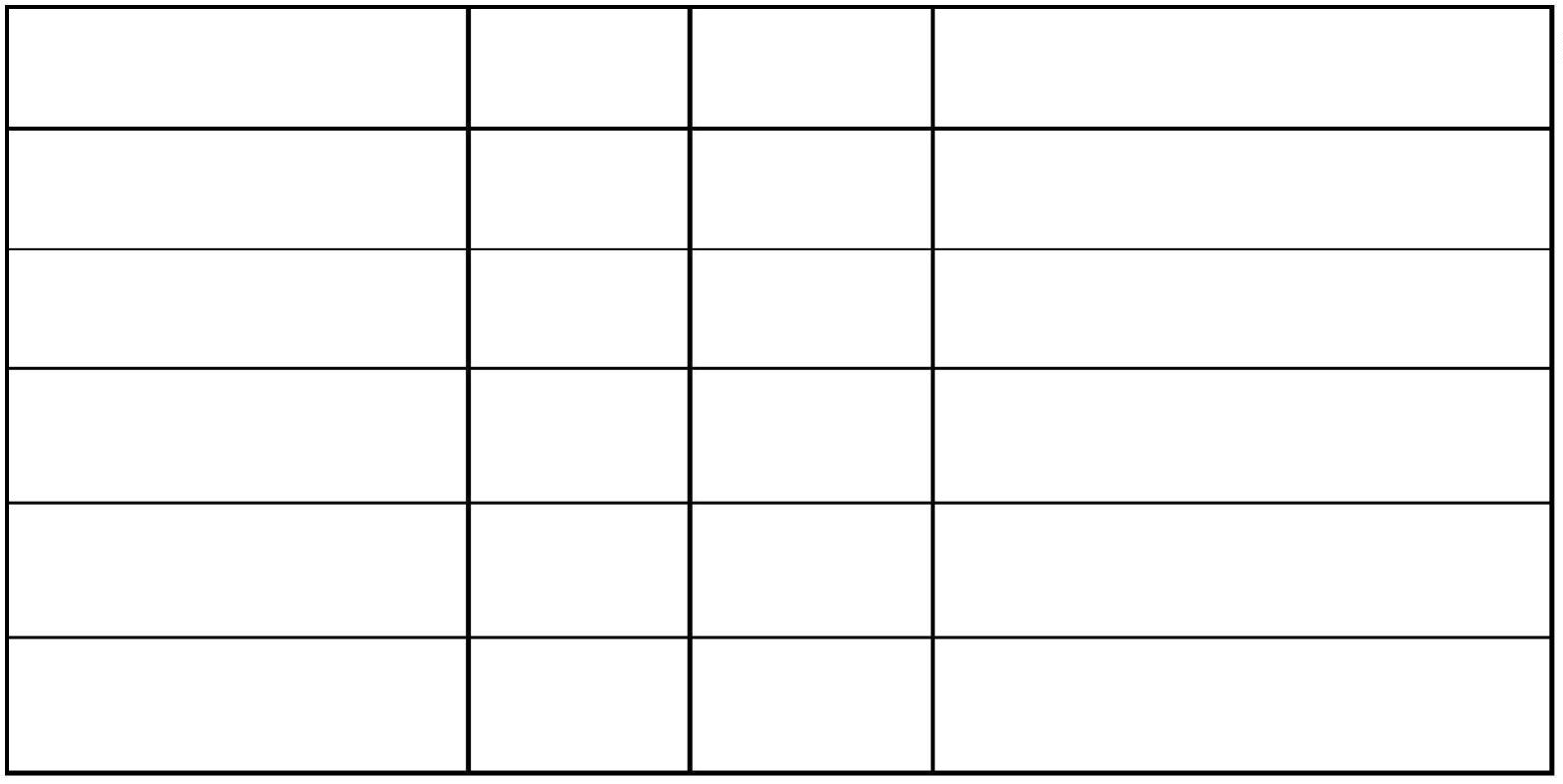


**Table S2**

Source and

type

Antibody

Dilution

1:5000

1:1000

1:2500

1:4000

1:3000

1:1000

1:5000

1:1000

1:1000

1:1000

1:1000

1:1000

Manufacturer

Rabbit,

polyclonal

Santa Cruz Biotechnology, Cat#sc-

9116, RRID:AB_2199007

Synaptophysin

PSD-95

GluN1

Mouse,

monoclonal

Millipore, Cat#MAB1598,

RRID:AB_94278

Rabbit,

monoclonal

Cell Signaling Technology, Cat#5704,

RRID:AB_1904067

Rabbit,

polyclonal

Millipore, Cat#07-632,

RRID:AB_310837

GluN2A

GluN2B

GluR1

Mouse,

monoclonal

Abcam, Cat#ab93610,

RRID:AB_10561972

Mouse,

monoclonal

Santa Cruz Biotechnology, Cat#sc-

55509, RRID:AB_629532

Guinea pig,

polyclonal

Synaptic Systems, Cat#135304, RRID:

AB_887878

VGLUT1

p-Akt

Rabbit,

polyclonal

Cell Signaling Technology, Cat#9271,

RRID:AB_329825

Rabbit,

polyclonal

Cell Signaling Technology, Cat#9272,

RRID:AB_329827

t-Akt

Rabbit,

monoclonal

Cell Signaling Technology, Cat#5536,

RRID:AB_10691552

p-mTOR

t-mTOR

p-ERK1/2

Rabbit,

monoclonal

Cell Signaling Technology, Cat#2983,

RRID:AB_2105622

Rabbit,

polyclonal

Cell Signaling Technology, Cat#9101,

RRID:AB_331646

9


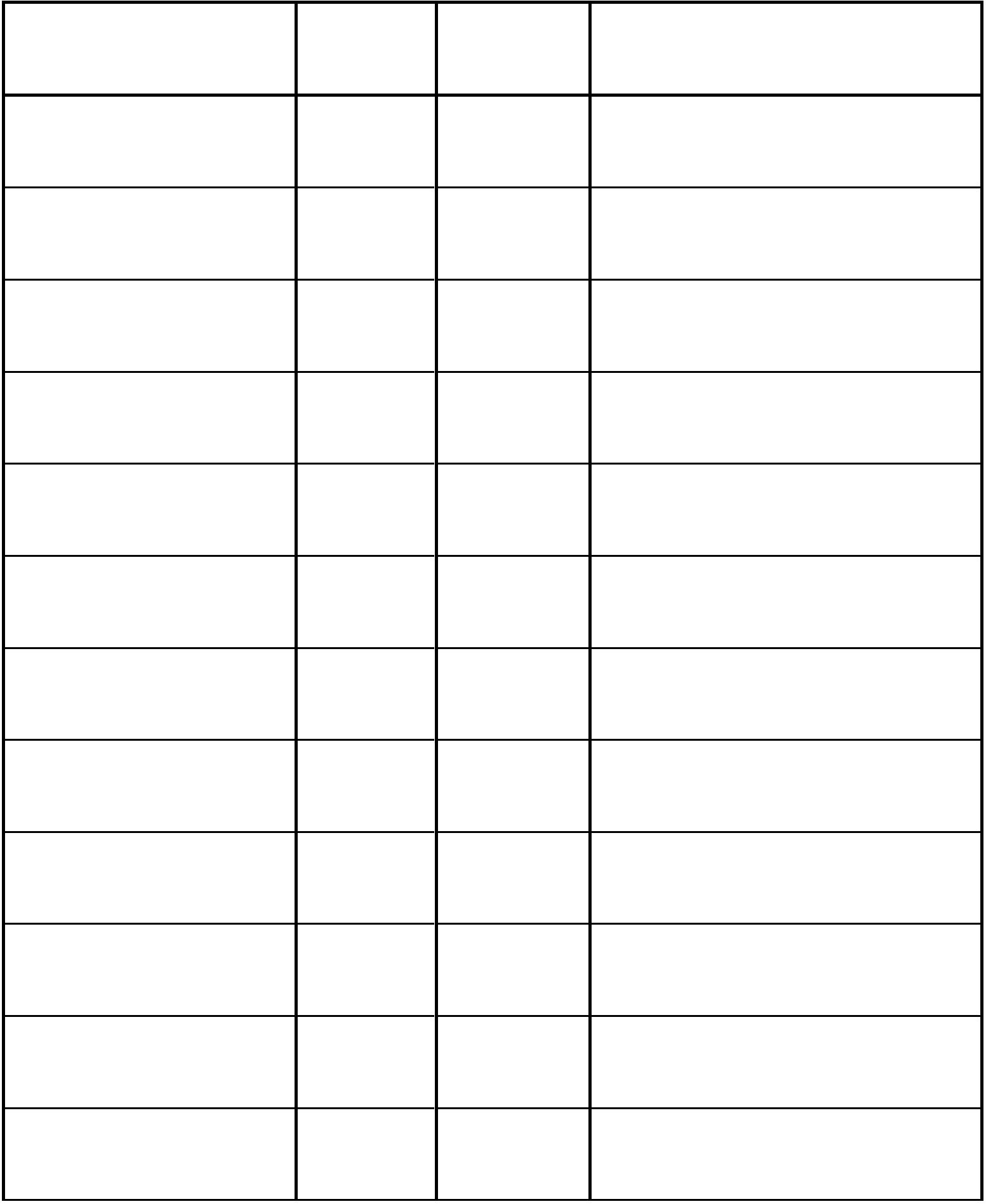


Rabbit,

polyclonal

Cell Signaling Technology, Cat#9102,

RRID:AB_330744

t-ERK1/2

EAAT1

EAAT2

BDNF

1:1000

1:300

Rabbit,

monoclonal

Cell Signaling Technology, Cat#5684,

RRID:AB_10695722

Rabbit,

polyclonal

Abcam, Cat#ab69098,

RRID:AB_2190732

1:2000

1:2000

1:300

Rabbit,

monoclonal

Abcam, Cat#ab108319,

RRID:AB_10862052

Sheep,

polyclonal

TrkB

Osenses, Cat#OST00118G-500UG

Mouse,

monoclonal

Sigma-Aldrich, Cat#P3088,

RRID:AB_477329

PV

1:2000

1:500

Rabbit,

polyclonal

c-Fos

Elabscience, Cat#E-AB-30900

Mouse,

monoclonal

Abcam, Cat#ab2723, RRID:

AB_303248

t-PSD95

p-PSD95

p-GluR1

t-GluR1

GAPDH

1:1000

1:1000

1:1000

1:1000

1:2000

Rabbit,

monoclonal

Cell Signaling Technology, Cat#45737

Rabbit,

monoclonal

Cell Signaling Technology, Cat#8084,

RRID:AB_10860773

Rabbit,

monoclonal

Cell Signaling Technology,

Cat#13185, RRID:AB_2732897

Rabbit,

polyclonal

Thermo Fisher Scientific, Cat#PA1-

987, RRID:AB_2107311

Anti-rabbit HRP-conjugated Goat,

Abcam, Cat#ab6721, RRID:

AB_955447

1:30000

1:30000

IgG

polyclonal

Anti-mouse HRP-

Goat,

Abcam, Cat#ab97240,

10


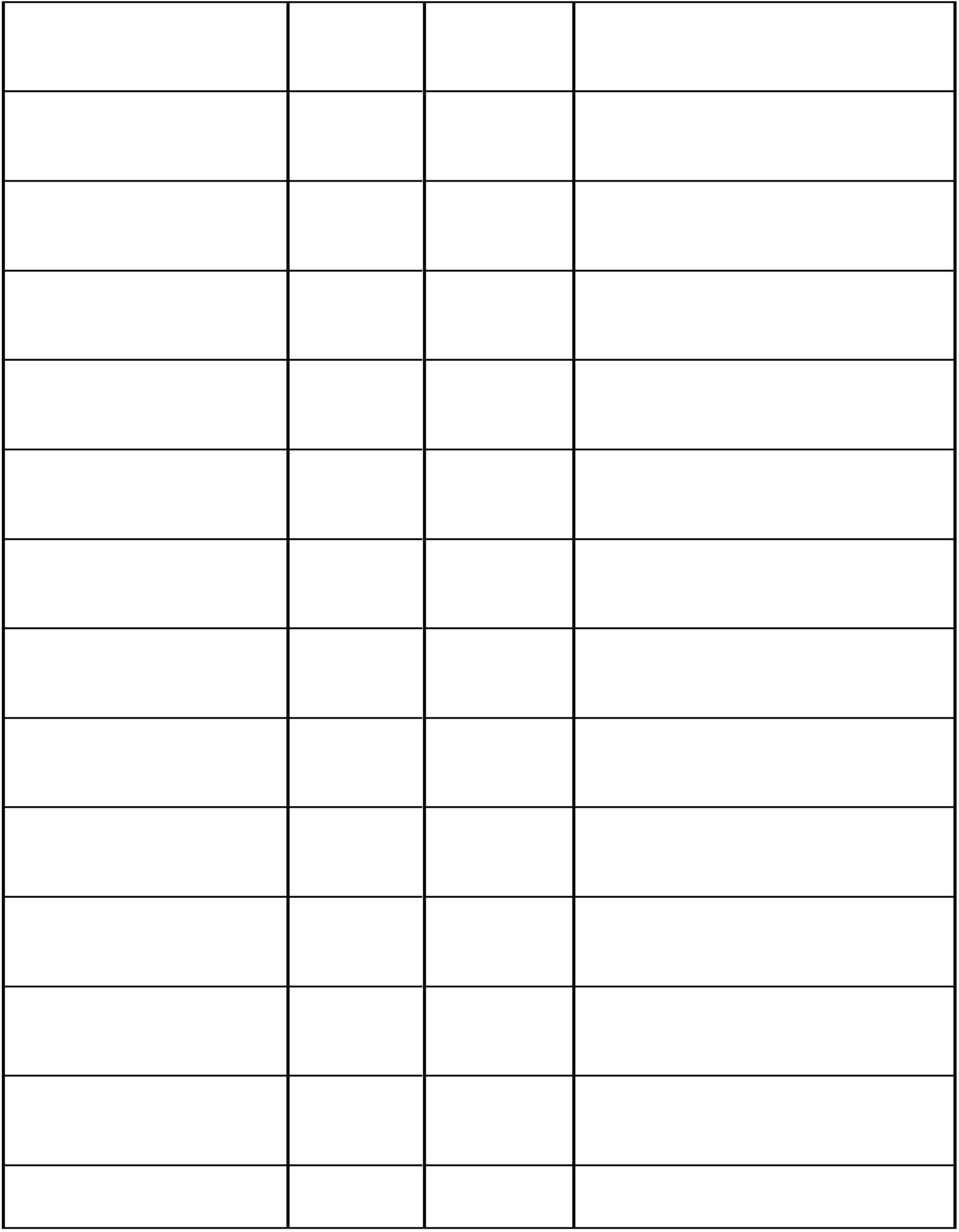


conjugated IgG

polyclonal

RRID:AB_10695944

Anti-sheep HRP-conjugated Donkey,

Abcam, Cat#ab6900,

RRID:AB_955452

1:5000

1:6000

IgG

polyclonal

Anti-guinea pig HRP-

conjugated IgG

Goat,

polyclonal

Abcam, Cat# ab6908,

RRID:AB_955425

Table S2 List of antibodies used for immunoblot

11


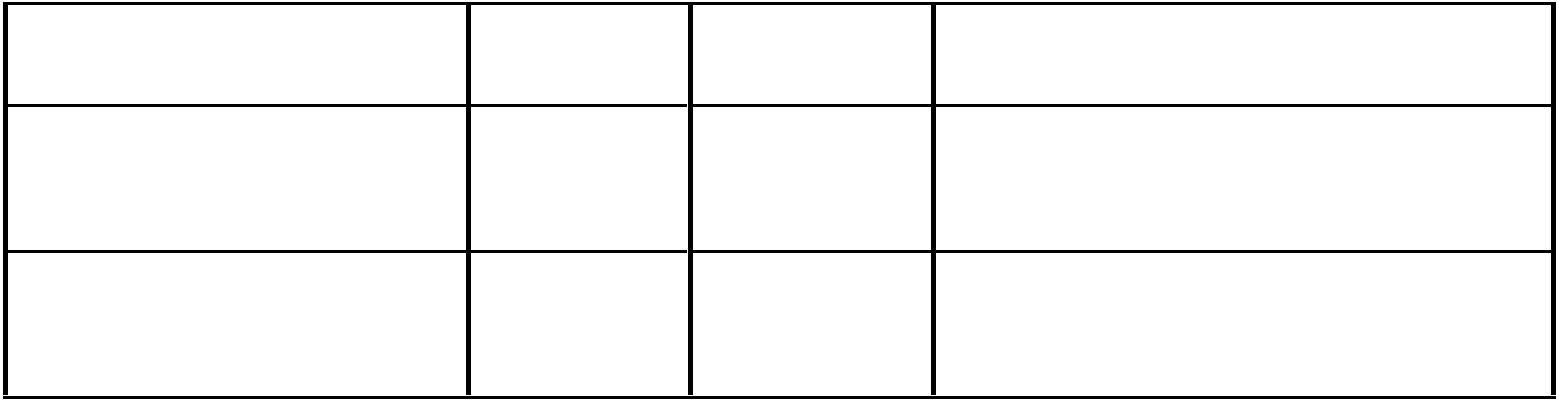


**Video S1 (separate file)**

**Video S1 Video showing an insert of one iTBS stimulation session on rats.**

12
