# Supplementary material for: Prolonged intermittent theta burst stimulation enhances hippocampal plasticity via GluN2A-mediated signaling
Source: Front Aging Neurosci. 2026 Mar 9;18:1757554. doi: 10.3389/fnagi.2026.1757554 (PMC13006673; doi:10.3389/fnagi.2026.1757554)
Supplement: Supplementary file 3 [file Data_Sheet_1.pdf]

Figure 2G

Synaptophysin

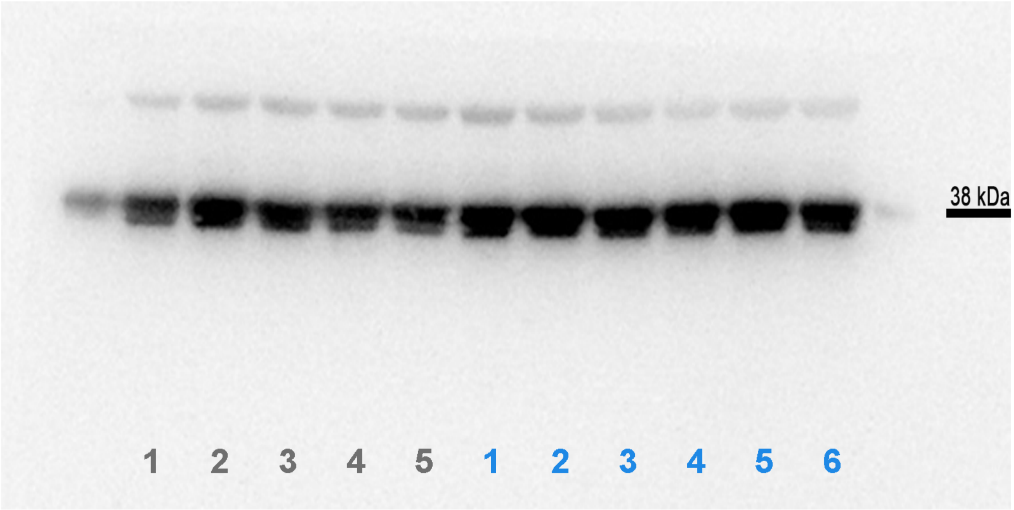

GAPDH

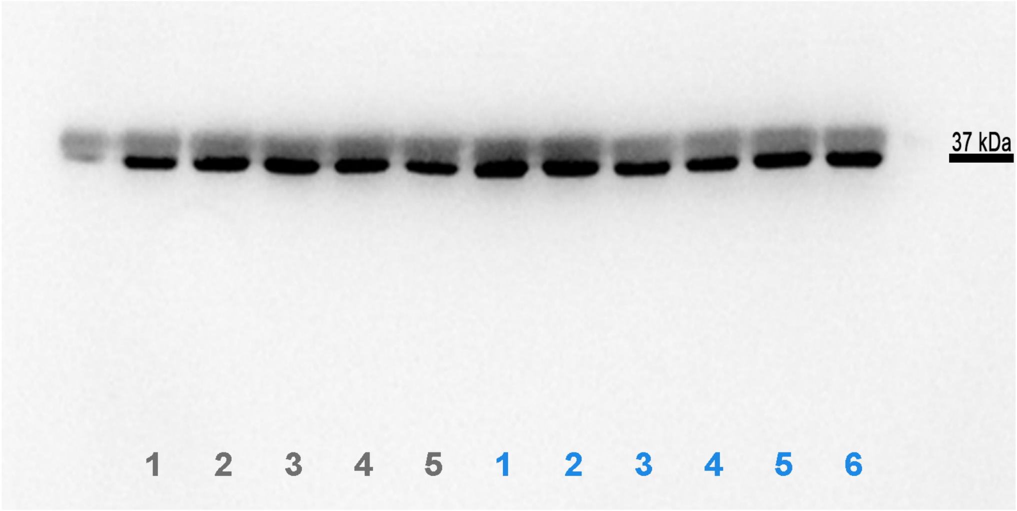

Sample order: Sham 1-5; iTBS 1-6

Figure 2H

PSD-95

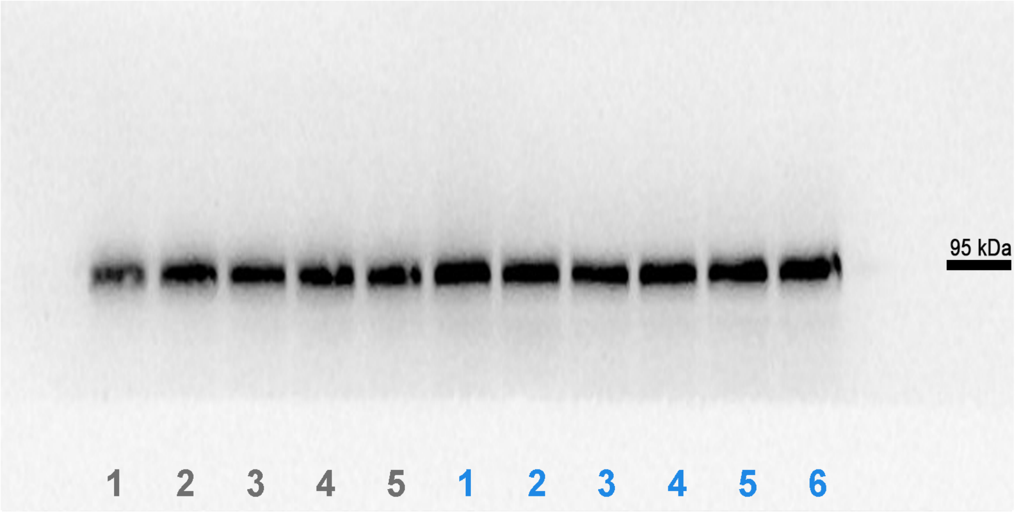

GAPDH

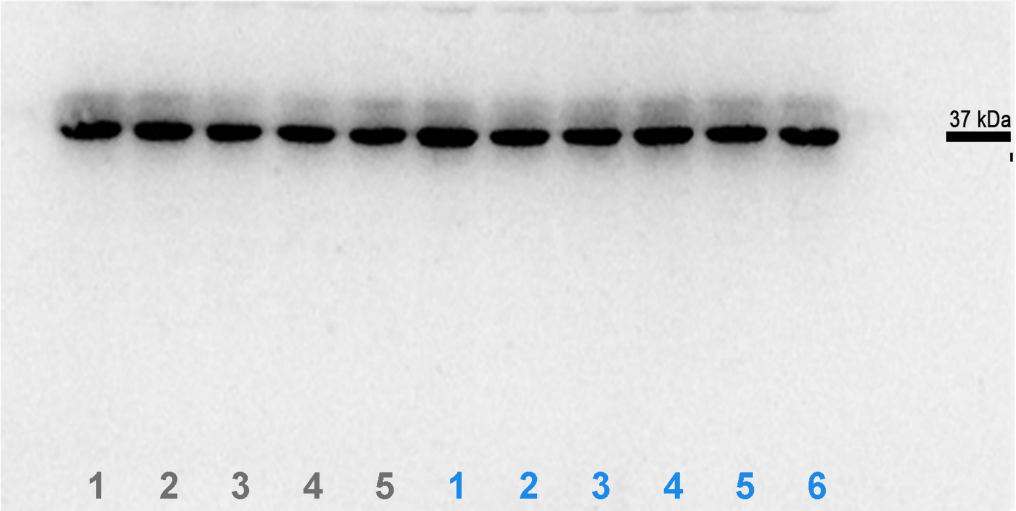

Sample order: Sham 1-5; iTBS 1-6

Figure 2I

GluR1 subunit

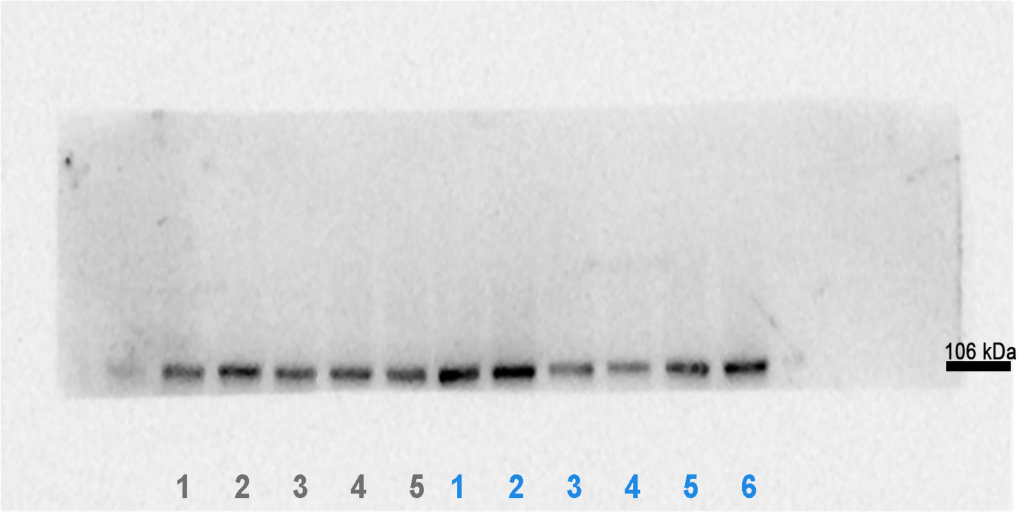

GAPDH

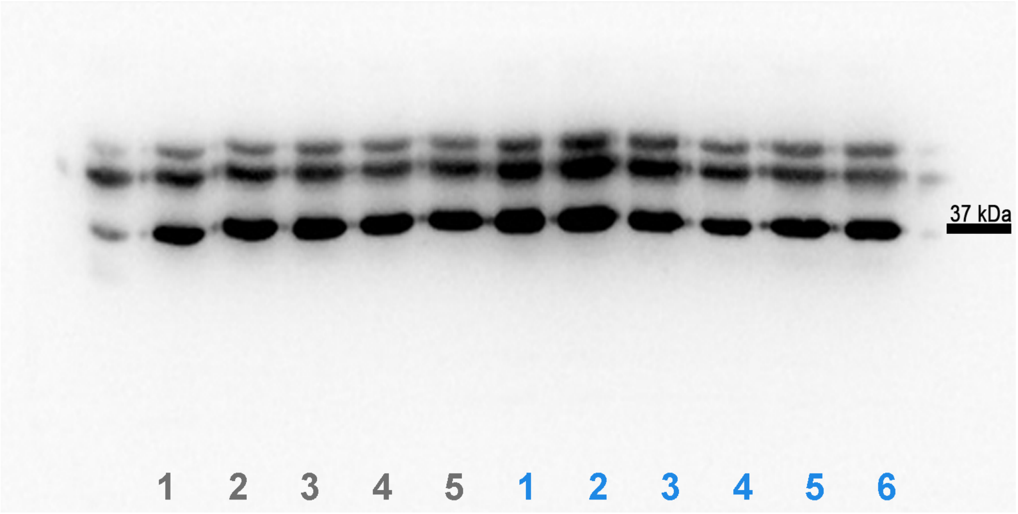

Sample order: Sham 1-5; iTBS 1-6

Figure 3A

GluN1 subunit

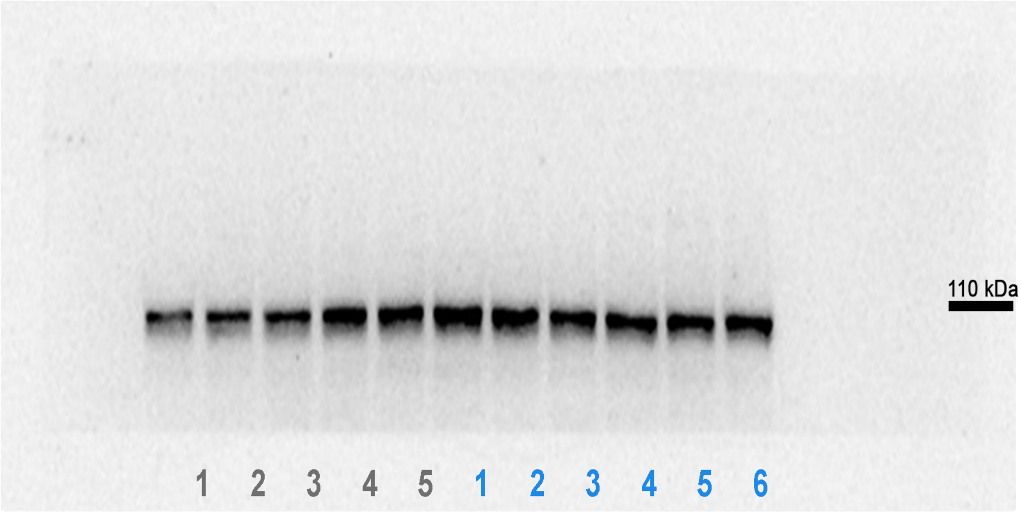

GAPDH

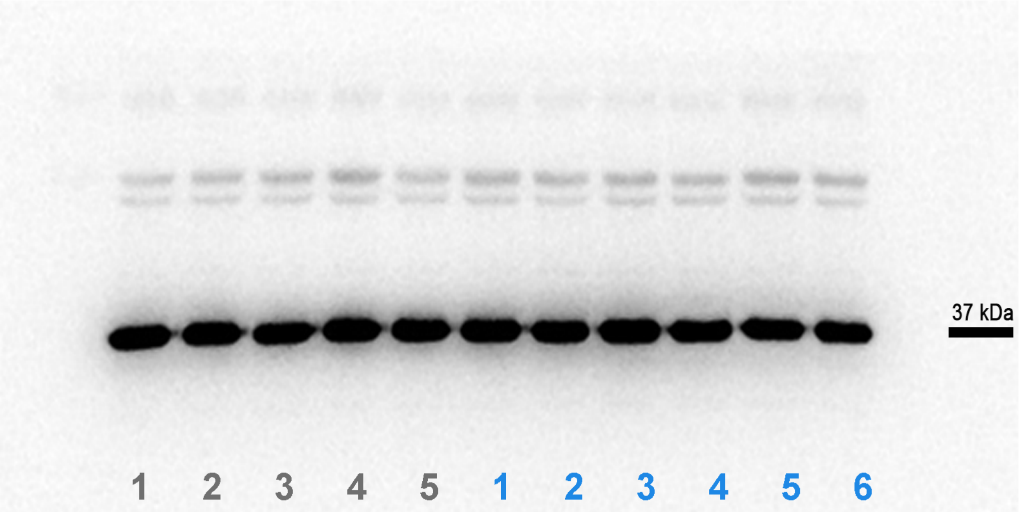

Sample order: Sham 1-5; iTBS 1-6

Figure 3B

GluN2A subunit

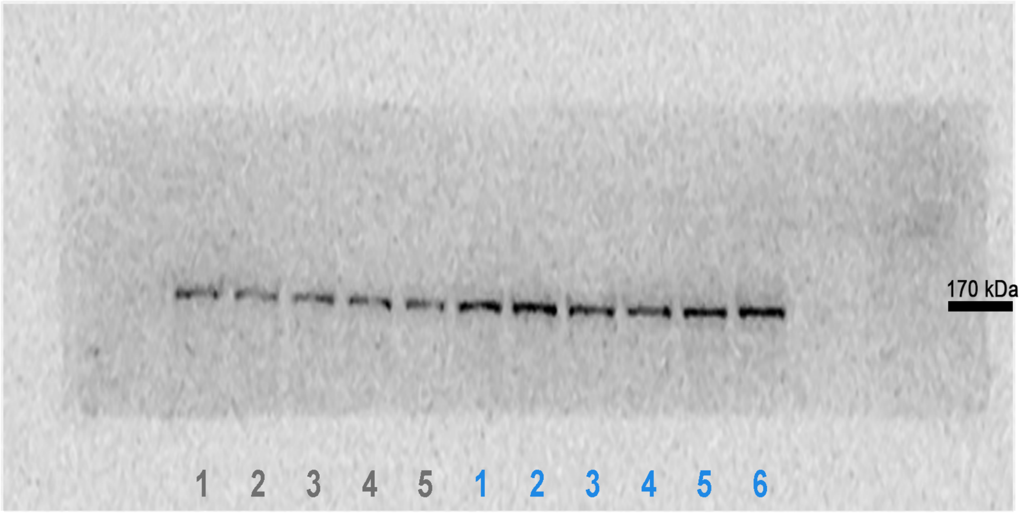

GAPDH

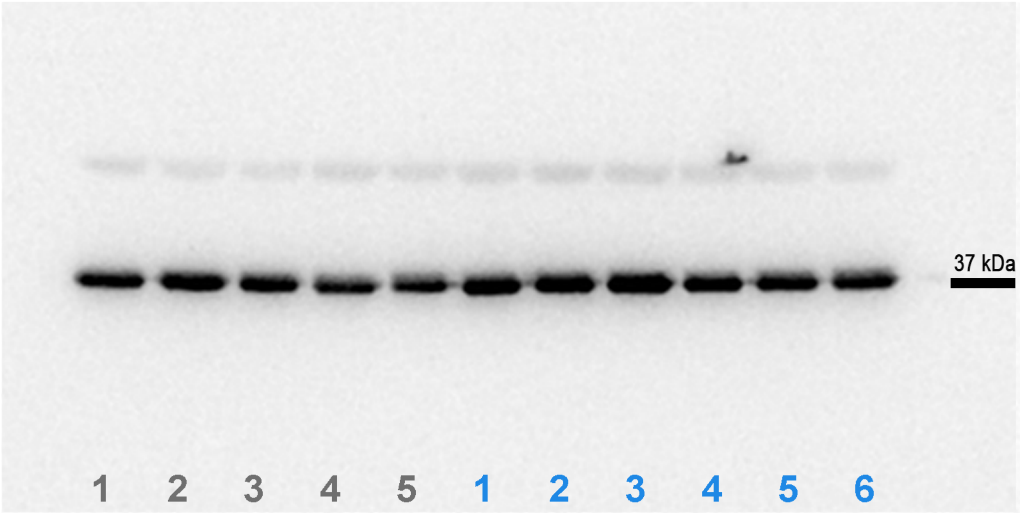

Sample order: Sham 1-5; iTBS 1-6

Figure 3C

GluN2B subunit

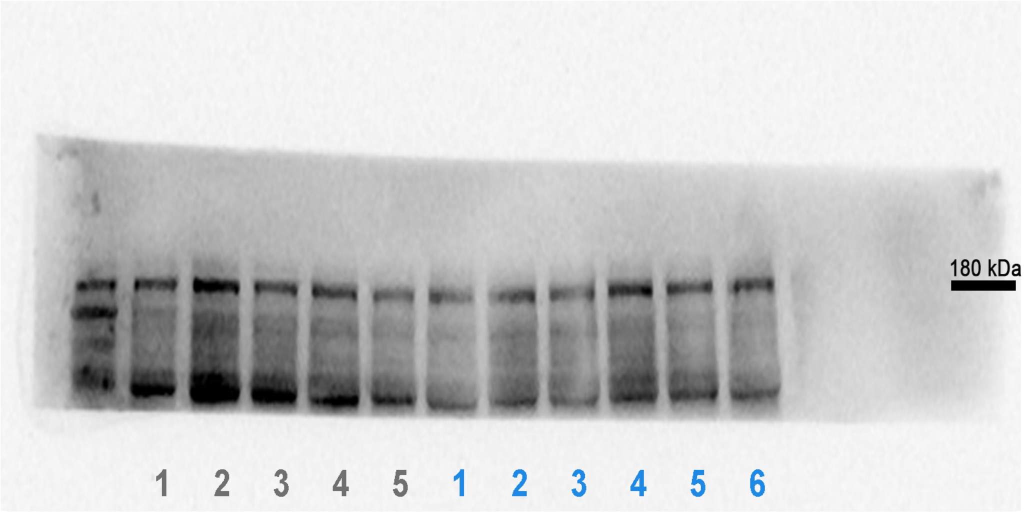

GAPDH

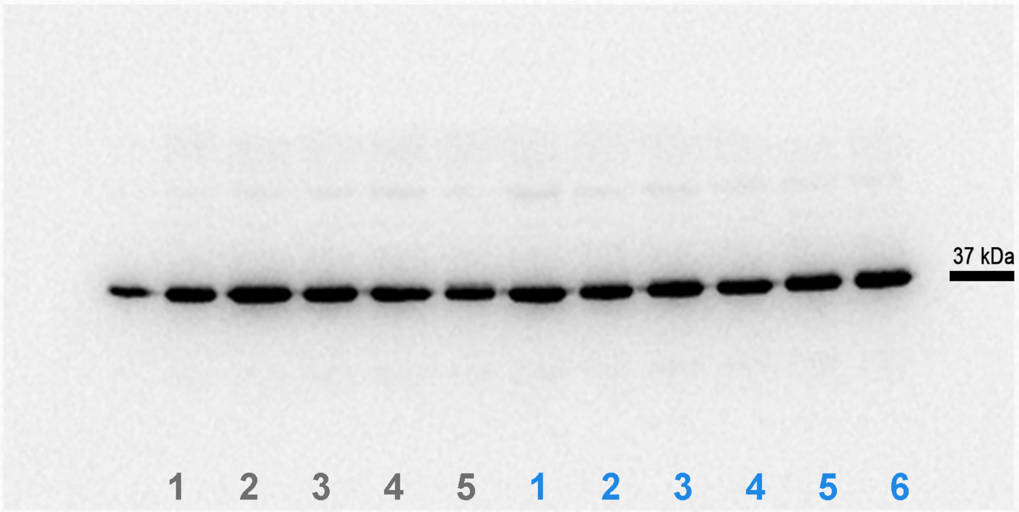

Sample order: Sham 1-5; iTBS 1-6

Figure 3D

Vesicular glutamate transporter 1 (VGLUT1)

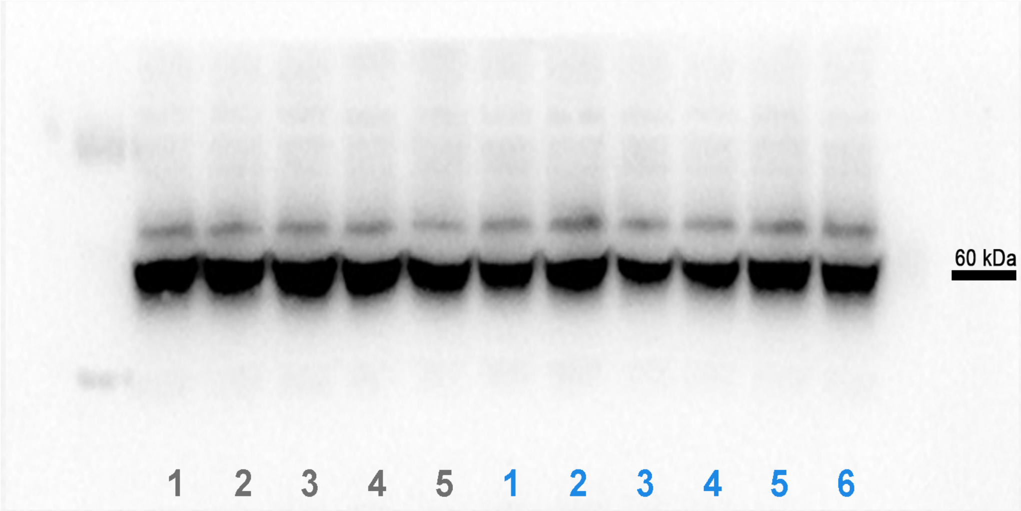

GAPDH

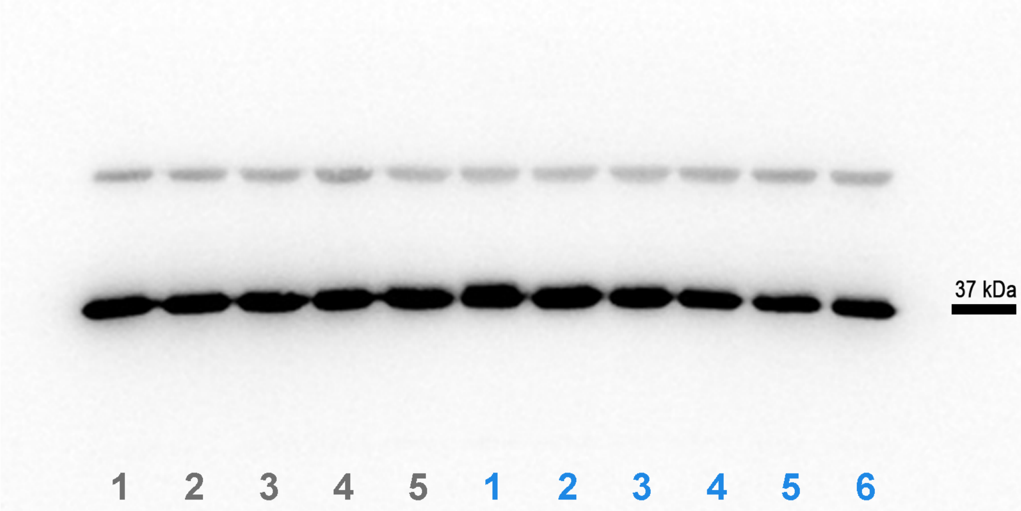

Sample order: Sham 1-5; iTBS 1-6

Figure 3E

Excitatory amino acid transporter 1 (EAAT1)

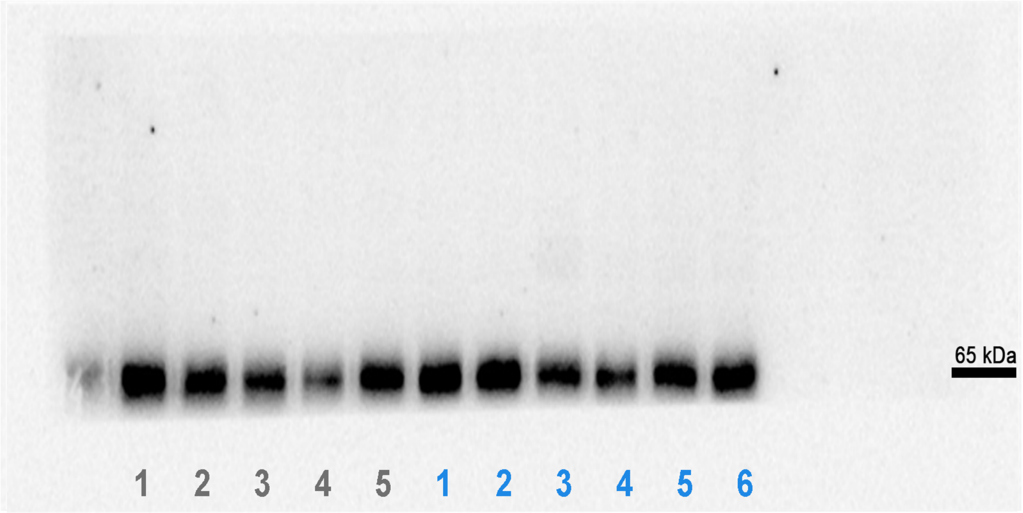

GAPDH

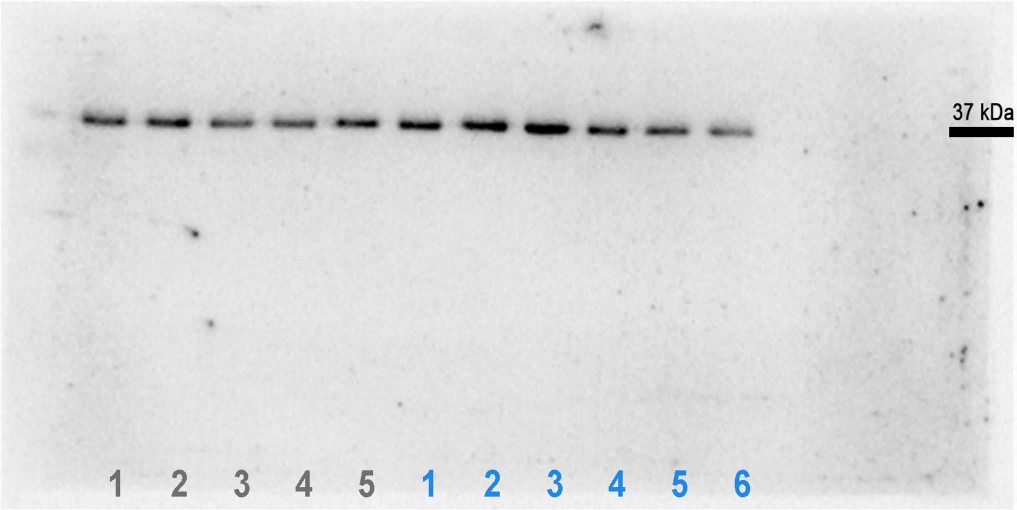

Sample order: Sham 1-5; iTBS 1-6

Figure 3F

Excitatory amino acid transporter 2 (EAAT2)

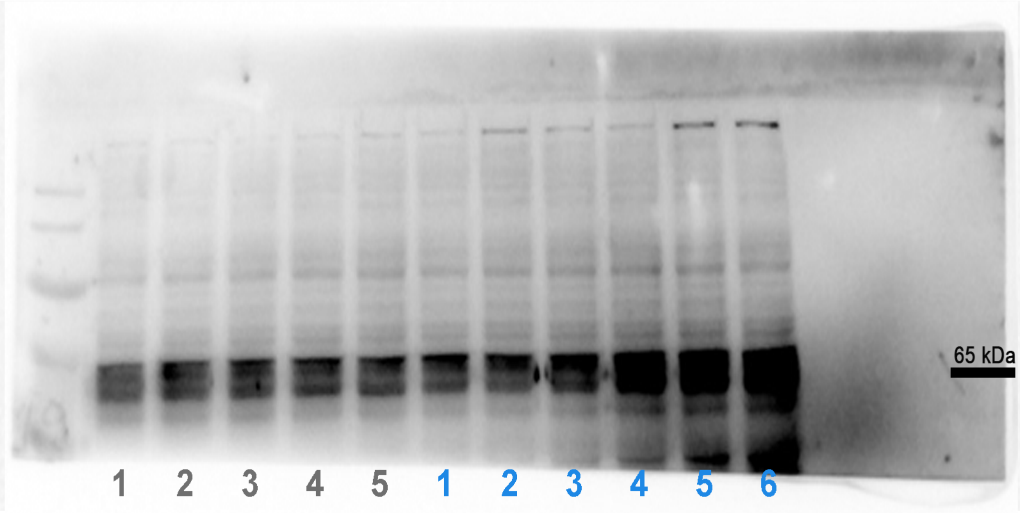

GAPDH

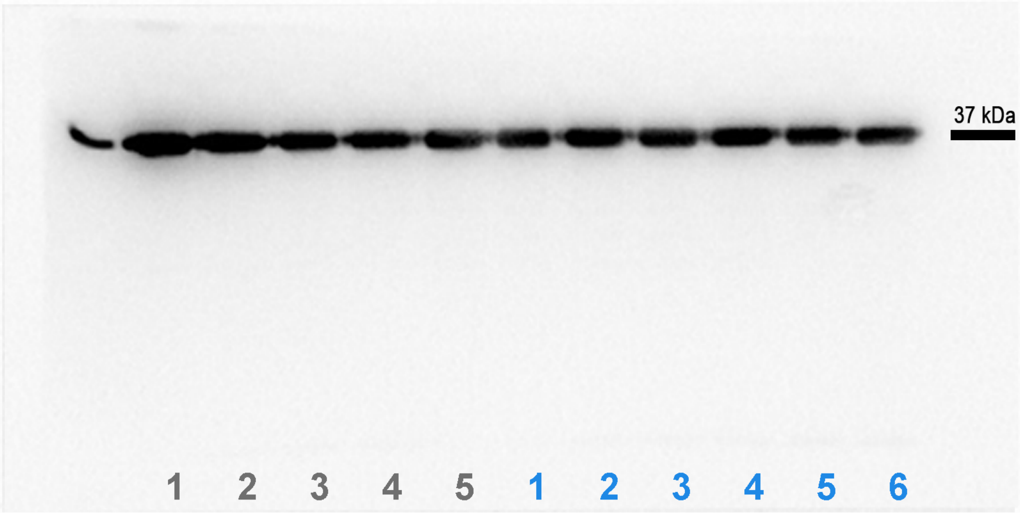

Sample order: Sham 1-5; iTBS 1-6

Figure 3G

Tropomyosin receptor kinase B (TrkB)

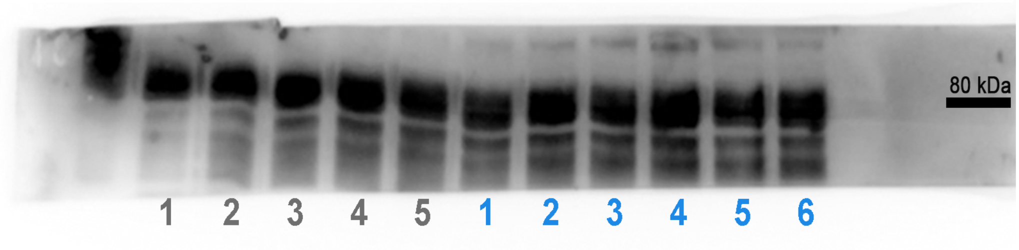

GAPDH

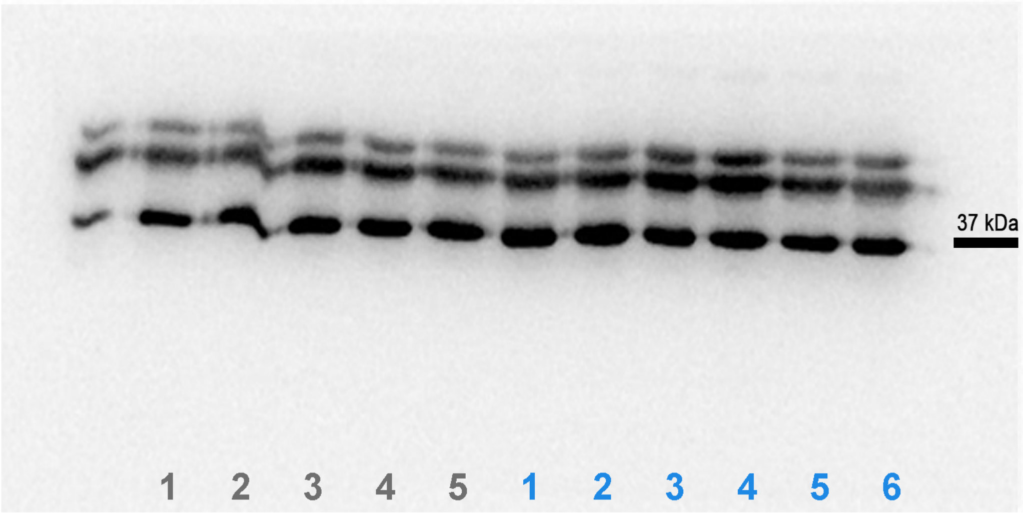

Sample order: Sham 1-5; iTBS 1-6

Figure 3H

Brain derived neurotrophic factor (BDNF)

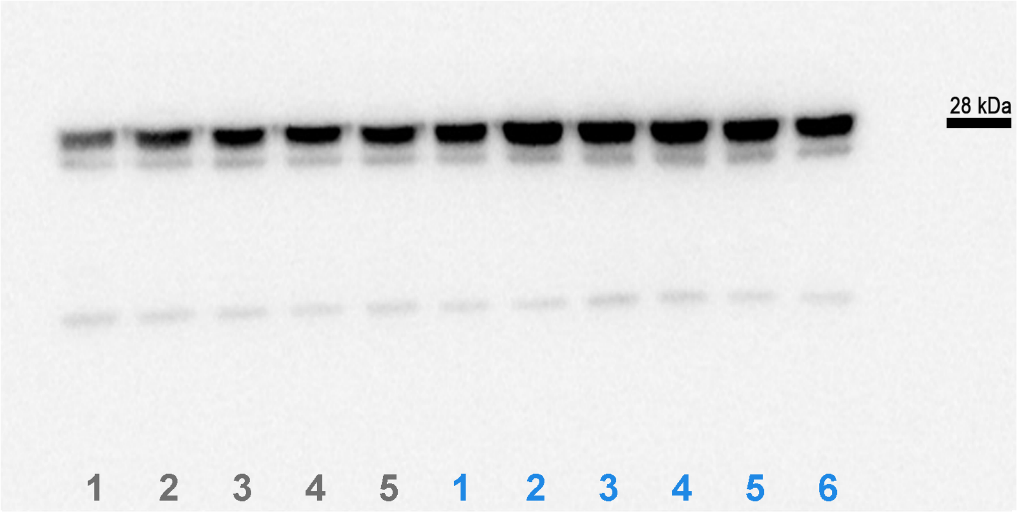

GAPDH

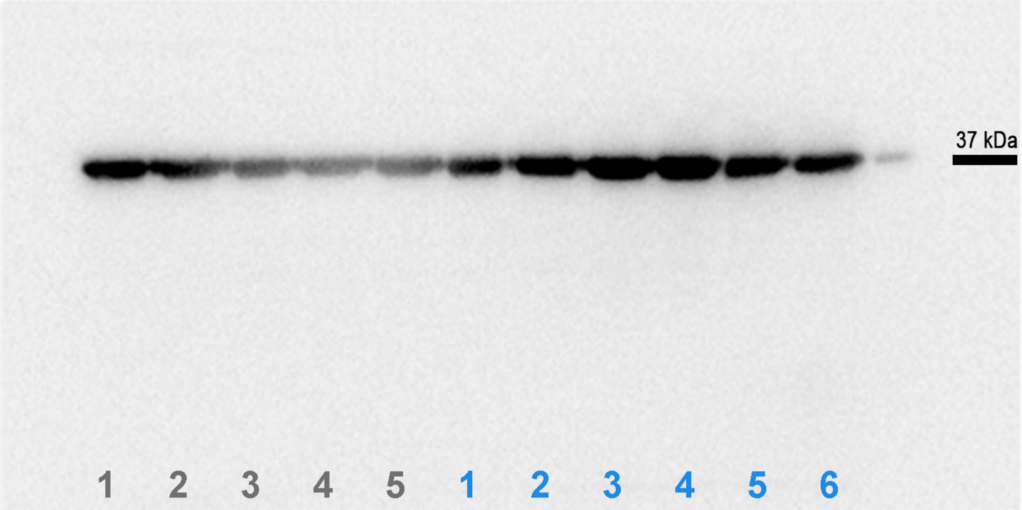

Sample order: Sham 1-5; iTBS 1-6

Figure 3I

p-protein kinase B (p-Akt)

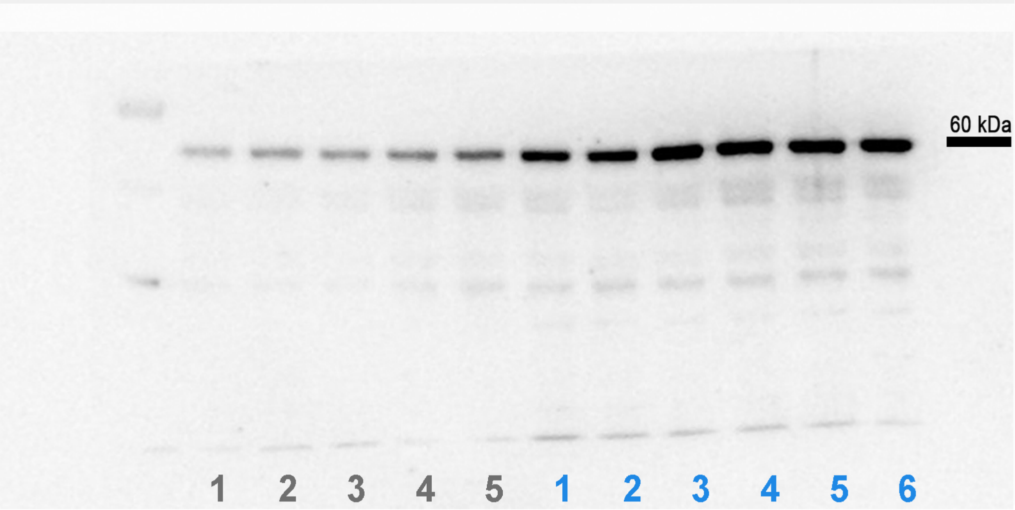

t-protein kinase B (t-Akt)

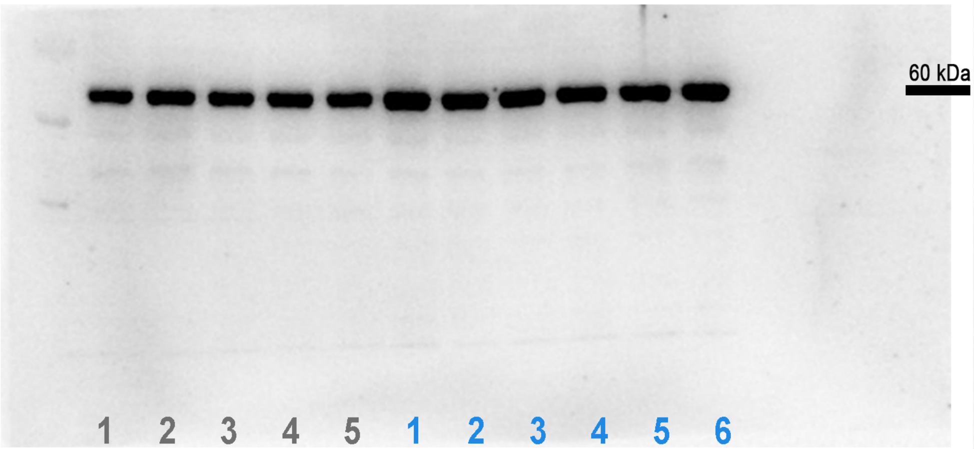

GAPDH

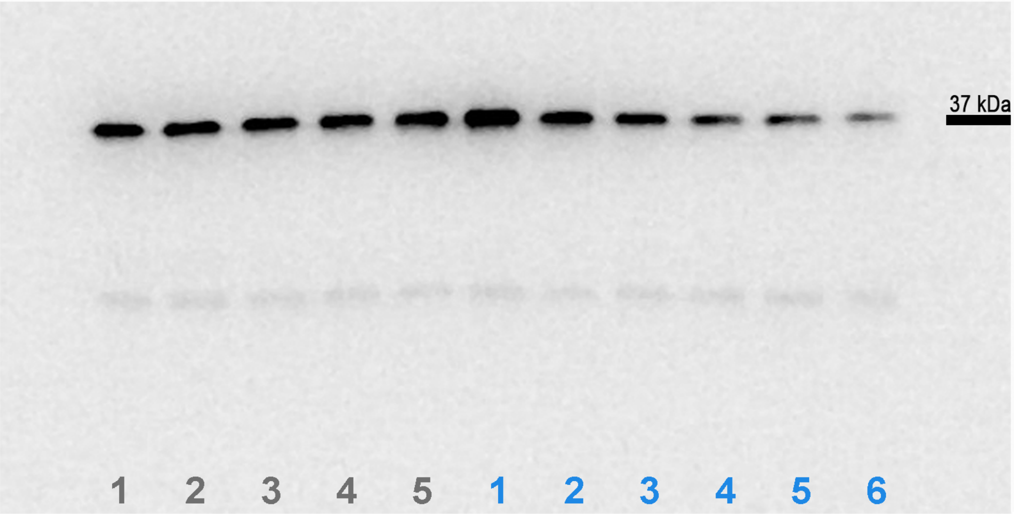

Sample order: Sham 1-5; iTBS 1-6

Figure 3J

p-extracellular signal-regulated kinase 1/2 (p-ERK1/2)

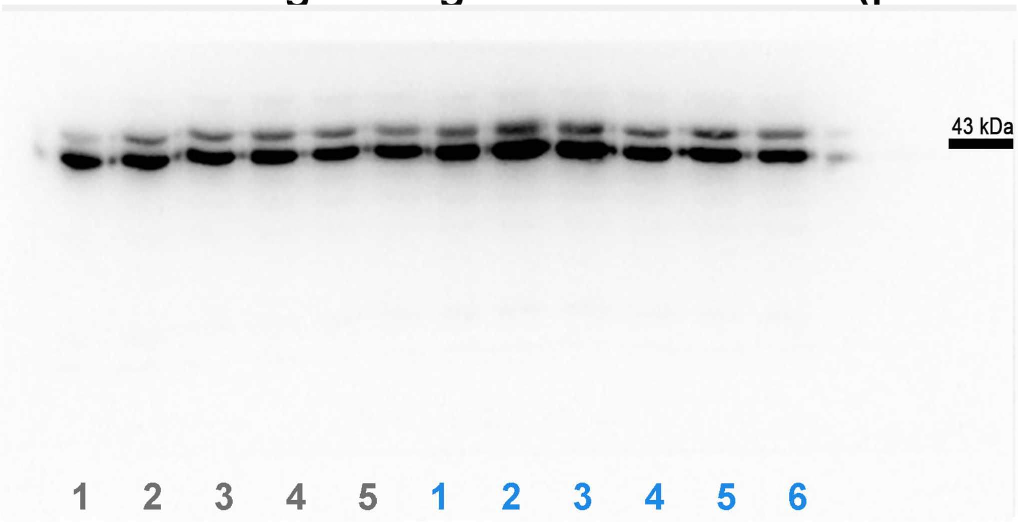

t-extracellular signal-regulated kinase 1/2 (t-ERK1/2)

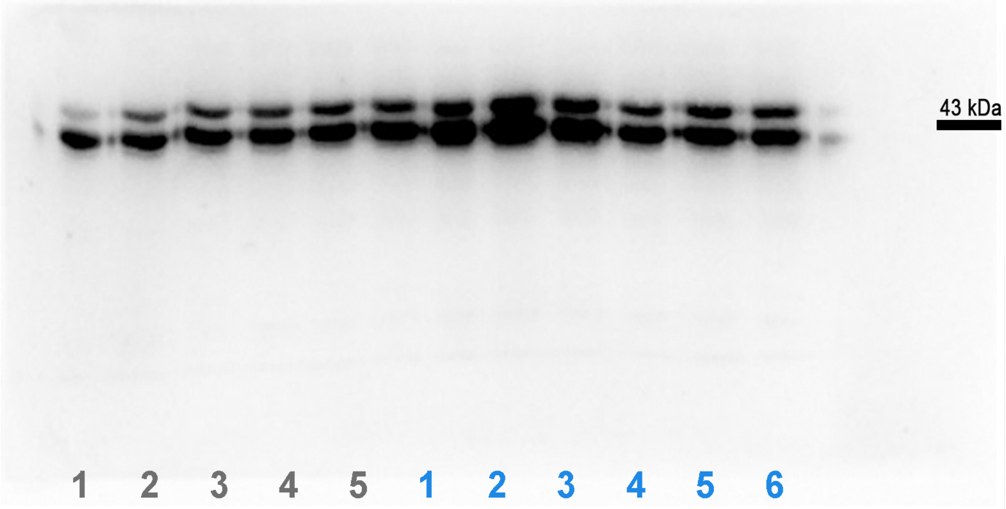

GAPDH

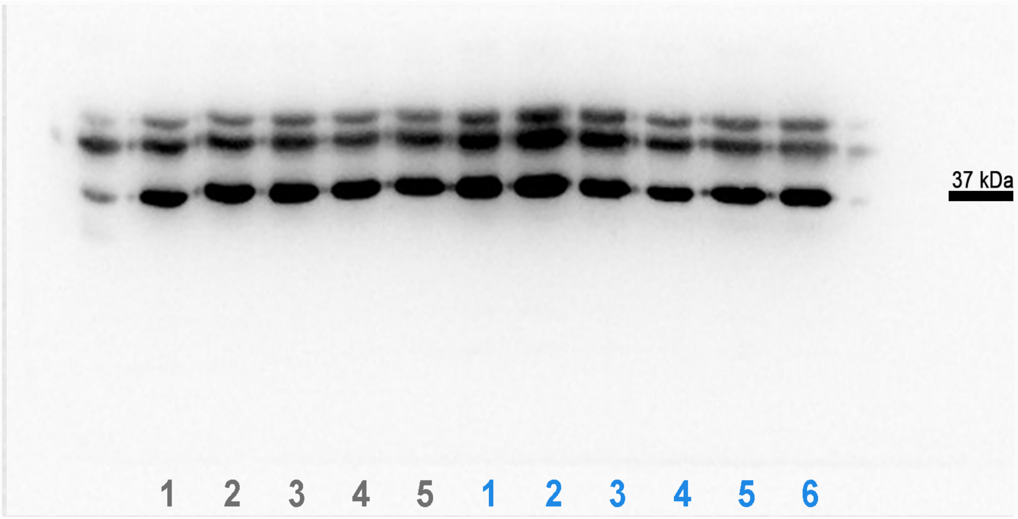

Sample order: Sham 1-5; iTBS 1-6

Figure 3K

p-mechanistic target of rapamycin (p-mTOR)

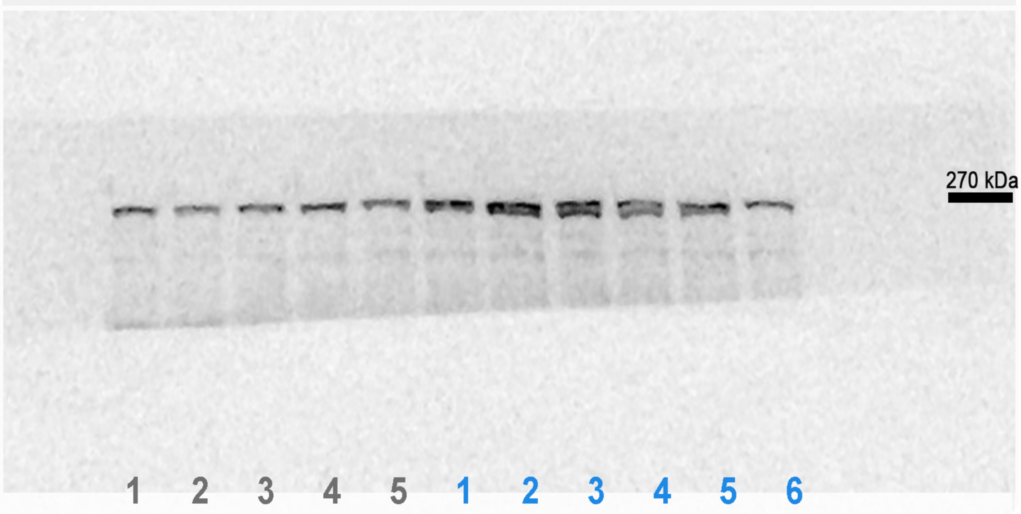

t-mechanistic target of rapamycin (t-mTOR)

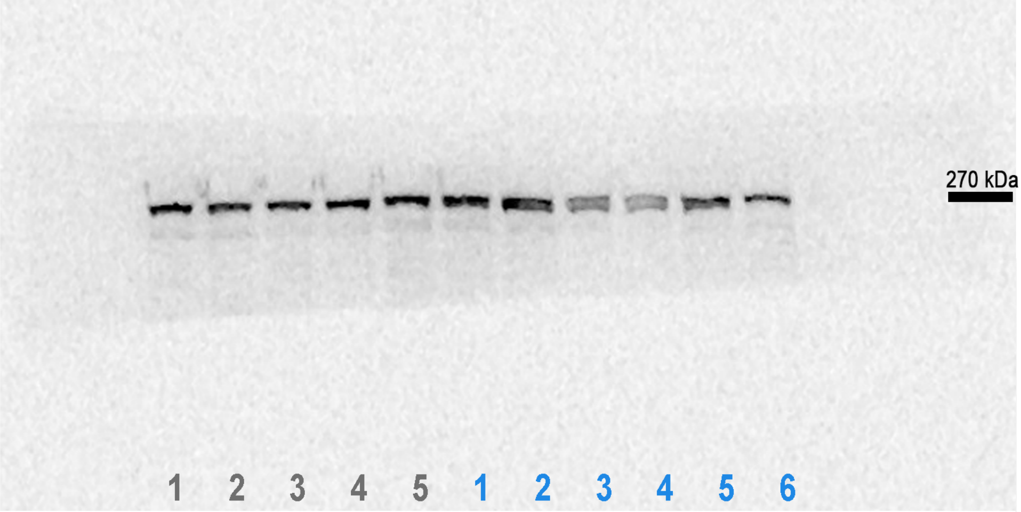

GAPDH

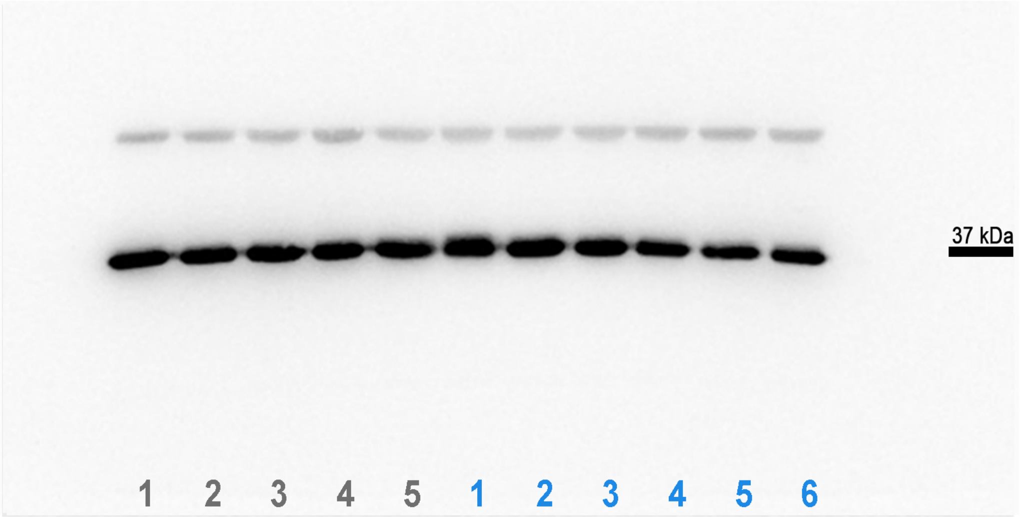

Sample order: Sham 1-5; iTBS 1-6

Figure 4H

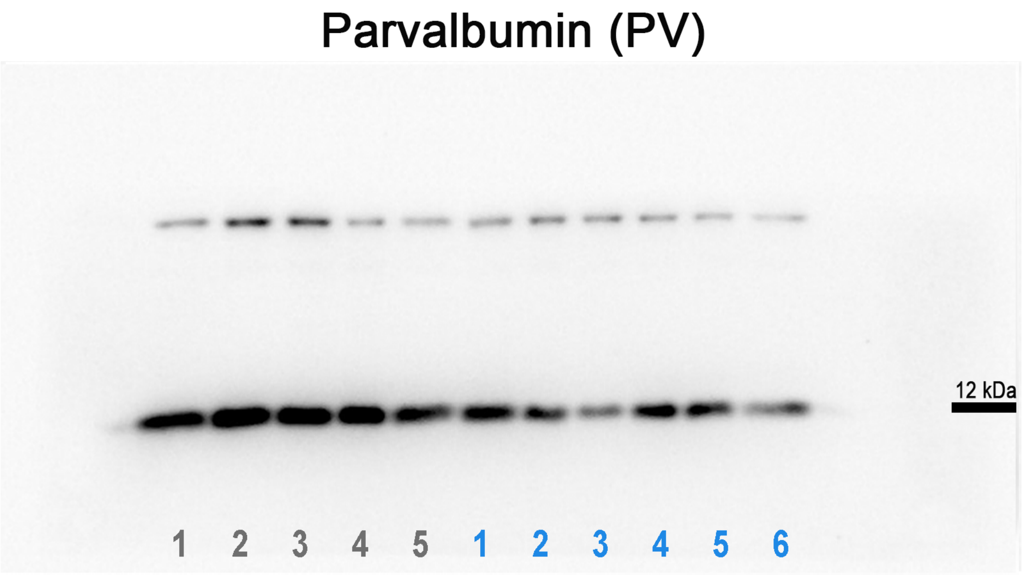

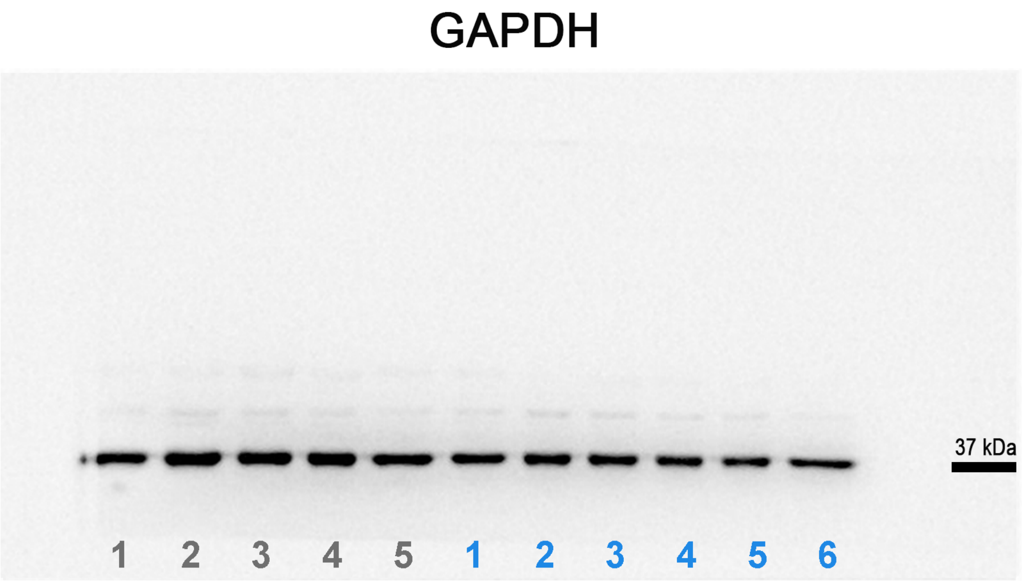

Sample order: Sham 1-5; iTBS 1-6

Figure 7D

Brain derived neurotrophic factor (BDNF)

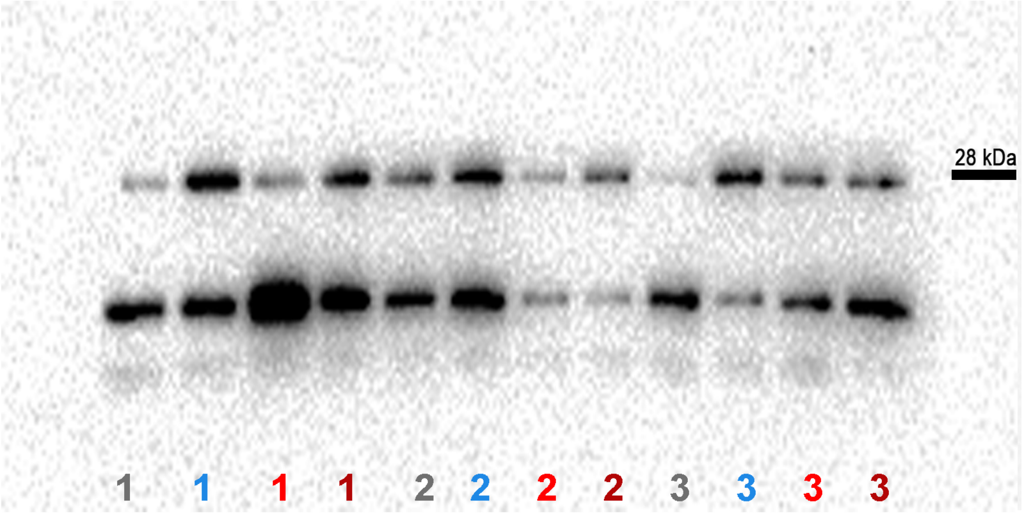

GAPDH

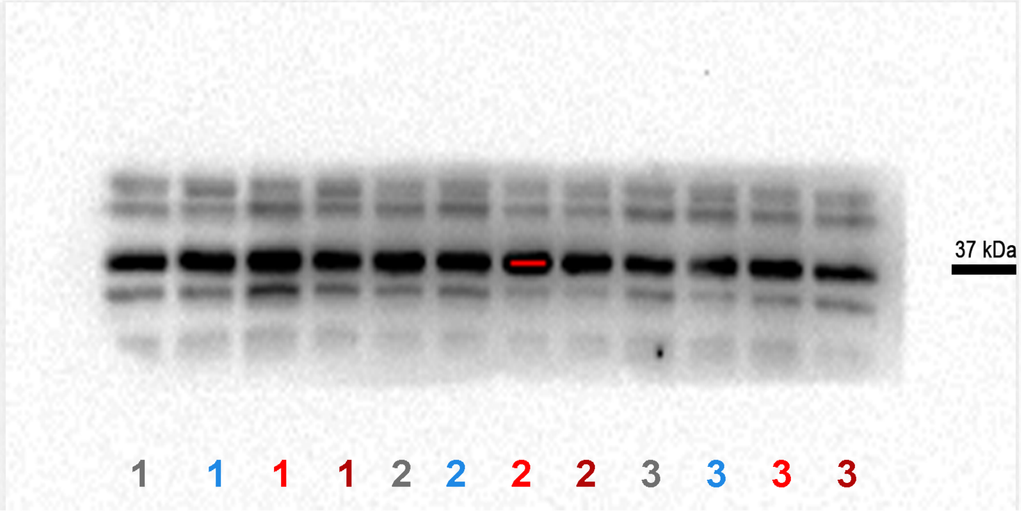

1 - 3 Sham *Grin2a*$^{+/+}$
1 - 3 iTBS *Grin2a*$^{+/+}$
1 - 3 Sham *Grin2a*$^{-/-}$
1 - 3 iTBS *Grin2a*$^{-/-}$

Figure 7D

p-extracellular signal-regulated kinase ERK1/2 (p-ERK1/2)

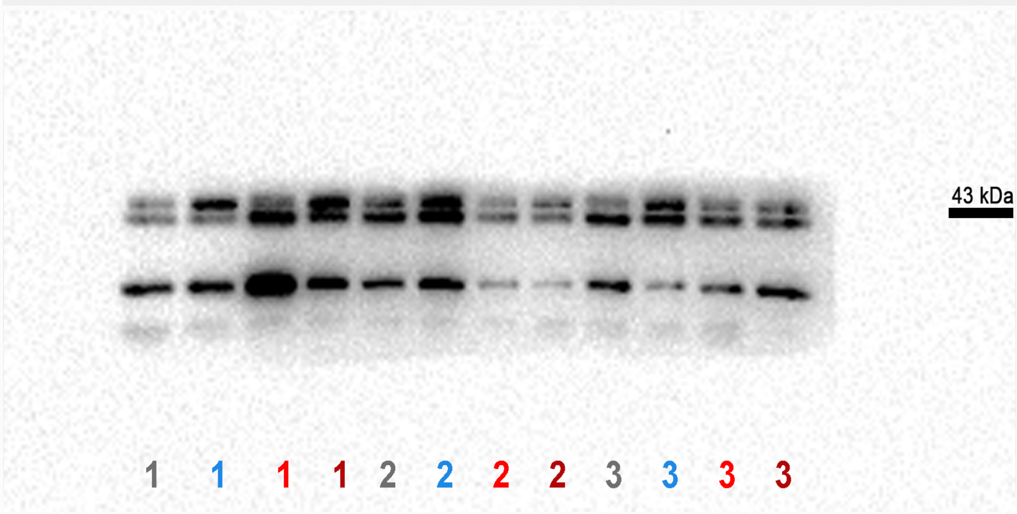

t-extracellular signal-regulated kinase ERK1/2 (t-ERK1/2)

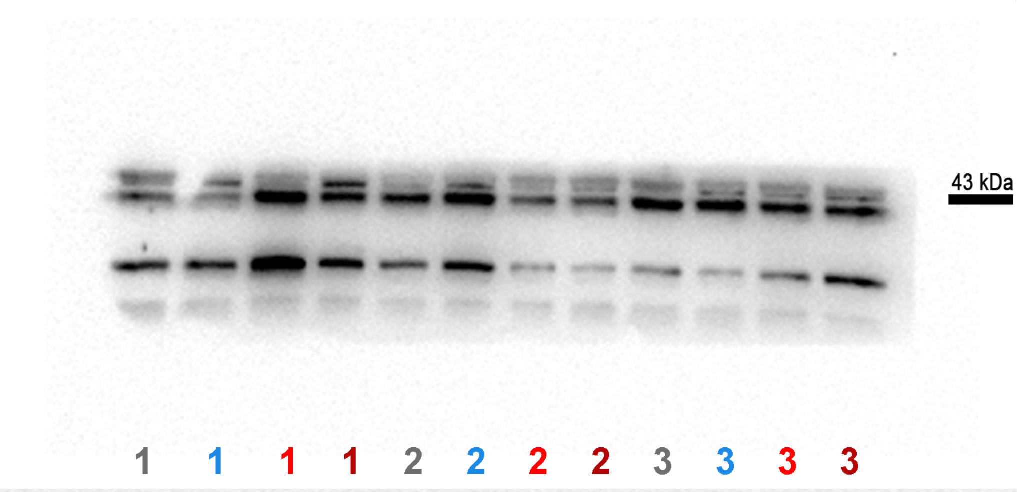

GAPDH

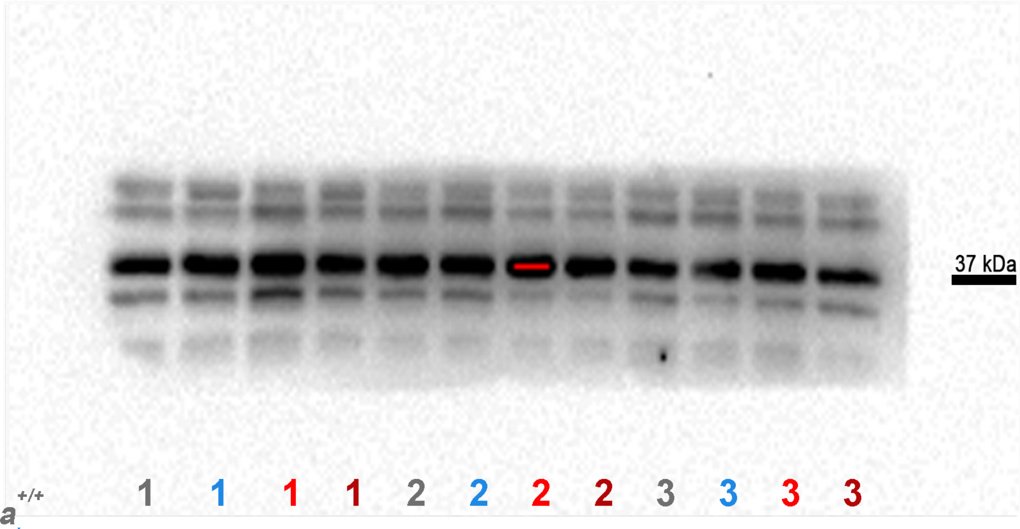

1 - 3 Sham *Grin2a*$^{+/+}$
1 - 3 iTBS *Grin2a*$^{+/+}$
1 - 3 Sham *Grin2a*$^{-/-}$
1 - 3 iTBS *Grin2a*$^{-/-}$

Figure 9A

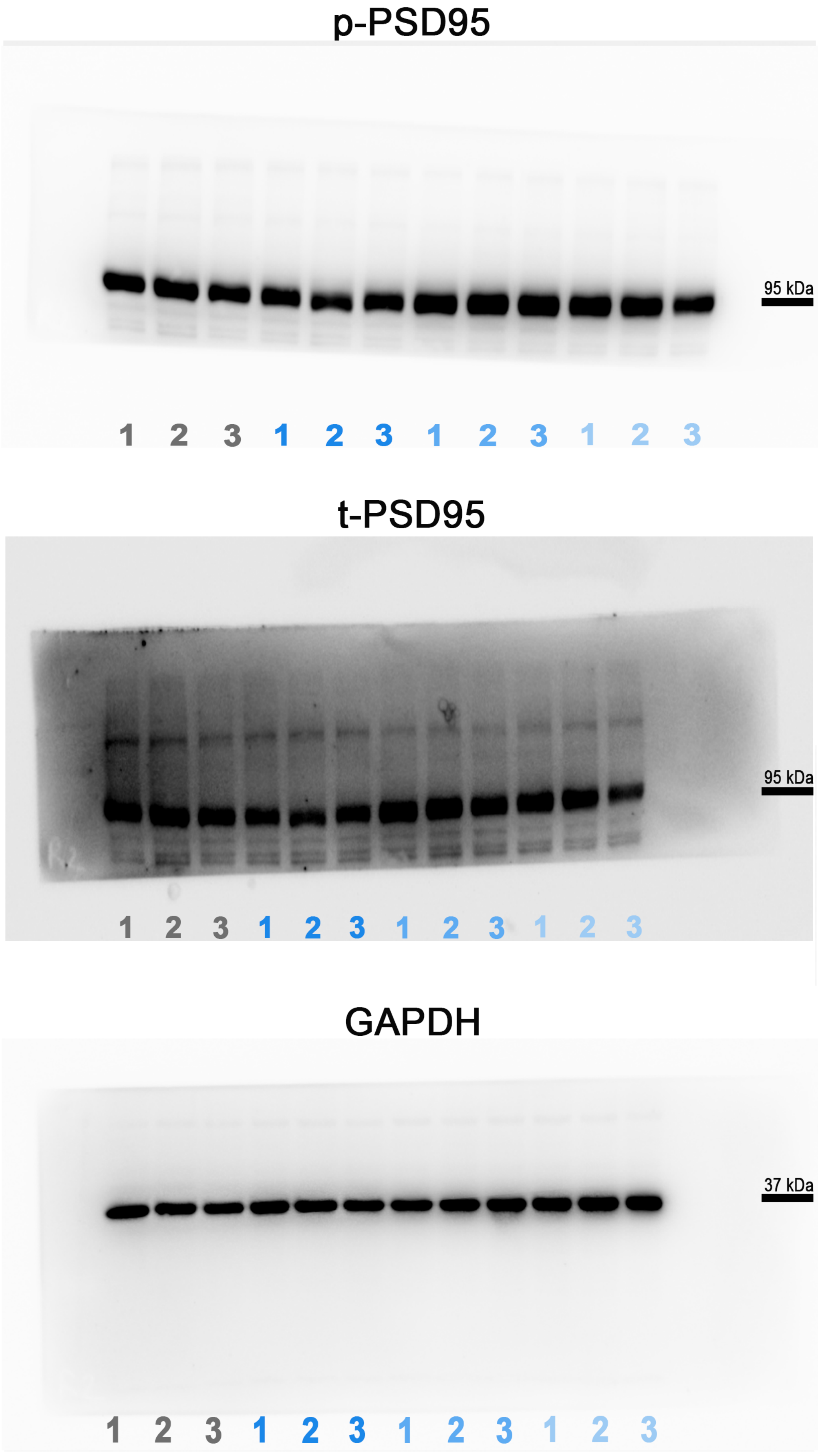

Sample order: Sham 1-3; iTBS 1dps 1-3; iTBS 7dps; iTBS 14dps

Figure 9B

GluN2A

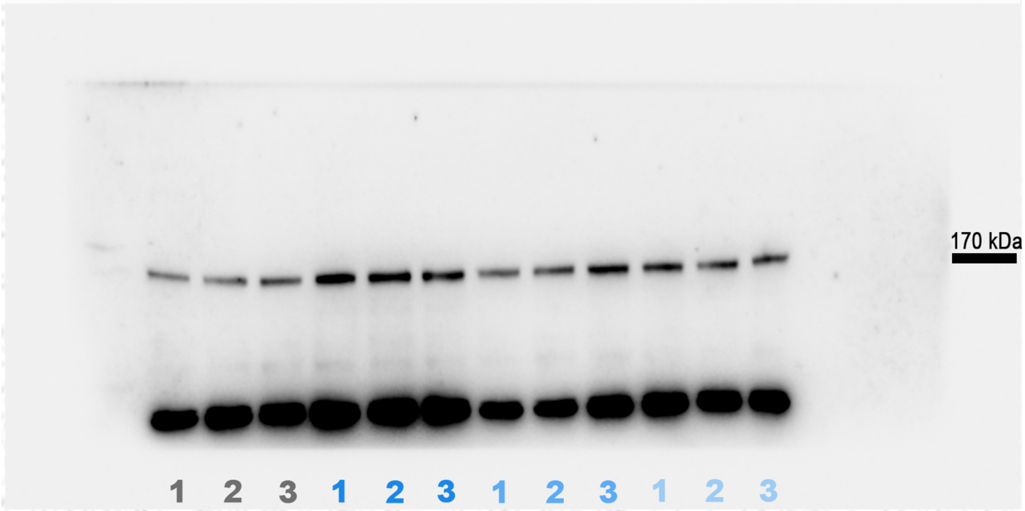

GAPDH

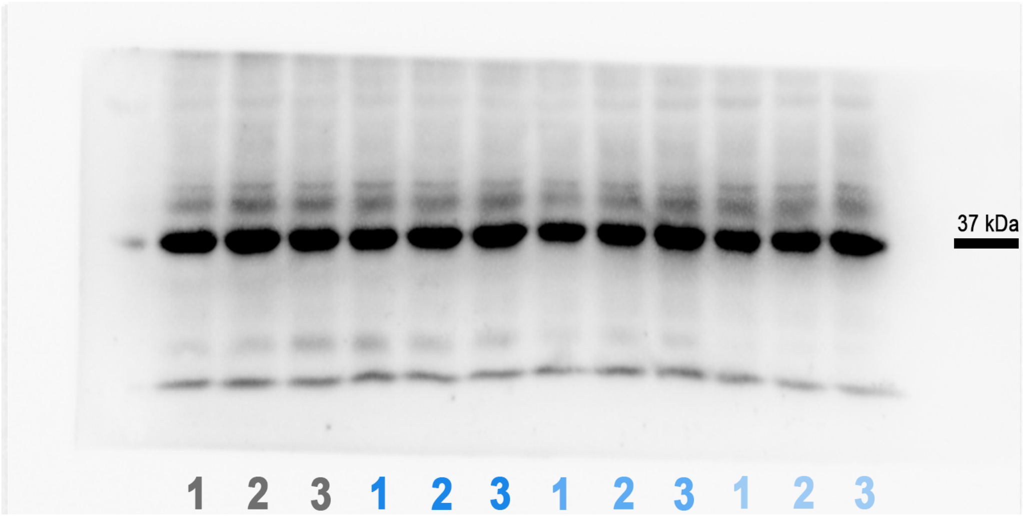

Sample order: Sham 1-3; iTBS 1dps 1-3; iTBS 7dps; iTBS 14dps

Figure 9C

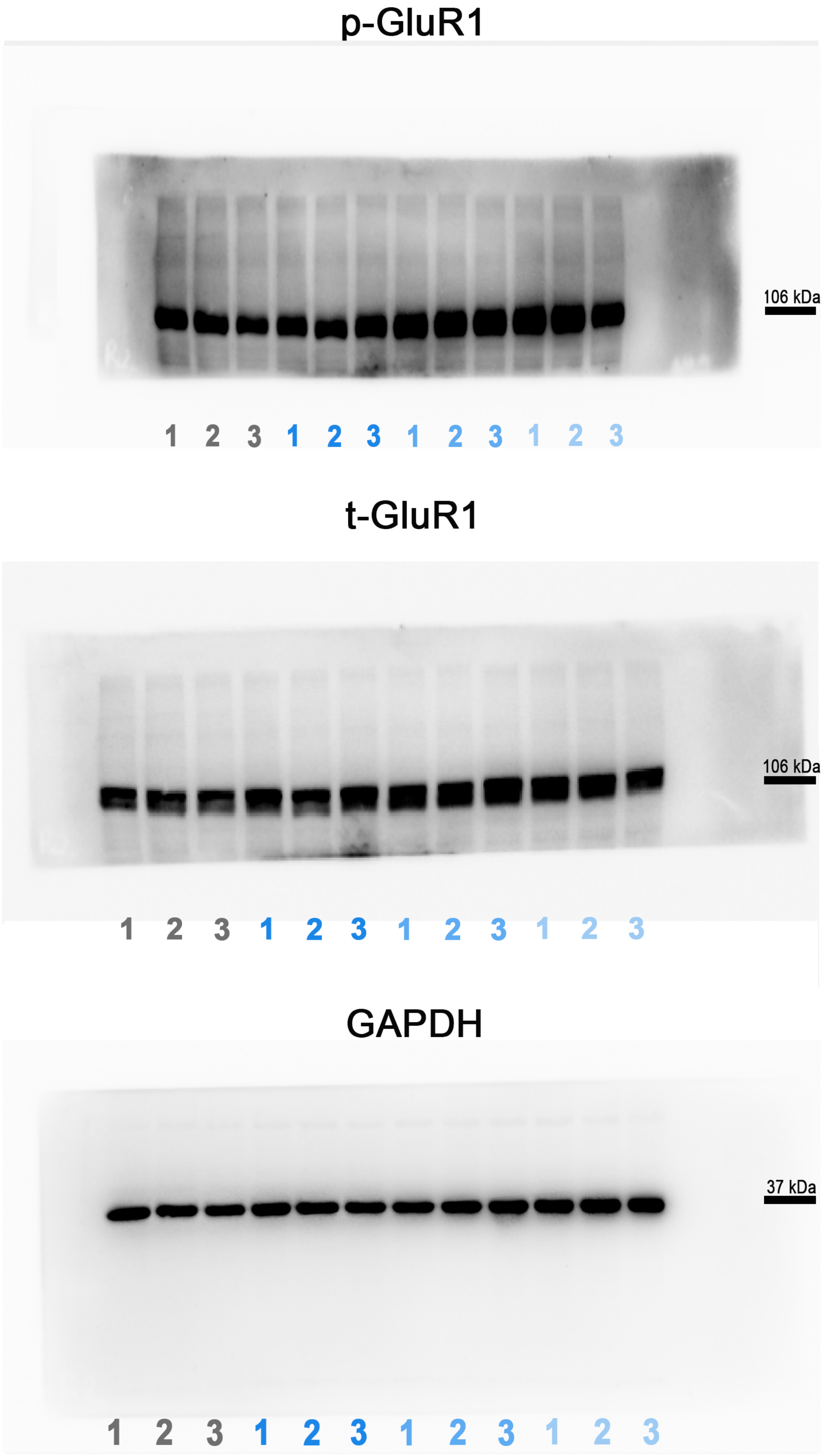

Sample order: Sham 1-3; iTBS 1dps 1-3; iTBS 7dps; iTBS 14dps

Supplementary figure 3C

c-Fos

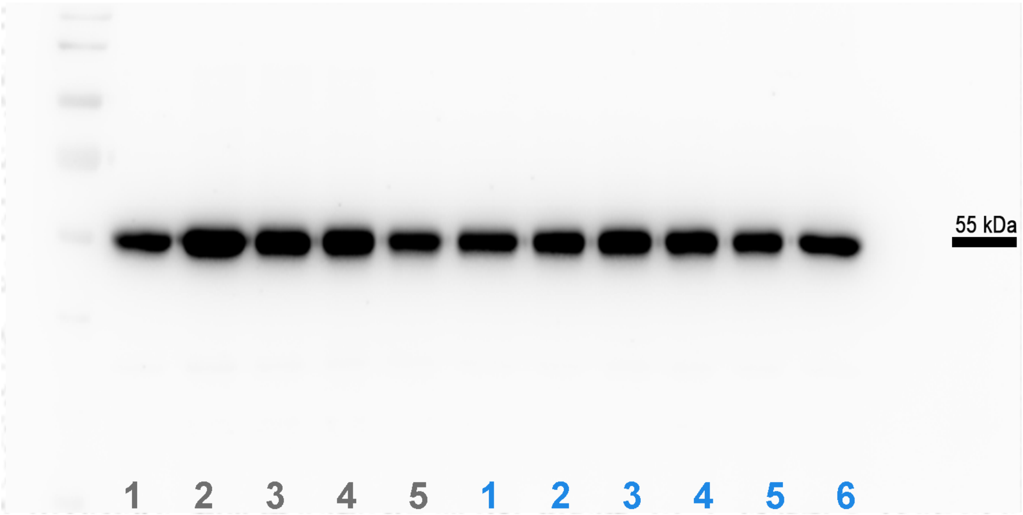

GAPDH

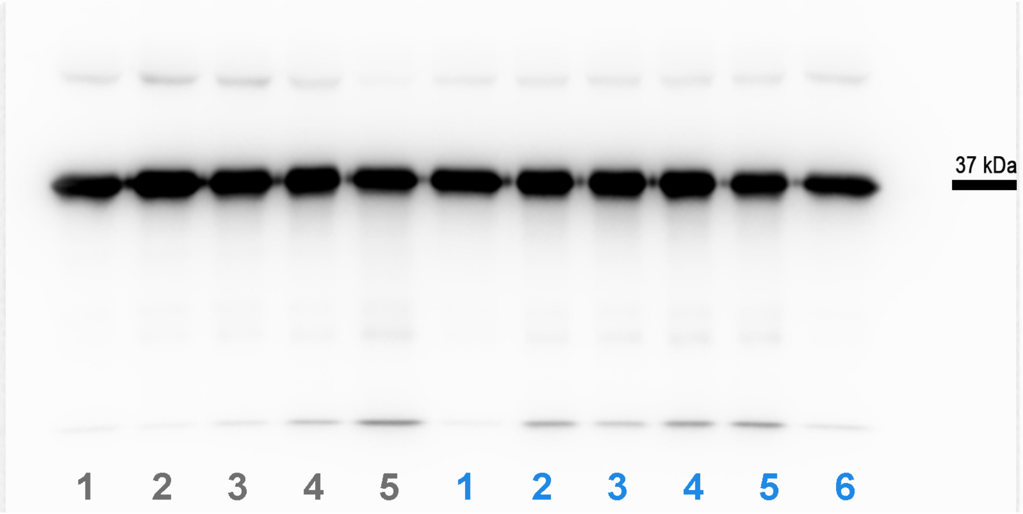

Sample order: Sham 1-5; iTBS 1-6

Supplementary figure 5

Synaptophysin

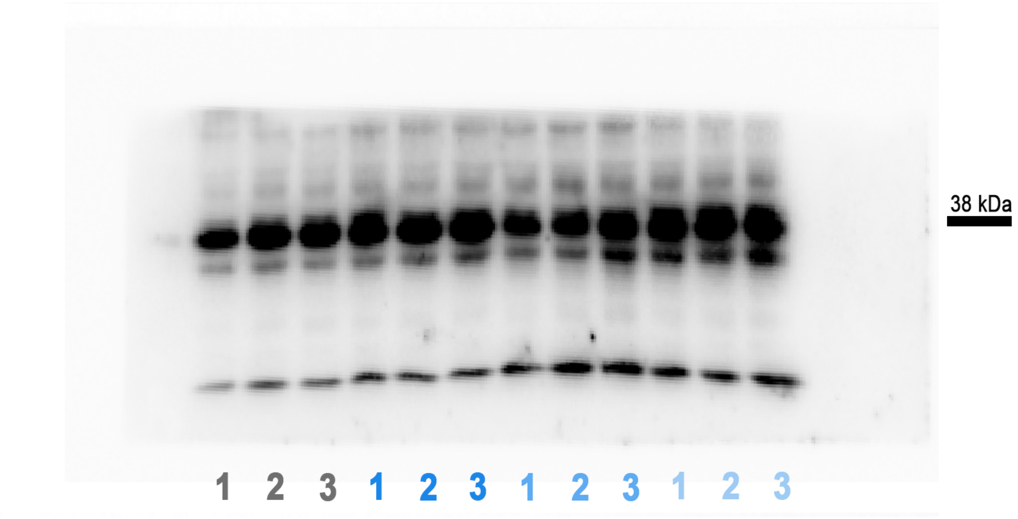

GAPDH

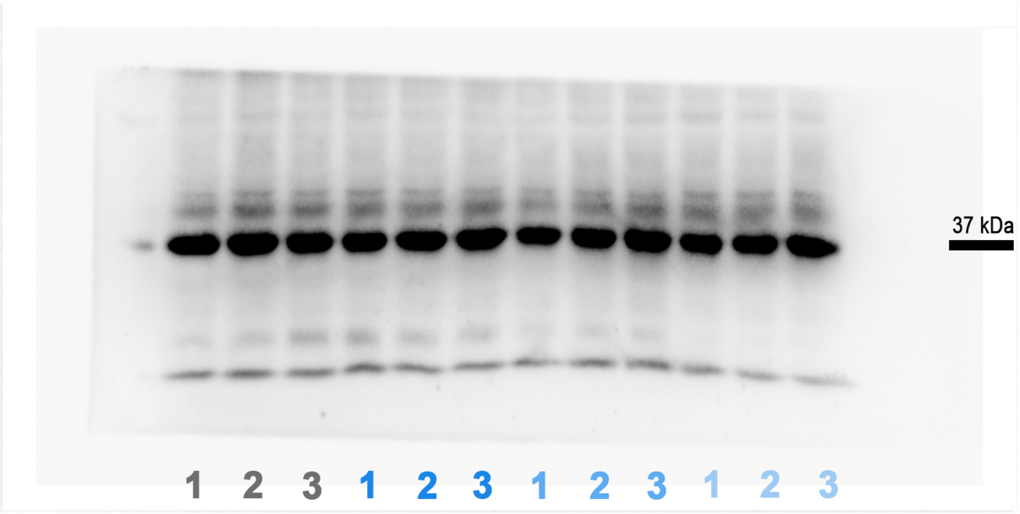

Sample order: Sham 1-3; iTBS 1dps 1-3; iTBS 7dps; iTBS 14dps
